# Reversible control of post-Golgi transport by brefeldin A reveals recycling endosome maturation during glycosylphosphatidylinositol-anchored protein transport

**DOI:** 10.1038/s41467-026-75784-1

**Published:** 2026-07-27

**Authors:** Arata Takiguchi, Ho Tung Shek, Shogo Sasaki, Tatsuya Tago, Taisei Uehara, Yumi Goto, Kiminori Toyooka, Kazuo Kurokawa, Takuro Tojima, Akihiko Nakano, Makoto Maeda, Takunori Satoh, Akiko K. Satoh

**Affiliations:** 1 Graduate School of Integrated Sciences for Life, Hiroshima University, 1-7-1 Kagamiyama, Higashi-Hiroshima, Hiroshima, Japan; 2 Technology Platform Division, Mass Spectrometry and Microscopy Unit, RIKEN Center for Sustainable Resource Science, Yokohama, Kanagawa Japan; 3 Live Cell Super-Resolution Imaging Research Team, RIKEN Center for Advanced Photonics, 2-1 Hirosawa, Wako, Saitama, Japan; 4 Image Processing Research Team, RIKEN Center for Advanced Photonics, Wako, Saitama, Japan; 5 Institute of Integrated Research, Science Tokyo, Chiyoda-ku, Tokyo, Japan; 6 Core Facility Management Center, Hiroshima University, Higashi-Hiroshima, Hiroshima, Japan

**Keywords:** Golgi, Endosomes, Membrane trafficking

## Abstract

Post-Golgi transport plays a crucial role in establishing and maintaining cellular function; however, its mechanism of action remains unclear. Therefore, a system to manipulate post-Golgi transport is highly desirable. In this study, we developed a brefeldin A (BFA)-controlled system to block and restart post-Golgi transport freely, allowing the detailed observation of cargo exit from Golgi stacks using live-cell imaging, electron microscopy, and biochemical analysis. Using this system, glycosylphosphatidylinositol-anchored protein (GPI-AP) transport from the *trans*-Golgi network (TGN) to Golgi-associated recycling endosomes (GA-REs) was visualized. GA-REs expanded during GPI-AP uptake, indicating maturation from the TGN into REs, which later detached as free REs released from the Golgi stacks. Tubular and pearled GPI-AP-positive structures formed on the TGN, which were likely GA-REs transporting GPI-AP from the TGN. REs matured in AP-1-deficient cells, whereas GA-RE detachment was impaired, and long-tubules were observed, thereby delaying GPI-AP delivery to the plasma membrane.

## Abstract (web summary)

The authors developed a new system using brefeldin A and its resistant version of GBF1 knocked in, which can manipulate cargo transport from the trans-Golgi network to recycling endosomes. Details are revealed by live imaging and electron microscopy.

## Introduction

The Golgi apparatus, a key organelle within the secretory pathway, regulates post-translational modification and intracellular lipid, membrane, and secretory protein sorting to their appropriate cellular destinations^1–3^. Its core structural element, the Golgi stack, comprises several flattened cisternae accompanied by various tubules and vesicles^4^. Recycling endosomes (REs) are perinuclear compartments that mediate endocytosed material transport before their return to the plasma membrane^5–7^. The Golgi apparatus and REs are primarily involved in the exocytic and endocytic pathways, respectively; however, each organelle also contributes to the other pathway^8–10^.

Golgi stacks are accompanied with REs on their *trans* side in *Drosophila*, microtubule (MT)-disrupted HeLa cells, and sea urchin embryos^11,12^, indicating that Golgi stacks and REs form integrated structures. REs also exist as free REs independent of Golgi stacks, and both Golgi-associated REs (GA-REs) and free REs are mutually interchangeable. The functional significance of the association between REs and Golgi stacks has been characterized. Glycosylphosphatidylinositol-anchored proteins (GPI-APs) are predominantly localized in GA-REs after their exit from the *trans*-Golgi cisternae. These GA-REs then dissociate from the Golgi stacks, transitioning to free REs while retaining the GPI-AP cargo, a hallmark of post-Golgi transport carriers. In contrast, vesicular stomatitis virus glycoprotein (VSVG) and tumor necrosis factor-alpha (TNFα) are not detected within GA-REs but are found in their immediate vicinity^11,13^.

In *Drosophila*, brefeldin A (BFA)-sensitive *trans*-Golgi network (TGN)-localized ADP-ribosylation factor guanine nucleotide exchange factor (ARFGEF) Sec71 regulates RE detachment from Golgi stacks and post-Golgi transport^14^. As Sec71 is the only BFA-sensitive ARFGEF in *Drosophila*, post-Golgi transport can be manipulated by adding and washing out BFA. The ability to restart post-Golgi transport at any time is highly advantageous, as it allows for detailed observation using live-cell imaging, electron microscopy, and biochemical analysis. However, even though methods for synchronous cargo release are available, technical limitations such as inefficient drug delivery and difficulty in live super-resolution imaging make it challenging to study cargo transport in *Drosophila* compared to cultured mammalian cells^15–19^. Therefore, the development of a system that allows similar manipulations of post-Golgi transport in mammalian cell culture is urgently required.

**Fig. 1:**
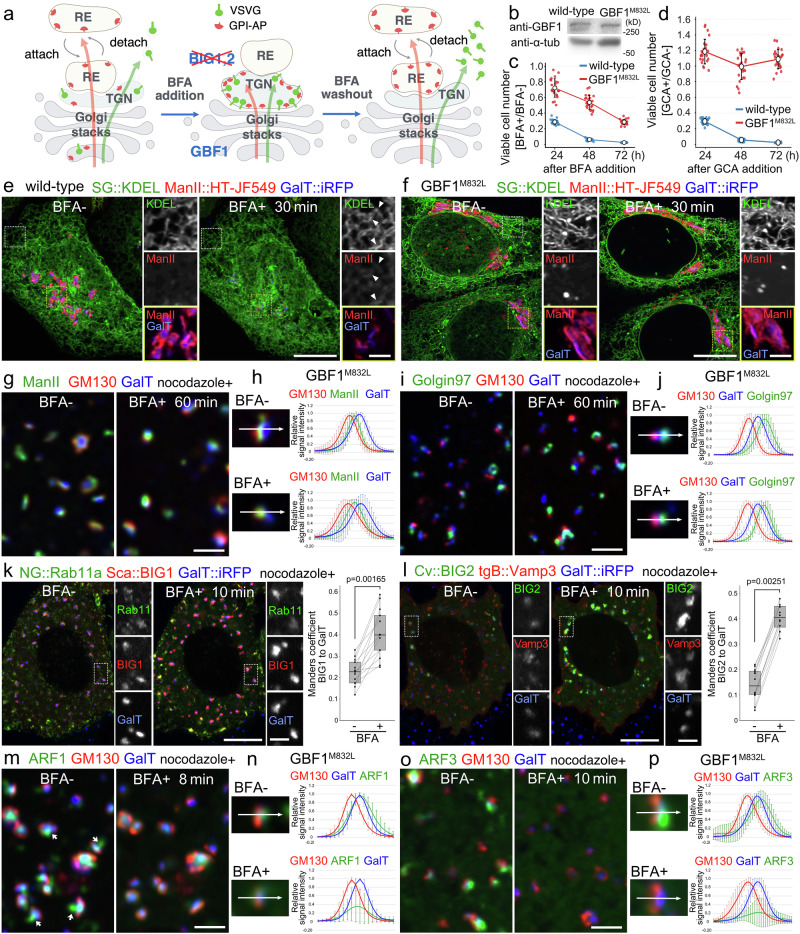
Genomically engineered GBF1 brefeldin A (BFA)-resistant cells. **a** Model for the manipulation of post-Golgi transport using BFA and GBF1^M832L^ cells. BFA administration inhibits the function of *trans*-Golgi network (TGN)-localized ADP-ribosylation factor guanine nucleotide exchange factor (ARFGEF) BIG1/2, resulting in the blockade of post-Golgi transport. Post-Golgi transport resumes after BFA is washed out. **b** Immunoblot detection of endogenous GBF1 and α-tubulin in the wild-type (left) and GBF1^M832L^ cells (right). **c**,** d** Survivability of wild-type (blue) and GBF1^M832L^ cells (red) after BFA (**c**) or Golgicide A (GCA) (**d**) administration. The relative numbers of BFA- or GCA-treated cells were plotted against those of BFA- and GCA-untreated cells at 24, 48, and 72 h after treatment. 24 wells were used for each condition. Error bars represent the mean ± standard deviation (SD). **e**,** f** Golgi apparatus before (left) and 30 min after (right) BFA administration in wild-type (**e**) and GBF1^M832L^ cells (**f**). The images show the ER marker (StayGold::KDEL) in green, the *medial*-Golgi marker (ManII::Halo7-tag-Janelia Fluor 549) in red, and the *trans*-Golgi marker (GalT::iRFP713) in blue. The upper two images in the right panels show magnified black-and-white single-channel images of the white insets, and the bottom images outlined in yellow show magnified double-color images of the yellow insets. Seven cells for wild-type and Seven cell for GBF1^M832L^ cells were observed. **g**–**j** Golgi stacks in nocodazole-treated GBF1^M832L^ cells before (left) and 60 min after (right) BFA administration (**g**,** i**). The images show Ruby::GM130 in red, GalT::iRFP713 in blue, and ManII::EGFP (**g**) or a TGN marker (Golgin97::Clover) (**i**) in green. The plots show the normalized means of the 15 Golgi/TGN marker line profiles across the Golgi stack. Signal intensity was measured along the arrows (representing 1.5 μm). Error bars represent the mean ± SD. The image on the left shows a typical Golgi stack (**h**,** j**). **k**,** l** BIG1 (**k**) and BIG2 (**l**) localization 10 min after BFA administration in nocodazole-treated GBF1^M832L^ cells. Magnified black-and-white single-channel images of the insets are shown in the right panels. Scarlet::BIG1 is shown in red, GalT::iRFP713 is shown in blue, and the RE marker, NG::Rab11a, is shown in green (**k**). Clover::BIG2 is shown in green, GalT::iRFP713 in blue, and the RE marker tgB::Vamp3 in red (**l**). The plots on the right show the Mandars coefficient of BIG1/2 to GalT before (-) and after (+) BFA administration. The box plots show the median and the interquartile range (IQR), while whiskers extend to 1.5 times the interquartile range. Minimum or maximum values are used instead when whiskers exceed the minimum or maximum. Statistical analyses were performed using a two-sided paired Wilcoxon signed-rank test. Thirteen cells were observed BIG1 (**k**) and 12 cells were observed in BIG2 (**l**). **m**–**p** ARF1/3 localization before (left) and 8 or 10 min after (right) BFA administration in nocodazole-treated GBF1^M832L^ cells (**m**–**p**). The images show Ruby::GM130 in red, GalT::iRFP713 in blue, and ARF1::NeonGreen (**m**) or ARF3::NeonGreen in green (**o**). The plots show the normalized mean values for 15 ARF and Golgi markers line profiles across the Golgi stack. Signal intensity was measured along the arrow (representing 1.5 μm). Error bars represent the mean ± SD. The left image shows a typical Golgi stack (**n**, **p**). The arrows indicate ARF1 localization on the strong puncta beyond the *trans*-Golgi cisternae. Scale bars: 10 μm (**e**, **f**), 2 μm (insets of **e**, **f**), 2 μm (**g**, **i**), 10 μm (**k**, **l**), 2 μm (insets of **k**, **l**), and 2 μm (**m**, **o**).

BIG1 and BIG2 are human orthologs of the *Drosophila* Sec71 protein. In cultured human cells, BFA inhibits the *cis*-localized ARFGEF GBF1 as well as BIG1 and BIG2^20,21^. Inhibiting GBF1 causes the Golgi apparatus to be absorbed into the endoplasmic reticulum (ER)^22–26^, preventing BFA from modulating post-Golgi transport in wild-type cells. Therefore, in this study, a BFA-resistant GBF1 allele, GBF1^M832L^, was introduced into HeLa cells: this cell line allows the manipulation of post-Golgi transport by BFA (Fig. 1a). Using this system, we analyzed GPI-AP transport from TGN to GA-RE in detail and found that GPI-AP-positive TGN domains mature into GA-REs.

## Results

### Genomically transformed BFA-resistant ARFGEF GBF1, GBF1^M832L^, counteracts the effects of Golgicide A and BFA on GBF1

CRISPR/Cas9-mediated knock-in was performed to convert GBF1 from BFA-sensitive to -resistant, and a single allelic GBF1^M832L^ HeLa cell line was cloned. The levels of GBF1 protein were comparable between the wild-type and GBF1^M832L^ cells (Fig. 1b). The viability of GBF1^M832L^ cells was compared with that of wild-type cells treated with or without 10 µM BFA (Fig. 1c). GBF1^M832L^ cells were more resistant to BFA than the wild-type cells. However, the surviving cells were fewer than that in the absence of BFA, likely because BIG1/2 remained sensitive to BFA and reduced the viability of GBF1^M832L^ cells. As the GBF1-specific inhibitor Golgicide A (GCA) binds to wild-type GBF1 but not GBF1^M832L^^27^, we investigated the effect of GCA on the viability of the wild-type and GBF1^M832L^ cells. Cell viability was comparable to that of untreated cells when GBF1^M832L^ cells were treated with 10 µM GCA (Fig. 1d). Therefore, the presence of a single GBF1^M832L^ allele sufficiently counteracted the effects of GCA and BFA on GBF1.

### BFA does not induce Golgi stack absorption into the ER in GBF1^M832L^ cells

In wild-type cells, the *cis*-Golgi marker GM130, *medial*-Golgi marker ManII, and *trans*-Golgi marker GalT relocated to the ER after BFA administration (Fig. 1e and Supplementary Video 1)^23,28–33^. In contrast, these markers did not relocate to the ER and remained largely intact in GBF1^M832L^ cells; however, the Golgi morphology was slightly perturbed (Fig. 1f and Supplementary Video 1), similar to BIG1/2 function impairments^20,30,34,35^. Similar to HeLa cells with the GBF1^M832L^ mutation, BFA administration to HEK293T cells with the GBF1^M832L^ mutation did not result in relocation of the *trans*-Golgi marker GalT to the ER (Supplementary Video 1). MT-disrupted GBF1^M832L^ HeLa cells, which have scattered Golgi stacks in front of each ER exit site (ERES)^36,37^ instead of the Golgi ribbon, were used to further investigate the *cis–trans* polarity of the Golgi apparatus. GM130, ManII, and GalT were aligned in a *cis*-to-*trans* order, even in BFA-treated GBF1^M832L^ cells (Fig. 1g, h). The relative spatial distribution of GM130, GalT, and golgin97 was also very similar in BFA-treated and -untreated GBF1^M832L^ HeLa cells (Fig. 1i, j). Moreover, although BIG1/2 were weakly associated with the Golgi/GA-RE and free REs before BFA administration, the strong BIG1/2 signals were detected on both Golgi/GA-REs and free REs after BFA administration (Fig. 1k, l), consistent with the stabilized membrane association of a complex between BFA, ARF-GDP, and ARFGEF^14,24,26^.

### BFA impairs ARF1 and ARF3 membrane recruitment in GBF1^M832L^ cells

The membrane association of Golgi-localizing ARFs (ARF1, ARF3, ARF4, and ARF5) was examined before and after BFA administration. In MT-intact GBF1^M832L^ cells, all four ARFs were localized to Golgi stacks and cytoplasmic foci before BFA administration. ARF1 and ARF3 co-localized with the RE marker TfR, but not with the ERES marker Sec13, in these puncta. In contrast, ARF4 and ARF5 co-localized with Sec13, but not with TfR (Supplementary Fig. 1a–e). Thus, in addition to the Golgi stacks, ARF1 and ARF3 are localized in the RE, and ARF4 and ARF5 are localized in the ERES, which are consistent with the results of previous studies^38,39^. Upon BFA administration, all ARF3 diffused, and ARF1 on the puncta also diffused; however, ARF1 on the Golgi apparatus remained visible. BFA administration did not affect ARF4 and ARF5 localization in the Golgi apparatus and puncta (Supplementary Fig. 1c). Similarly, some ARF1 and most ARF3 diffused rapidly after BFA administration in MT-disrupted GBF1^M832L^ cells (Fig. 1m–p). Before BFA administration, ARF1 was localized in the entire region of the Golgi stacks, and the strong puncta beyond the *trans*-Golgi cisternae (Fig. 1m). Upon BFA administration, ARF1 puncta disappeared, and the Golgi localization was reduced but remained (Fig. 1m, n). Because ARF1 diffused in wild-type cells after BFA administration (Supplementary Fig. 2a), ARF1 remaining on Golgi stacks in GBF1^M832L^ cells is likely activated by GBF1^27,40^. In contrast, ARF4 and ARF5 did not diffuse after BFA treatment in MT-disrupted GBF1^M832L^ cells (Supplementary Fig. 2b, c). These results suggest that ARF1 and ARF3 are prominent targets of BIG1/2, although previous studies have indicated that ARF4 is also localized to the TGN and regulates trafficking from endosomes to the TGN^41,42^.

### RE motility is reduced in BFA-treated GBF1^M832L^ cells

**Fig. 2:**
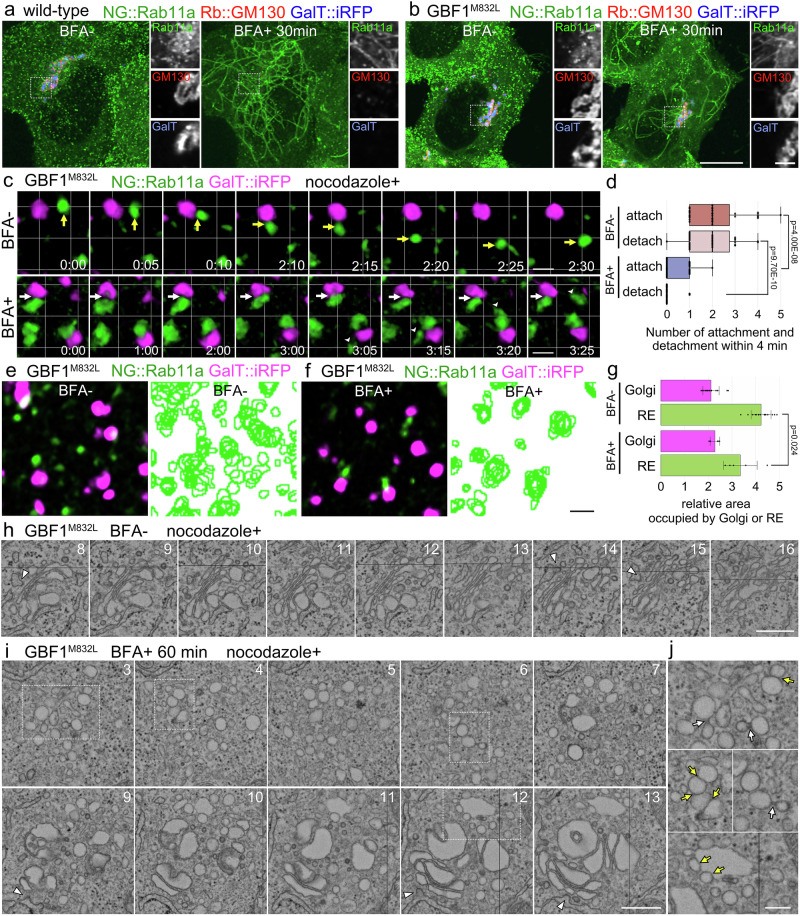
Dynamics of recycling endosomes (REs) in GBF1^M832L^ cells. **a**,** b** Localization of REs in wild-type (**a**) and GBF1^M832L^ cells (**b**) before (left) and 30 min after BFA administration (right). Magnified black-and-white single-channel images of the insets are shown in the right panels. The images show Ruby::GM130 in red, GalT::iRFP713 in blue, and NeonGreen::Rab11a in green. Five cells for wild-type and seven cells for GBF1^M832L^ cells were observed. **c** High-speed time-lapse images showing the dynamics of REs (NeonGreen::Rab11a: green) and Golgi stacks (GalT::iRFP713: magenta) with and without BFA treatment. Images were acquired using super-resolution confocal live-imaging microscopy (SCLIM), and the number in the lower-right corner indicates the elapsed time (min:sec). Yellow arrows indicate a free RE attached to the Golgi stack and then detached from it. White arrows indicate a RE stably associated with the Golgi stack. Arrowheads show the free RE moving from left to right in the image field. The following numbers of cells were analyzed: BFA-: *n* = 8, BFA + : *n* = 7. **d** The plot shows the number of RE attachment to and detachment from Golgi stacks within 4 min in BFA-treated and -untreated GBF1^M832L^ cells. The box plots show the median and the IQR, while whiskers extend to 1.5 times the interquartile range. Minimum or maximum values are used instead when whiskers exceed the minimum or maximum. Statistical analyses were performed using a two-sided Welch’s *t* test with Holm adjustment for multiple comparisons. Thirty Golgi stacks were counted for both BFA − and BFA + conditions. **e**,** f** Dynamics of REs in GBF1^M832L^ cells before (**e**) and after (**f**) BFA administration. The images on the left are frames from live imaging of GalT::iRFP713 (magenta) and NG::Rab11a (green). The right drawings are overlaid with traces of 11 RE positions at 15-second intervals. **g** The relative areas occupied by overlaid traces of 13 REs or Golgi positions at 10 s intervals are shown against the mean area of a single wild-type Golgi or RE trace. Error bars represent the mean ± SD. Statistical significance was assessed using a one-sided two-sample *t* test assuming unequal variances (Welch’s* t* test). The following numbers of area were analyzed: BFA-: *n* = 18, BFA + : *n* = 5. **h**–**j** Scanning electron micrographs of serial sections of a Golgi stack with a 50-nm interval in a GBF1^M832L^ cell after 60 min of incubation with (**i**,** j**) or without (**h**) 10 μM BFA. The number in the top-right corner indicates the frame in Supplementary Video 3. Magnified images of the insets in (**i**) are shown in (**j**) for BFA-treated GBF1^M832L^ cells. Arrowheads indicate the ERES. Yellow arrows indicate the hemifused vesicles. White arrows indicate constricted connections between the vesicles. The following numbers of cells were analyzed: BFA-: *n* = 19 (TEM) and 4 (SEM), BFA + 60 min: *n *= 15 (TEM) and 9 (SEM). Scale bars: 10 μm (**a**, **b**), 2 μm (insets of **a**, **b**), 1 μm (**c**, **e**,** f**), 500 nm (**h**, **i**), and 200 nm (**j**).

BFA induces the formation of a tubular network of TGN and endosomes, as well as Golgi absorption into the ER^43–45^. Golgi absorption is caused by the loss of GBF1 function^14,24^, whereas tubular network formation of TGN and endosomes is induced by the functional loss of BIG1/2^35,46^. In coincidence with BFA-sensitivity of BIG1/2 in GBF1^M832L^ cells, Rab11a-positive tubules appeared upon BFA administration, similar to the wild-type cells (Fig. 2a, b and Supplementary Video 1); before BFA administration, Rab11a was localized near the Golgi apparatus or in small tubules and foci in the cytoplasm. Therefore, BFA alters the regulation of the endosomal system via BIG1/2 in GBF1^M832L^ cells.

The dynamics of REs and Golgi stacks in MT-disrupted GBF1^M832L^ cells with or without BFA were observed using super-resolution confocal live imaging microscopy (SCLIM)^47–49^. BFA did not affect the presence of GA-REs or free REs; however, RE motility was greatly reduced in BFA-treated GBF1^M832L^ cells (Supplementary Video 2). Before BFA administration, free REs moved within the cytoplasm and frequently attached to and separated from Golgi stacks (Fig. 2c). However, the movement of free REs mostly ceased after BFA administration, and GA-REs did not detach from and stably associate with Golgi stacks. The frequency of RE attachment to and detachment from Golgi stacks was significantly reduced (Fig. 2c, d), indicating that ARF1 and ARF3 promote RE motility. The relative movement of the Golgi stacks or REs, defined as the total area occupied by the REs in 11 frames, indicated that RE movement, but not Golgi movement, was reduced after BFA administration (Fig. 2e–g).

Reduced RE motility has not previously been reported in BIG1/2 deficiency. Therefore, we next examined RE motility in BIG1/2 siRNA-knockdown cells. Notably, 72 hours after siRNA treatment, the amount of BIG1/2 protein decreased to less than 19% of the wild-type, and we found Rab11-positive tubular network were often formed in BIG1/2-double knockdown (DKD) cells (Supplementary Fig. 3a, b). Observation of the dynamics of REs and Golgi stacks in MT-disrupted BIG1/2-DKD cells using SCLIM (Supplementary Fig. 3c) revealed that the REs were stably attached to the Golgi stacks. Quantification of the RE movement showed a significant reduction in motility (Supplementary Fig. 3d, e). These results indicate that RE motility requires BIG1/2 activity, and the reduction of RE motility in BFA-treated GBF1^M832L^ cells is a direct consequence of BIG1/2 inhibition.

### Vesicle accumulation on the *trans* side of Golgi stacks in BFA-treated GBF1^M832L^ cells

The membrane structures of the Golgi stacks in MT-disrupted GBF1^M832L^ cells were observed in serial thin sections (Fig. 2h–j, and Supplementary Video 3). The Golgi stacks consisted of several cisternae and vesicles in BFA-untreated GBF1^M832L^ cells (Fig. 2h). In contrast, BFA treatment enlarged Golgi stacks and induced the accumulation of several vesicles or cisternae on the *trans* side (Fig. 2i). Some vesicles and cisternae attached in a hemifused state; the electron density and membrane thickness between the attached vesicles appeared similar to those of the other membranes on the same vesicles, suggesting that they were single-bilayer membranes (Fig. 2j). Vesicles were also connected by small bridges, whose cytoplasmic sides exhibited high electron density (Fig. 2j), suggesting defects in membrane fission. Similar accumulation of vesicle clusters and hemifused vesicles was observed in MT-disrupted BIG1/2-DKD cells (Supplementary Fig. 3f).

To identify REs in MT-disrupted GBF1^M832L^ cells that had been treated or untreated with BFA, a genetically encoded EM-tagged ascorbate peroxidase 2 (APEX2)^50,51^ was fused with an RE marker, TfR (TfR::APEX2), and expressed. APEX2 signals were detected in tubules or vesicles on the *trans* side of Golgi stacks (Supplementary Fig. 4a, b and Supplementary Video 4). Occasionally, cisternae associated with the Golgi stacks were positive for TfR::APEX2 (Supplementary Fig. 4c). Conversely, cisternal association of TfR::APEX2 was not observed in BFA-treated, MT-disrupted GBF1^M832L^ cells; however, some of the accumulating vesicles, including those in the hemifused state, exhibited APEX2 signals (Supplementary Fig. 4d, e and Supplementary Video 4). Therefore, some accumulating vesicle clusters and hemifused vesicles were identified as REs.

### BFA strongly delays cargo transport in MT-intact cells

**Fig. 3:**
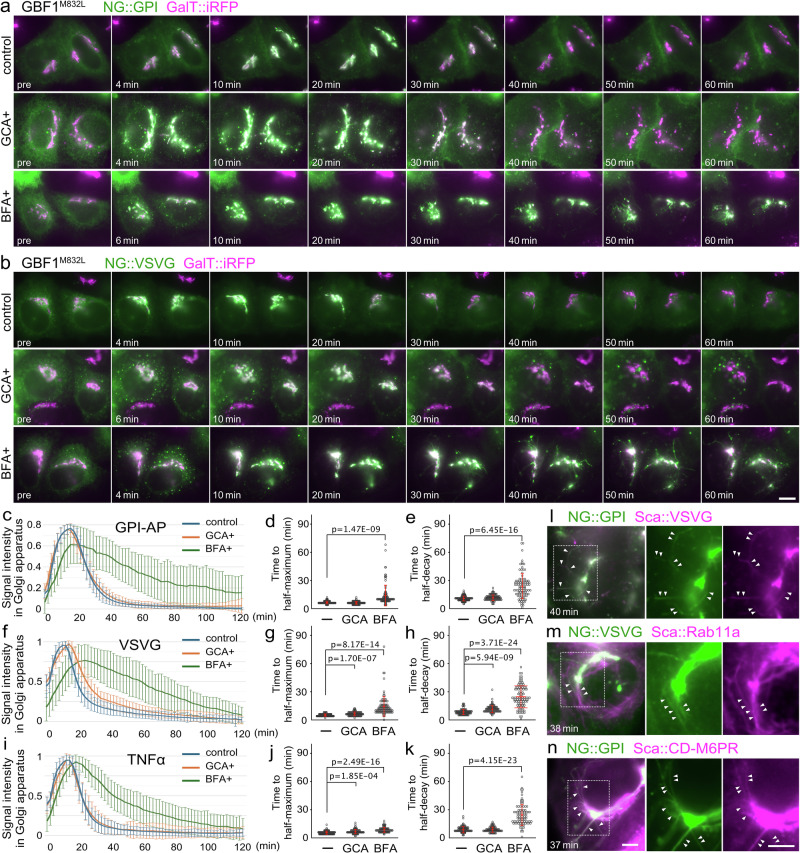
Cargo transport is delayed in BFA-treated GBF1^M832L^ cells. **a**,** b** Time-lapse images of NeonGreen::GPI (**a**) and NeonGreen::VSVG (**b**) transport initiated by the BME-RUSH system in untreated (top), GCA-treated (middle), and BFA-treated cells (bottom). NeonGreen::GPI (**a**) and NeonGreen::VSVG (**b**) are shown in green, and GalT::iRFP713 is shown in magenta. **c**–**k** Quantification of cargo transport kinetics: **c**,** f**,** i** Plots of the relative signal intensity of NeonGreen::GPI (**c**), NeonGreen::VSVG (**f**), and TNFα::NeonGreen (**i**) in the Golgi apparatus over time after BME administration. Blue, orange, and green lines represent untreated, GCA-treated, and BFA-treated cells, respectively. The Golgi apparatus is defined by a GalT::iRFP713 signal. The following numbers of cells were analyzed: GPI-AP (control): *n* = 101, GPI-AP (GCA + ): *n* = 100, GPI-AP (BFA + ): *n* = 99, VSVG (control): *n* = 94, VSVG (GCA + ): *n* = 101, VSVG (BFA + ): *n* = 97, TNFα (control): *n *= 99, TNFα (GCA + ): *n* = 85, TNFα (BFA + ): *n *= 90. Error bars represent the mean ± SD. **d**,** g**,** j** Time constants for cargo accumulation in the Golgi apparatus. The time constants for NeonGreen::GPI (d) without drug administration, with 10 µM of GCA administration, and with 10 µM BFA administration were 6.5 ± 1.4, 6.1 ± 1.5, and 13.9 ± 11.0 min, respectively. The time constants for NeonGreen::VSVG (g) without drug administration, with 10 µM GCA, and with 10 µM BFA were 5.0 ± 1.3, 6.4 ± 2.1, and 14.3 ± 10.5 min, respectively. The time constants for TNFα::NeonGreen (j) without drug administration, with 10 µM GCA, and with 10 µM BFA were 5.6 ± 1.7, 6.8 ± 2.4, and 8.6 ± 2.6 min, respectively. Values are mean ± SD; error bars indicate SD. Statistical analysis was performed using a one-sided two-sample *t* test assuming unequal variances (Welch’s *t* test). **e**, **h**, **k** Time constants for cargo exit from the Golgi apparatus. The time constants for NeonGreen::GPI (**e**) without drug administration, with 10 µM GCA, and with 10 µM BFA were 10.8 ± 2.6, 11.5 ± 3.0, and 23.8 ± 13.3 min, respectively. The time constants for NeonGreen::VSVG (**h**) without drug administration, with 10 µM GCA, and with 10 µM BFA were 8.4 ± 2.9, 11.3 ± 3.7, and 24.5 ± 11.7 min, respectively. The time constants for TNFα::NeonGreen (**k**) without drug administration, with 10 µM GCA, and with 10 µM BFA were 8.7 ± 3.3, 8.8 ± 2.8, and 24.5 ± 11.3 min, respectively. Values are mean ± SD; error bars indicate SD. Statistical analysis was performed using a one-sided two-sample *t* test assuming unequal variances (Welch’s *t* test). **l**–**n** Co-localization studies in BFA-treated cells: **l** NeonGreen::GPI (green) and Scarlet::VSVG (magenta) localization 40 min after BME administration; **m** NeonGreen::VSVG (green) and Scarlet::Rab11a (magenta) localization 38 min after BME administration; **n** NeonGreen::GPI (green) and Scarlet::CD-M6PR (magenta) localization 37 min after BME administration. The right panels display magnified single-color images, which are shown in the insets of the left panels. Arrowheads indicate the co-localization of cargo pairs (**l**, **n**) or cargo with Rab11a (**m**). The following numbers of cells were analyzed: NeonGreen::GPI vs Scarlet::VSVG: *n* = 2, NeonGreen::VSVG vs Scarlet::Rab11a: *n* = 3, NeonGreen::GPI vs Scarlet::CD-M6PR: *n* = 2. Scale bars: 10 μm (**a**, **b**) and 5 μm (**l**–**n**).

The effects of GCA and BFA on the transport of three cargo proteins—GPI-AP, VSVG, and TNFα—were investigated in GBF1^M832L^ cells using the BME-RUSH system^52,53^. After the addition of biotin methyl ester (BME) and without GCA or BFA, all three cargoes reached their maximum levels and exited the Golgi apparatus after approximately 10 and 30 min, respectively (Fig. 3a–k, Supplementary Fig. 5a and Supplementary Video 5). Administering 10 µM GCA to cells before cargo release did not alter the kinetics of GPI-AP transport; however, it weakly affected VSVG and TNFα transport. Consistent with the delay, VSVG and TNFα accumulated in cell peripheral dots, likely ERESs, before reaching the Golgi apparatus in GCA-treated GBF1^M832L^ cells. GBF1^M832L^ cells harbor one wild-type allele whose product forms a dead-end complex with GCA and ARF1, exerting a dominant-negative effect on the GBF1^M832L^ allele upon exit from the ERESs.

Unlike GCA, 10 µM BFA treatment strongly influenced all three cargo transports. The time constants for the exit from the Golgi apparatus were dramatically delayed (Fig. 3a–k, Supplementary Fig. 5a and Supplementary Video 5), consistent with BFA-induced BIG1/2 inhibition failing in recruiting ARF1/3 on the *trans* side of the Golgi stacks (Fig. 1m–p and Supplementary Fig. 1). Some delays were also observed in early Golgi transport, which may be caused by the substantial delay in cargo export from the Golgi apparatus. Similar to GCA treatment, peripheral punctate VSVG and TNFα signals on ERES were visible in BFA-treated cells. The most striking phenomenon observed in BFA-treated cells was the formation of numerous tubules simultaneously carrying newly synthesized GPI-AP, VSVG, and catio-dependent-mannose 6-phosphate receptors (CD-M6PRs), which were sometimes Rab11-positive (Fig. 3l–n and Supplementary Fig. 5b–d).

Without BFA, GPI-AP, but not VSVG or TNFα, is transported into Rab11a-positive, GA-REs before its transport to the plasma membrane^11,13^. To confirm these results, the transport of two cargo types was initiated simultaneously using the BME-RUSH system in MT-disrupted cells. GPI-AP and VSVG were separated in the TGN and post-Golgi compartments; however, VSVG and TNFα continued to co-localize (Supplementary Fig. 5e–g, and Supplementary Video 6). CD-M6PR was transported to endosomes rather than the plasma membrane in the wild-type cells^54^. Therefore, GPI-AP, VSVG/TNFα, and CD-M6PR likely use different transport carriers under normal conditions. Consequently, post-Golgi cargo transport using tubules in BFA-treated GBF1^M832L^ cells appears to lack selectivity.

### BFA blocks cargo transport in MT-disrupted cells

**Fig. 4:**
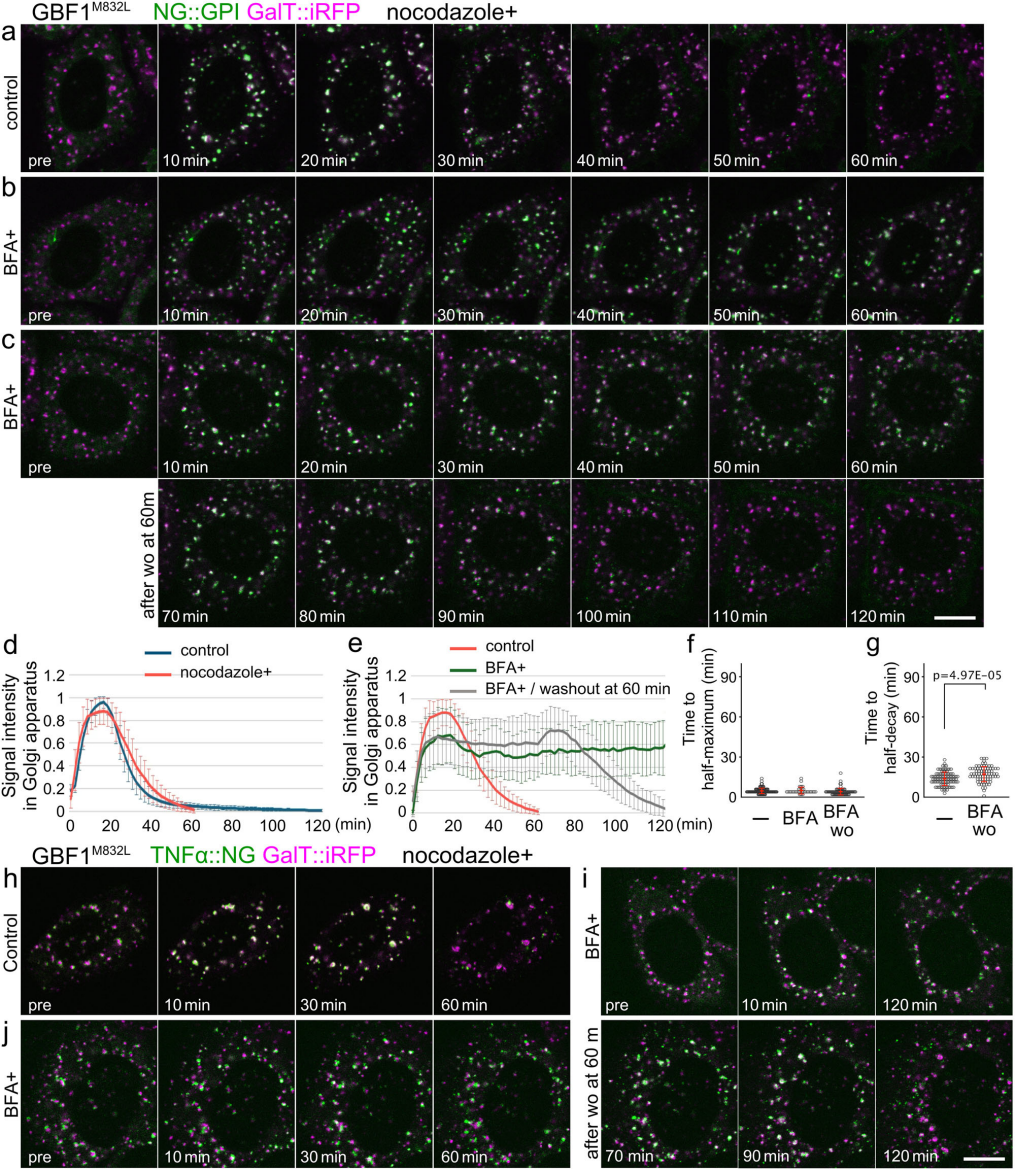
BFA blocks cargo exit from Golgi stacks in nocodazole-treated GBF1^M832L^ cells. **a**–**c** Time-lapse images showing NeonGreen::GPI transport initiated by the BME-RUSH system in untreated (**a**), BFA-treated (**b**), and BFA-treated and washed-out cells (**c**). BFA and BME treatments were initiated after a 4 h incubation with nocodazole. BFA was washed out 60 min after the addition of BME (**c**, bottom). NeonGreen::GPI is shown in green, and GalT::iRFP713 is shown in magenta. **d**–**g** Quantification of cargo transport kinetics. **d**,** e** Plots of relative signal intensity of GPI-AP in the Golgi apparatus over time after BME administration. Blue and orange lines represent untreated and nocodazole-treated cells, respectively (**d**). Orange, green, and gray lines represent untreated, BFA-treated, and BFA-treated cells that have undergone washout, respectively (**e**). The Golgi apparatus was defined by a GalT::iRFP713 signal. The following numbers of cells were analyzed: GPI-AP (control: BFA-): *n* = 100, GPI-AP (BFA + ): *n* = 33, and GPI-AP (BFA-washout): *n* = 76.　 Error bars represent the mean ± SD. **f** Time constants for NeonGreen::GPI accumulation in the Golgi stacks. The time constants without BFA administration, with 10 µM BFA, and BFA washout were 4.8 ± 2.1, 5.0 ± 2.6 min, and 4.2 ± 2.5, respectively. Values are mean ± SD; error bars indicate SD. **g** Time constants for NeonGreen::GPI exit from the Golgi stacks. The time constants without BFA and after washout of 10 µM BFA were 13.8 ± 5.1 and 17.3 ± 5.6 min, respectively. Error bars represent the mean ± SD. Statistical analysis was performed using a one-sided two-sample* t* test assuming unequal variances (Welch’s *t* test). **h**–**j** Time-lapse images showing TNFα::NeonGreen transport initiated by the BME-RUSH system in untreated cells (**h**), BFA-treated cells (**i**), and cells after BFA washout (**j**). BFA and BME treatments were initiated after a 4-hour incubation with nocodazole. BFA was washed out 60 min after the addition of BME (j, bottom). TNFα::NeonGreen is shown in green, and GalT::iRFP713 is shown in magenta. Scale bars: 10 μm (**a**–**c**, **h**–**j**).

Next, we investigated the effects of BFA on GPI-AP transport in MT-disrupted GBF1^M832L^ cells, because the scattered Golgi stacks allow more detailed analysis with precise *cis–trans* polarity. The effects of BFA on GPI-AP transport in MT-disrupted GBF1^M832L^ cells were examined (Fig. 4 and Supplementary Video 7). The transport kinetics of MT-disrupted GBF1^M832L^ cells without BFA treatment were slightly delayed, but nearly comparable to those of MT-intact GBF1^M832L^ cells (Fig. 3a and Fig. 4a, d). In MT-disrupted BFA-treated GBF1^M832L^ cells, the time constants for GPI-AP accumulation in the Golgi were also comparable to those of MT-disrupted BFA-untreated GBF1^M832L^ cells; however, GPI-AP did not exit the Golgi stack, even 2 h after cargo release from the ER (Fig. 4b, e, f). Thus, the time constant for GPI-AP exit from the Golgi could not be calculated. In MT-intact cells, cargo exit from the Golgi stack was delayed but not blocked by BFA; in contrast, the suppression of TGN-endosome tubule network formation by MT-disruption blocked GPI-AP exit from the Golgi stack. TNFα exit was also blocked in the Golgi stacks (Fig. 4h, i and Supplementary Video 7).

To investigate whether the delay or blockage of cargo transport in BFA-treated GBF1^M832L^ cells is caused by the inhibition of BIG1/2 function, GPI-AP transport was examined in MT-intact and MT-disrupted BIG1/2-DKD cells. Similar to BFA-treated MT-intact GBF1^M832L^ cells, GPI-AP transport was significantly delayed in MT-intact BIG1/2-DKD cells (Fig. 3a, c–e and Supplementary Fig. 6a, c, d). In MT-disrupted BIG1/2-DKD cells, GPI-AP transport was also significantly delayed, but not blocked (Supplementary Fig. 6b, e, f). This differs from the situation in BFA-treated, MT-disrupted GBF1^M832L^ cells. We speculated that BIG1/2 inhibition by siRNA was imperfect, allowing residual activity and GPI-AP transport in MT-disrupted BIG1/2-DKD cells. Nevertheless, GPI-AP transport in BIG1/2-DKD cells mostly mimics that in BFA-treated GBF1^M832L^ cells. Therefore, BFA inhibition of BIG1/2 activity partly causes these effects on post-Golgi transport in GBF1^M832L^ cells.

### Cargo transport resumes after BFA washout in MT-disrupted BFA-treated GBF1^M832L^ cells

GPI-AP accumulated in the TGN after 60 min of BFA and BME treatment, and BFA was washed out to recover BIG1/2 function in MT-disrupted GBF1^M832L^ cells. Post-Golgi transport restarted after BFA washout, and GPI-AP gradually reached the plasma membrane (Fig. 4c and Supplementary Video 7). Notably, after BFA washout, GPI-AP and GalT co-localization increased slightly and then quickly decreased (Fig. 4e). The peak of GPI-AP accumulation in the Golgi was determined. The time constant of GPI-AP exit from the Golgi stacks, calculated from this peak, was only slightly longer than that in cells without BFA, although the difference was statistically significant (Fig. 4g). A similar restart of post-Golgi transport after accumulation on the Golgi was observed with TNFα after BFA washout in GBF1^M832L^ cells (Fig. 4j and Supplementary Video 7). These results indicate that BFA administration, followed by BFA washout in MT-disrupted GBF1^M832L^ cells, can be used to manipulate post-Golgi transport.

To determine whether manipulating post-Golgi transport using the GBF1^M832L^ mutation and BFA is a universal phenomenon, we examined the effects of BFA on post-Golgi transport in GBF1^M832L^ HEK293T cells. As with GBF1^M832L^ HeLa cells, BFA administration blocked post-Golgi transport of GPI-AP in GBF1^M832L^ HEK293T cells, which was then restarted by BFA washout (Supplementary Fig. 6g and Supplementary Video 8).

### Cargo accumulates near the *trans*-Golgi cisternae in MT-disrupted BFA-treated GBF1^M832L^ cells

**Fig. 5:**
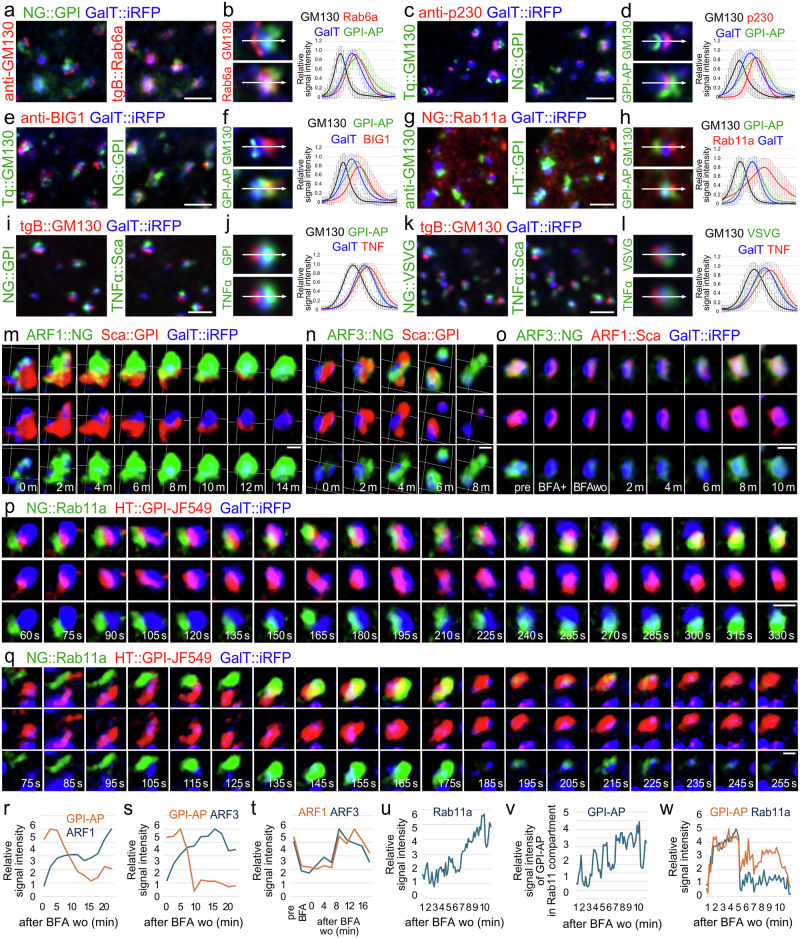
GPI-AP enters REs after BFA washout. **a**–**h** Localization of NeonGreen::GPI (green) 60 min post-BME administration in BFA-treated cells. GalT::iRFP713 is indicated in blue; (**a**) left red: *cis*-Golgi marker anti-GM130 antibody; right red: TGN marker tgB::Rab6a. **c** Red: TGN marker anti-p230 antibody; left green: *cis*-Golgi marker; Turquoise::GM130; right green: NeonGreen::GPI. **e** Red: TGN marker anti-BIG1 antibody; left green: *cis*-Golgi marker Turquoise::GM130; right green: NeonGreen::GPI. **g** Red: RE marker NeonGreen::Rab11a; left green: *cis*-Golgi marker anti-GM130 antibody; right green: Halo7-tag::GPI. **b**,** d**,** f**,** h** Normalized mean intensity profiles of 20 line scans across Golgi/RE units showing the relative distributions of Golgi, TGN, and RE markers with NeonGreen::GPI. Error bars represent the mean ± SD. The images on the left display typical Golgi/RE units. **i**–**l** TNFα::Scarlet, NeonGreen::GPI, and NG::VSVG localization 60 min after BME administration in BFA-treated cells. The cargo is indicated in green, the *cis*-Golgi marker tgB::GM130 is presented in red, and GalT::iRFP713 is indicated in blue. **j**,** l** Normalized means of the 20 line profiles of Golgi markers and cargo. Signal intensity was measured along the arrows (representing 1.5 μm). The images on the left display the typical Golgi/RE units. Error bars represent the mean ± SD. **m**,** n** Time-lapse images of ARF1::NeonGreen (m green) or ARF3::NG (n green) recruitment to the Golgi/RE unit, as well as Scarlet::GPI efflux (red) from the Golgi/RE unit after BFA washout. BFA was added 5 min after BME administration, and the cells were washed after 55 min. GalT::iRFP713 is indicated in blue. Five cells were observed for both ARF1 and ARF3. **o** Time-lapse images showing ARF1::Scarlet (red) and ARF3::NeonGreen (green) diffusion from the Golgi/RE unit after BFA administration, and their recruitment to the Golgi/RE unit after BFA washout. GalT::iRFP713 is indicated in blue. Five cells were observed. **p**, **q** High-speed time-lapse images showing GPI-AP transport from the TGN to the RE after BFA washout. BFA was administered 5 min after BME and washed out after 55 min. Images were captured using SCLIM, and the number in the lower-right corner indicates the elapsed time (s) after BFA washout. Halo7-tag::GPI-Janelia Fluor 549 is indicated in red, the RE marker NeonGreen::Rab11a in green, and GalT::iRFP713 in blue. Five cells were observed. **r**, **s** Relative signal intensities of GPI-AP (orange) and ARF1/3 (dark blue) after BFA washout in Fig. 5m, n. **t** Relative signal intensities of ARF1 (orange) and ARF3 (dark blue) pre BFA-treatment, during BFA-treatment, and after BFA-washout in Fig. 5o. **u**,** v** Relative signal intensities of Rab11 (**u**) and GPI-AP in Rab11 compartment (**v**) after BFA washout in Fig. 5p. **w** Relative signal intensities of GPI-AP in Rab11 compartment and Rab11 after BFA washout in Fig. 5q. Scale bars: 2 μm (**a**, **c**, **e**, **g**, **i**, **k**) and 1 μm (**m**–**q**).

Cargo localization was investigated in MT-disrupted BFA-treated GBF1^M832L^ cells. GPI-AP was localized between GalT and BIG1, and co-localized well with the TGN markers Rab6a and p230 (Fig. 5a–f). However, GPI-AP did not co-localize with the RE marker Rab11a, suggesting that its entry into REs depended on BIG1/2 activity (Fig. 5g, h). To investigate whether different types of cargo accumulate separately, we examined the two types simultaneously: two types of cargoes were localized close to each other and accumulated on the slightly *trans* side of GalT (Fig. 5i–l). Thus, cargoes accumulate in TGN in MT-disrupted BFA-treated GBF1^M832L^ cells.

ARF1/3 were recruited to Golgi stacks and co-localized with GPI-AP 2 min after BFA washout. ARF1/3 continued to increase on the membrane; however, the amount of GPI-AP gradually decreased (Fig. 5m–o, 5r–t and Supplementary Video 9).

SCLIM was used to investigate GPI-AP transport from TGN to RE^47,49,55^. In one Golgi stack, GPI-AP accumulated between GalT and Rab11a 60 s after BFA washout, and the GPI-AP signal gradually merged with that of Rab11a, becoming strongly co-localized (Fig. 5p, u, v and Supplementary Video 10). In another Golgi stack, the GPI-AP signal was separated from that of Rab11a for 125 s after BFA washout. They began to merge at 135 s and strongly co-localized shortly afterwards. At 185 s, both signals suddenly disappeared. Subsequently, the Rab11a signal increased gradually and then disappeared at 235 s after BFA washout, accompanied by a decrease in the GPI-AP signal (Fig. 5q, w and Supplementary Video 10). These results suggest that Rab11a-positive RE detached from the Golgi stack after receiving GPI-AP.

### GPI-AP is exported from the TGN via tubules and beads-on-a-string

**Fig. 6:**
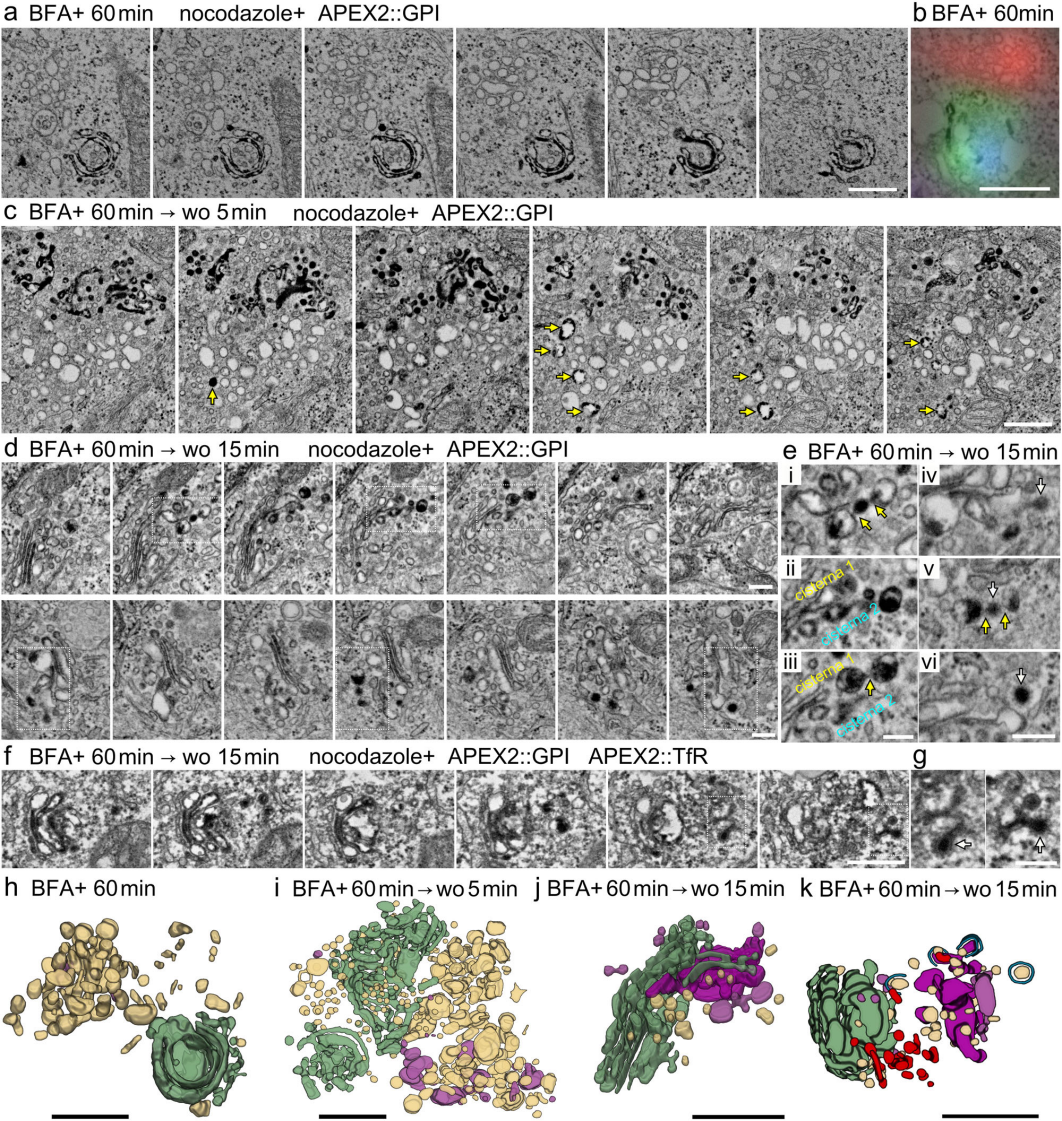
GPI-AP localization before and after BFA washout. **a**–**g** Scanning electron micrographs of serial sections of a Golgi stack and vesicle clusters at 50-nm intervals in GBF1^M832L^ cells. APEX2::GPI transport is initiated by the BME-RUSH system. The cytoplasmic APEX2 fused with TfR (APEX2::TfR) is also expressed in (**f**) and (**g**). **a** BFA was added 5 min after BME administration and fixed for 60 min. Twelve cells were observed in TEM, and five cells were observed in SEM. **b** Correlative light and electron micrographs. BFA was added 5 min after BME administration and fixed after 60 min. This cell line expresses Scarlet::Rab11a (red), GalT::iRFP713 (blue), and the RUSH cargo, EGFP::APEX2::GPI (green). Two cells were observed in the SEM. **c** BFA was added 5 min after BME administration, washed after 60 min, and fixed after 5 min. Yellow arrows indicate GPI-AP-containing vesicles. Fifteen cells were observed in TEM, and 12 cells were observed in SEM. **d**,** e** BFA was added 5 min after BME administration, washed after 60 min, and fixed after 15 min. Magnified images of the insets in (**d**) are shown in (**e**). Yellow arrows show vesicles connected by thin tubules (i, iii, and v). The “beads-on-a-string” were connected to the TGN (cisterns 1 and 2 in ii and iii). White arrows indicate vesicles or buds with a clathrin coat (iv–vi). Ten cells were observed in TEM, and four cells were observed in SEM. **f**,** g** BFA was added 5 min after BME administration, washed after 60 min, and fixed after 15 min. Magnified images of the insets in (**f**) are shown in (**g**). White arrows indicate TfR-positive clathrin-coated buds containing GPI-AP. Two cells were observed using TEM and two using SEM. **h**–**k** 3D images constructed from the serial sections. Golgi stacks are shown in green; APEX2::GPI-positive vesicles and tubules are shown in purple or pink; APEX2::GPI-negative vesicles are shown in yellow (**h**–**k**); APEX2::GPI-positive beads or tubule structures connected to the TGN are shown in purple (**j**). TfR::APEX-positive APEX2::GPI-negative membrane is shown in red; TfR::APEX-negative APEX2::GPI-positive membrane is shown in pink; and membrane positive for both markers is shown in purple (**k**). Scale bars: 500 nm (**a**–**c**), 200 nm (**d**, **e**), 500 nm (**f**), 200 nm (**g**), and 500 nm (**h**–**k**).

APEX2 was used to investigate cargo localization in MT-disrupted BFA-treated GBF1^M832L^ cells. The transport of RUSH cargo APEX2::GPI was induced by BME and stopped by the administration of BFA (Fig. 6 and Supplementary Video 11). APEX2::GPI signals accumulated within the Golgi stacks. Numerous vesicles, some of which were hemifused, accumulated near the stacks; however, APEX2 signals were not detected (Fig. 6a, h). This result is consistent with the observation that the TfR::APEX2 signal was localized in some of the vesicles accumulated near the Golgi stack (Supplementary Fig. 4d, e): GPI-AP is not localized in REs. Finally, we performed correlative light and electron microscopy (CLEM) to establish a direct link between the fluorescent microscopy data and the membrane structure observed in the electron microscopy images (Fig. 6b). The results indicated that GPI-AP is not localized on REs, which are part of the vesicle clusters.

Five minutes after BFA washout, GPI-AP was still primarily observed in the Golgi stacks; however, some was noted in the accumulated vesicles, which invaded the GPI-AP-negative vesicle clusters (Fig. 6c, i). In contrast, only a small amount of GPI-AP remained within the Golgi cisternae 15 min after BFA washout. Concentrated GPI-AP was located within the vesicles, which were often interconnected and resembled beads-on-a-string (Fig. 6d, e). Some of these beads were separated from the Golgi stacks (Fig. 6e-i, and v); however, others were connected to the TGN (Fig. 6e-ii, iii, cisterna 1). Notably, some vesicles or buds containing GPI-AP were coated with clathrin (Fig. 6e-iv–vi). Some clathrin-coated budding profiles without GPI-AP were also observed, even when directly connected to weakly GPI-AP-positive tubular structures (Supplementary Fig. 7a, b). The 3D-rendered model shows “beads-on-a-string” or “pearled” structures with GPI-AP connecting to TGN and the others separated from the TGN (Fig. 6j).

To investigate whether these GPI-AP-positive structures are REs, we performed double labeling of GPI-AP and REs using APEX2 in electron microscopy. APEX2::TfR, whose APEX2 is localized to the cytoplasmic side of REs, was co-expressed with APEX2::GPI, and observed 15 min after BFA washout. We found that the “pearled” structures containing GPI-AP were also positive for APEX2::TfR (Fig. 6f, g, k, Supplementary Fig. 7c–e and Supplementary Video 11).

### Adapter complex 1 is co-localized with GPI-AP at the TGN

**Fig. 7:**
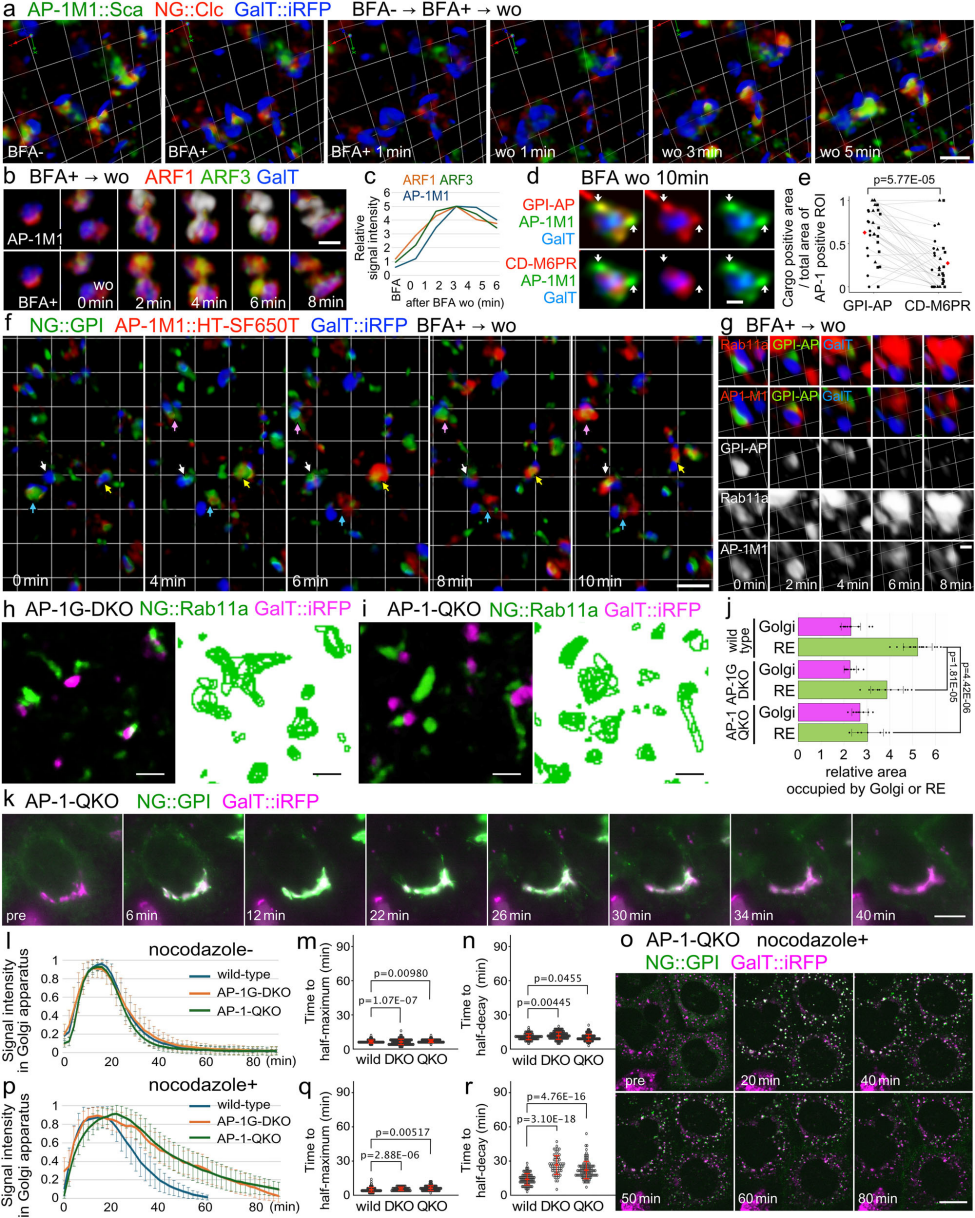
AP-1 is recruited to the membrane with GPI-AP after BFA washout. **a** Localization of NeonGreen::Clc (red), AP-1M1::Scarlet (green), and GalT::iRFP713 (blue) before and after BFA treatment and washout. Two cells were observed. **b** Localization of AP-1M1::Halo7-tag-Sara Flour 650T (white), ARF1::Scarlet (red), ARF3::NeonGreen (green), and GalT::iRFP713 (blue) before and after BFA washout. Two cells were observed. **c** Relative signal intensities of ARF1, ARF3 and AP-1-M1 during BFA-treatment and after BFA washout in (**b**). **d** Localization of cargo (red), AP-1M1::NeonGreen (green), and GalT::iRFP713 (blue) 10 min after BFA washout. Transport of two types of RUSH cargo (top: Halo7-tag::GPI-Sara Flour 650T; bottom: Scarlet::CD-M6PR) transport was induced by BME administration. BFA was added 5 min after BME administration, washed after 55 min, and fixed 10 min after BFA washout. **e** Quantification of the GPI-AP– or CD-M6PR–positive area within AP-1–positive ROIs near Golgi stacks. Thirty AP-1–positive ROIs from three cells were analyzed. The values for GPI-AP and CD-M6PR obtained from the same ROI are connected by lines. Red diamonds indicate the mean. Mean inclusive occupancy values were 0.628 for GPI-AP and 0.274 for CD-M6PR. Statistical analysis was performed using a one-sided two-sample *t* test assuming unequal variances (Welch’s *t* test). Thirty Golgi stacks were counted. **f**,** g** Localization of AP-1M1::Halo7-tag-Sara Flour 650T (red), NeonGreen::GPI (green), and GalT::iRFP713 (blue) before and after BFA washout. Arrows of the same color indicate the same Golgi stack across frames (**f**). Golgi stacks, indicated by blue arrows in (**f**), were magnified and presented at different angles in (**g**). Scarlet::Rab11a is shown in (**g**) in the indicated color. Three cells were observed. **h**,** i** Dynamics of REs in AP-1G-DKO (**h**) and AP-1-QKO (**i**) cells. The images on the left are frames from live imaging of GalT::iRFP713 (magenta) and NeonGreen::Rab11a (green). The right drawings are overlaid with traces of 11 RE positions at 15-second intervals. **j** The relative areas occupied by overlaid traces of 25 REs or Golgi positions at 5 s intervals are shown against the mean area of a single wild-type Golgi or RE trace. Error bars represent the mean ± SD. Statistical analyses were performed using a one-sided Welch’s *t* test with Holm adjustment for multiple comparisons. The following numbers of area were analyzed: wild-type: *n* = 18, AP-1G-DKO:* n* = 12, AP-1-QKO: *n* = 8. **k** Time-lapse images showing NeonGreen::GPI transport initiated by the BME-RUSH system in AP-1-QKO cells. NG::GPI is shown in green, and GalT::iRFP713 is shown in magenta. **l**–**n** Quantification of cargo transport kinetics: **l** Relative signal intensity of NeonGreen::GPI in the Golgi apparatus over time after BME administration. The blue, orange, and green lines represent wild-type, AP-1G-DKO, and AP-1-QKO cells, respectively. The Golgi apparatus is identified by a GalT::iRFP713 signal. The following numbers of cells were analyzed: wild-type: *n* = 101, AP-1G-DKO: *n* = 61, AP-1-QKO: *n* = 129. Error bars represent the mean ± SD. **m** Time constants for NeonGreen::GPI accumulation in the Golgi apparatus. The time constants for the wild-type, AP-1G-DKO, and AP-1-QKO cells were 6.5 ± 1.4, 7.8 ± 1.4, and 7.0 ± 1.3 min, respectively. Values are mean ± SD; error bars indicate SD. Statistical significance was determined using a two-tailed unpaired Student’s *t* test. **n** Time constants for NeonGreen::GPI exit from the Golgi apparatus. The time constants for the wild-type, AP-1G-DKO, and AP-1-QKO cells were 10.8 ± 2.6, 12.2 ± 3.1, and 10.0 ± 3.0 min, respectively. Values are mean ± SD; error bars indicate SD. Statistical significance was determined using a two-tailed unpaired Student’s *t* test. **o** Time-lapse images showing NeonGreen::GPI transport initiated by the BME-RUSH system in AP-1-QKO cells. BME treatment was initiated 4 h after incubation with nocodazole. NeonGreen::GPI is shown in green, and GalT::iRFP713 is shown in magenta. **p**–**r** Quantification of cargo transport kinetics: **p** Plots of the relative signal intensity of NeonGreen::GPI in the Golgi apparatus over time after BME administration. The blue, orange, and green lines represent wild-type, AP-1G-DKO, and AP-1-QKO cells, respectively. The Golgi apparatus is identified by a GalT::iRFP713 signal. The following numbers of cells were analyzed: wild-type: *n* = 100, AP-1G-DKO: *n* = 64, and AP-1-QKO: *n* = 104. Error bars represent the mean ± SD. **q** Time constants for NeonGreen::GPI accumulation in the Golgi stacks. The time constants for the wild-type, AP-1G-DKO, and AP-1-QKO cells were 4.8 ± 2.1, 5.6 ± 1.5, and 6.2 ± 2.1 min, respectively. Values are mean ± SD; error bars indicate SD. Statistical significance was determined using a two-tailed unpaired Student’s *t* test. **r** Time constants for NeonGreen::GPI exit from the Golgi stacks. The time constants for the wild-type, AP-1G-DKO, and AP-1-QKO cells were 13.8 ± 5.1, 26.4 ± 8.3, and 21.9 ± 7.7 min, respectively. Values are mean ± SD; error bars indicate SD. Statistical significance was determined using a two-tailed unpaired Student’s *t* test. Scale bars: 2 μm (**a**), 1 μm (**b**), 500 nm (**d**), 2 μm (**f**), 1 μm (**g**), 1 μm (**h**,** i**), and 10 μm (**k**, **o**).

As clathrin-coated vesicles or buds containing GPI-AP were observed by electron microscopy, the localization of the clathrin adapter complex AP-1 and clathrin was investigated. The μ subunit of AP-1 (AP-1M1) and the clathrin light chain (Clc) co-localized near GalT, and most disappeared rapidly after BFA administration. After the BFA washout, they were recruited to the vicinity of GalT and localized similarly as before BFA administration (Fig. 7a and Supplementary Video 12). Thus, BFA reversibly controlled AP-1 and clathrin localization. ARF1/3, AP-1, and Clc recruitments after BFA washout had similar timing (Fig. 7b, c).

Next, AP-1 localization was examined in fixed cells, in which GPI-AP and CD-M6PR exit from Golgi stacks were induced by BFA washout (Fig. 7d, e). Notably, AP-1 co-localized with GPI-AP but not with CD-M6PR, suggesting that it is specifically involved in GPI-AP transport. Live imaging showed that AP-1 was also recruited to the membrane where GPI-AP accumulated. The GPI-AP signal then gradually decreased, as did the AP-1 signal (Fig. 7f, g and Supplementary Video 13).

Finally, AP-1 and cargo localization were examined using RudLOV, a new light-induced cargo-releasing system^13,56^ that can control the amount of cargo released from the ER. After illumination-induced small amount of GPI-AP release from the ER, it entered the *cis* side of the Golgi stack at 0 min (Supplementary Fig. 8a and Supplementary Video 14). It then co-localized with GalT and Rab6a at 6 and 9 min. GPI-AP strongly co-localized with AP-1 and exited the Golgi stack 12 min after illumination. GPI-AP co-localization with AP-1 preceded that with Rab11a (Supplementary Fig. 8b and Supplementary Video 14). When TNFα was released from the ER, it entered from the *cis* side of the Golgi stack (0 min) and then co-localized with GalT and Rab6a (5–10 min) (Supplementary Fig. 8c and Supplementary Video 14). Then, TNFα accumulated in the Rab6a- or AP-1M1-negative membrane and exited the Golgi without colocalizing with Rab11a (Supplementary Fig. 8c, d and Supplementary Video 14). Thus, AP-1 specifically co-localizes with GPI-AP as it exits the Golgi stacks.

### RE is elongated and its motility is reduced in AP-1-deficient cells

Two types of AP-1-deficient cell lines with a GBF1^M832L^ allele were constructed (Supplementary Fig. 9). One was a double knockout (DKO) for AP-1G1 and G2, referred to as AP-1G-DKO cells. The other was a quadruple knockout (QKO) for AP-1G1, G2, M1, and M2, referred to as AP-1-QKO cells. AP-1-QKO cells completely lack AP-1, but AP-1G-DKO cells seem to exhibit residual AP-1 activity (Supplementary Fig. 9c, i).

Similar to wild-type cells, most Golgi stacks were accompanied by REs in MT-disrupted AP-1G-DKO and AP-1-QKO cells. However, the REs exhibited an elongated tubular morphology and reduced motility (Fig. 7h, i and Supplementary Video 15). The relative movement of the Golgi or REs showed that RE movement, but not Golgi movement, was reduced in AP-1G-DKO and AP-1-QKO cells (Fig. 7j). The number of RE attachments to and detachments from Golgi stacks was also significantly reduced in AP-1G-DKO and AP-1-QKO cells (Supplementary Fig. 9j).

### Post-Golgi transport of GPI-AP is delayed in AP-1-deficient cells

GPI-AP and VSVG transport was investigated in AP-1G-DKO and AP-1-QKO cells (Fig. 7k, Supplementary Fig. 10a, b, and Supplementary Video 16). In contrast to wild-type cells, numerous tubules extended from the Golgi apparatus in AP-1G-DKO and AP-1-QKO cells during cargo export. When simultaneously released from the ER, the same tubules contained GPI-AP and either VSVG or CD-M6PR (Supplementary Fig. 10c, d). Some tubules were Rab11a-positive (Supplementary Fig. 10e). This cargo transport using tubules in AP-1G-DKO and AP-1-QKO cells resembled that in BFA-treated GBF1^M832L^ cells (Fig. 3). Despite the presence of extended tubule-bearing cargo in AP-1G-DKO and AP-1-QKO cells, GPI-AP accumulation and exit from the Golgi stacks did not notably differ from that of the wild-type cells (Fig. 7l–n). In contrast, GPI-AP exits from the Golgi stacks in MT-disrupted AP-1G-DKO and AP-1-QKO cells were significantly delayed; however, GPI-AP accumulations in the Golgi stacks were not dramatically affected (Fig. 7o–r, Supplementary Fig. 10f, and Supplementary Video 16). Therefore, AP-1 is required for MT-independent post-Golgi transport of GPI-AP.

### RE formation on GPI-AP-accumulated membranes near Golgi stacks during post-Golgi transport in MT-disrupted AP-1-deficient cells

**Fig. 8:**
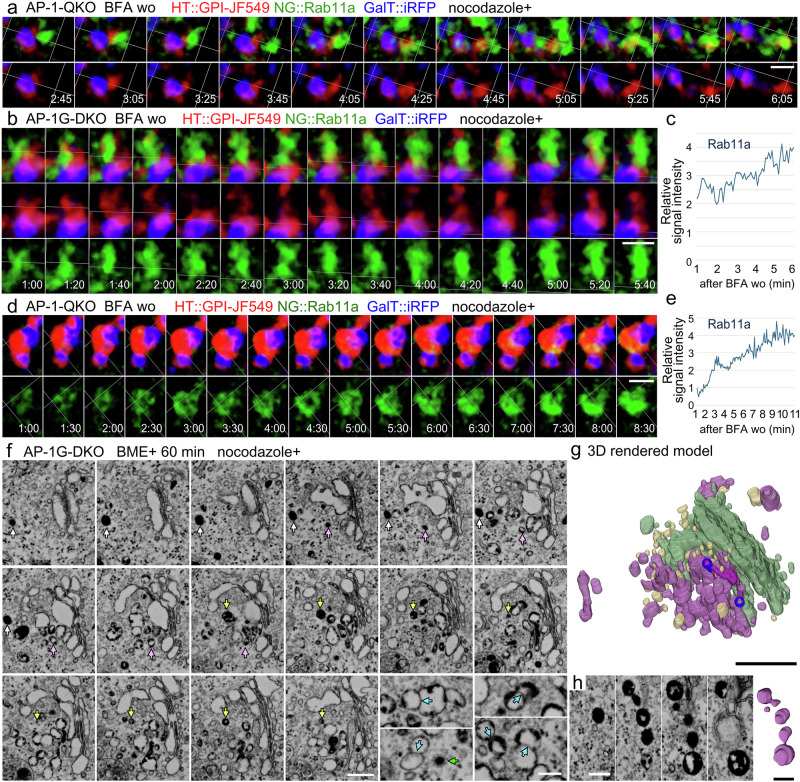
Cargo transport in AP-1G-DKO cells. **a**,** b** High-speed time-lapse images show GPI-AP transport from the TGN to REs after BFA washout in AP-1-QKO (**a**) and AP-1G-DKO cells (**b**). BFA was added 5 min after BME administration, and the cells were washed 55 min later. Images were taken with SCLIM, and the number in the lower right corner indicates the elapsed time (min:sec) after BFA washout. Halo7-tag::GPI-Janelia Fluor 549 is indicated in red, the RE marker NeonGreen::Rab11a in green, and GalT::iRFP713 in blue. Five AP-1G-DKO and four AP-1-QKO cells were observed. **c** Plot for the signal intensity of Rab11a during GPI-AP transport into the RE in (**b**). **d** High-speed time-lapse images show GPI-AP transport from the TGN to the RE after BFA washout in AP-1-QKO cells. BFA was added 5 min after BME administration, and the cells were washed after 55 min. Images were taken with SCLIM, and the number in the lower right corner indicates the elapsed time (min:sec) after BFA washout. Halo7-tag::GPI-Janelia Fluor 549 is indicated in red, NeonGreen::Rab11a in green, and GalT::iRFP713 in blue. **e** Plot of the signal intensity of Rab11a during GPI-AP transport into the RE in (**d**). **f** Scanning electron micrographs of serial sections at 50 nm intervals showing a Golgi stack and vesicle clusters in an AP-1G-DKO cell. APEX2::GPI transport was initiated using the BME-RUSH system and fixed after 60 min. White, pink, and yellow arrows indicate tubules containing APEX2::GPI. Blue arrows indicate hemifused vesicles, and green arrows indicate clathrin-coated vesicles containing GPI-AP. Four cells were observed using TEM, and four using SEM. **g** A 3D image reconstructed from serial sections as shown in (**f**). Golgi stacks are shown in green; APEX2::GPI-negative cisternae and vesicles are shown in yellow; APEX2::GPI-positive tubules are shown in pink; APEX2::GPI-positive tubules connected to the TGN are shown in purple. **h** Scanning electron micrographs of serial sections of possibly connected vesicles are shown on the left. A 3D-rendered render image was constructed from serial sections (right). Scale bars: 1 μm (**a**, **b**, **d**), 500 nm (**f**), 200 nm (**f** inset), 500 nm (**g**), and 200 nm (**h**).

SCLIM indicated that GPI-AP was incorporated into REs after BFA washout in AP-1G-DKO and AP-1-QKO cells (Fig. 8a, b, and Supplementary Video 17). RE growth on the GPI-AP-accumulating membrane was prominent after BFA washout (Fig. 8b, d), likely owing to reduced RE motility. A significant increase in Rab11a intensity was observed during GPI-AP incorporation after BFA washout (Fig. 8c, e). In summary, RE motility was reduced; however, GPI-AP transport to the RE was not severely affected in AP-1G-DKO or AP-1-QKO cells.

### GPI-AP-positive tubules and beads-on-a-string develop in AP-1G-DKO cells

The localization of APEX2::GPI and membrane structures 60 min after BME administration was investigated in MT-disrupted AP-1G-DKO and AP-1-QKO cells (Fig. 8f–h, Supplementary Fig. 11a, b and Supplementary Video 18). Numerous vesicles containing GPI-AP were observed on the *trans* side of Golgi stacks. Some vesicles were hemifused (Fig. 8f and Supplementary Fig. 11a, blue arrows), similar to those in BFA-treated cells (Fig. 2i). GPI-AP-positive tubules (Fig. 8f) and pearled structures (Fig. 8h and Supplementary Fig. 11a) were well developed. The 3D-rendered image shows the tubules running through the sections in the z-direction (Fig. 8g). Notably, some GPI-AP-positive vesicles or budding profiles were decorated with clathrin coats (Fig. 8f, g and Supplementary Fig. 11b).

The localization of APEX2::GPI cargo and membrane structures 15 min after BFA washout was also examined (Supplementary Fig. 11c, e, and Supplementary Video 18). Extensive APEX2::GPI-positive tubules were observed. The 3D-rendered model shows that numerous tubules containing GPI-AP were well-developed (Supplementary Fig. 11d). Some tubules were connected to the TGN (Supplementary Fig. 11f, g).

RE structures in AP-1-QKO cells were also investigated using TfR::APEX2 (Supplementary Fig. 12 and Supplementary Video 18). Many tubules positive for TfR::APEX2 were observed. Some tubules containing TfR::APEX2 were connected to the TGN, similar to GPI-AP positive tubules. Thus, these tubules are REs containing GPI-AP. These results suggest that AP-1 suppresses RE-tubule formation through tubule scission.

## Discussion

In MT-disrupted GBF1^M832L^ cells, BFA blocked post-Golgi transport without inducing Golgi absorption into the ER, and BFA washout restored it. This reversible system enabled detailed live imaging, electron microscopy, and biochemical assays, demonstrating its utility as a mammalian model for investigating post-Golgi transport. In MT-intact GBF1^M832L^ cells, BFA severely delayed, but did not block, post-Golgi transport, and numerous cargo-positive tubules extended from the Golgi ribbon, exporting cargo to the plasma membrane. This MT-dependent transport is independent of BFA-sensitive factors and conceals the effect of BFA on post-Golgi transport in MT-intact GBF1^M832L^ cells. At least some of the defects observed in BFA-treated GBF1^M832L^ cells are likely caused by BIG1/2 inhibition, as BIG1/2-DKD cells exhibit similar defects, albeit weaker, to those observed in BFA-treated GBF1^M832L^ cells. BIG1/2-DKD cells must have residual BIG1/2 activity.

Golgi stacks in mammalian cells naturally form MT-dependent Golgi ribbons, integrating MTs into post-Golgi transport. Therefore, MT-disrupted cells are artificial or non-physiological; however, manipulation of post-Golgi transport with BFA in MT-disrupted GBF1^M832L^ cells represents a useful experimental model. First, in wild-type HeLa cells, cargo reaches the plasma membrane without significant delay, even without MTs^57^. Second, Golgi stacks are distributed throughout the cytoplasm and do not form ribbons in numerous animals, such as *Drosophila melanogaster* and *Caenorhabditis elegans*, as well as in plants and fungi. Although the Golgi ribbon may have additional functionality, each independent Golgi stack is responsible for cargo sorting and export. This system enables the analysis of the fundamental processes of post-Golgi transport, which are prevalent in eukaryotic cells.

In GBF1^M832L^ cells, ARF1, ARF3, AP-1, and clathrin were recruited to GPI-AP-positive membranes shortly after BFA washout, and post-Golgi GPI-AP transport resumed. GPI-AP was gradually transferred to the pre-existing GA-REs; however, the signal intensity of Rab11a occasionally increased on the GPI-AP-positive membrane. This result suggests that this membrane with GPI-AP on the *trans* side of the Golgi stacks, presumably part of the TGN, matures into REs. Furthermore, the existence of GPI-AP-positive tubules and pearled structures connected to the TGN during export from the Golgi stacks indicates that these structures on the TGN could be the places of ongoing RE maturation (Supplementary Video 19).

Compared to wild-type cells, GPI-AP exit from Golgi stacks was significantly delayed in MT-disrupted AP-1-deficient cells. However, SCLIM observation after BFA washout in AP-1-deficient cells revealed normal GPI-AP transport from the TGN to the GA-RE, although Rab11a accumulation on the GPI-AP-positive membrane was more frequent than GPI-AP transfer into pre-existing REs. Therefore, GPI-AP transport from the TGN to REs was not blocked in AP-1-deficient cells. As GA-REs are closely associated with Golgi stacks, GPI-AP on GA-REs may be quantified as Golgi-localized in epifluorescence microscopy, which has a lower resolution than SCLIM. Together with the reduction in RE motility in AP-1-deficient cells, these results suggest that AP-1 is required for RE detachment from Golgi stacks for plasma membrane delivery, rather than for TGN-to-RE transport. Electron microscopy corroborated this finding: when GPI-AP is actively exported from Golgi stacks, AP-1-deficient cells develop GPI-AP-positive long tubules, some of which are connected to the TGN. These results suggest that AP-1 is involved in the scission of these tubules. These observations reinforce the hypothesis that these tubule and pearled structures are export carriers of GPI-AP from the TGN; in other words, GA-REs, and their detachment depends on AP-1 (Supplementary Video 19). Although the function of AP-1 and clathrin is likely vesicle formation, this process would result in tubule scission or RE detachment. These findings are consistent with those of previous reports, which show that the ARF1 compartment extends from the Golgi ribbon and matures into REs in an AP-1-dependent manner^58^; AP-1 localizes at the fission sites of the ARF1 compartment, and AP-1M1-KO induces cargo transport delays and ARF1 compartment elongation.

The GPI-AP-positive membrane on the TGN matured into Rab11a-positive compartments, presumably REs, after BFA washout. Golgi cisternal maturation extends to Golgi satellite organelles, ERGIC, or TGN in yeast^59,60^, and a similar expansion to endosomes has been reported in mammalian cells^61^. Retrograde transport of enzymes or factors is likely involved in RE maturation, as in Golgi cisternal maturation. Late Golgi or endosomal cisternal maturation is driven by AP-1–clathrin-mediated retrograde trafficking in yeast and mammalian cells^61,62^. However, the results of this study do not support the notion that AP-1 is the primary driver of TGN-RE maturation, because Rab11 deposition, if taken as an indicator of TGN-RE maturation, still occurs in AP-1-deficient cells. Endosome maturation from early to late endosomes is driven by Rab5-to-Rab7 conversion, rather than by retrograde transport^63^. Similarly, ARF1 compartments extending from the TGN convert into Rab11-positive compartments^58^. The authors proposed the hypothesis that ARF1-to-Rab11 conversion is essential for RE biogenesis. The results of this study are consistent with their hypothesis, as RE maturation of GPI-AP-positive membrane requires ARF1/3 function; it proceeds only after BFA-washout. These two mechanisms of TGN-RE maturation—AP-1–clathrin-mediated retrograde transport and ARF1-to-Rab11 conversion—are not mutually exclusive but may operate together. Therefore, a mixed model is proposed for the mechanism of RE-maturation. First, ARF1/3-to-Rab11 conversion in the TGN produces Rab11-positive membrane domains. Rab11 then recruits cytoplasmic factors, enabling membrane reception and exclusion via AP-1–clathrin-dependent retrograde transport. AP-1–clathrin vesicle formation also induces RE detachment from the TGN via tubule or pearled structure scission. In this model, ARF1/3 plays two distinct and crucial roles in RE maturation: ARF1/3-to-Rab11 conversion and AP-1 recruitment. Thus, BFA completely blocks post-Golgi GPI-AP transport, whereas AP-1 impairment only delays it.

Another surprising result of this study was the detection of numerous hemifused vesicles or cisternae in both BFA-treated GBF1^M832L^ and AP-1-deficient cells. Some hemifused vesicles in BFA-treated GBF1^M832L^ cells were Rab11-positive, and GPI-AP accumulated in hemifused vesicles in AP-1-deficient cells, suggesting these hemifused vesicles are related to REs and formed in a coatless condition. Cryo-electron tomography recently revealed the existence of hemifusomes, which are heterotypic hemifused vesicles associated with a proteolipid nanodroplet and are likely involved in ESCRT-independent multivesicular body formation^64^. Although the hemifused vesicles observed in this study are unlikely to be hemifusomes, the existence of hemifusomes suggests that the hemifused vesicles and cisternae are not artificial and could exist in wild-type cells, and they may persist in conditions lacking ARF or AP-1.

Various cargo molecules are transported from the TGN via tubular carriers in untreated mammalian cells. ARF1-coated tubular compartments transport TfR, LAMP1, TNFα, and VSVG, but not GPI-AP^58,65^. Similarly, GPI-AP is segregated from other cargoes, such as VSVG, TNFα, and CD-M6PR, at the TGN in MT-disrupted HeLa cells, and only GPI-AP is transported into GA-REs^11,13^. Thus, Golgi stacks/TGNs can sort cargo into specific carriers in MT-disrupted cells. BFA blocks all cargo transport from the TGN, whereas BFA washout resumes cargo sorting and transport into specific carriers in MT-disrupted GBF1^M832L^ cells. In contrast, cargo transport was delayed rather than blocked in BFA-treated MT-intact GBF1^M832L^ cells. Numerous cargo-positive tubules extend from the Golgi ribbon and transport the cargo to the plasma membrane. Notably, these tubules contained VSVG and CD-M6PR, together with GPI-AP, which transport them to the plasma membrane; however, under natural conditions, CD-M6PR is directly transported from the TGN to early endosomes^54,66^. Thus, BIG1/2-independent tubule carriers transport cargo without sorting. Similar to BFA-treated MT-intact GBF1^M832L^ cells, MT-intact AP-1-deficient cells developed tubules containing unsorted cargo such as VSVG, CD-M6PR, and GPI-AP. Thus, BIG1/2 functions, at least partly, through AP-1 to suppress premature tubule formation before sorting. This finding is consistent with the established model in which BIG1/2 activates ARFs, which recruit adapters and coat proteins to sort cargo.

BFA also inhibits the function of C-terminus binding protein 3/BFA adenosine diphosphate–ribosylated substrate (BARS) through ADP ribosylation^67,68^. BARS regulates the membrane-fission machinery that drives the formation of post-Golgi carriers containing VSVG, endocytic fluid-phase carriers^69,70^, macropinocytotic vesicles^71^, and COPI-coated vesicles^72^. BARS is also essential for Golgi partitioning during mitosis^73,74^. Consistently, vesicles in MT-disrupted BFA-treated GBF1^M832L^ cells were connected by constricted necks, indicating that scission defects occurred after BFA treatment. However, the IC_50_ of ADP-ribosylation of BARS by BFA is approximately 53–107 µM, much higher than the BFA concentration used in this study (10 µM)^75^. Therefore, the effects of BFA observed in this study were likely caused by BIG1/2 deficiency rather than BARS loss; however, future studies should identify targets for this phenotype.

## Methods

### Plasmid construction

The sequences and details of the construction of the plasmids used in this study are described in the Supplementary data file.

### Construction of BFA-resistant GBF1^M832L^ cells

BFA inhibits ARF protein activation by forming dead-end complexes with ARF-GDP and ARFGEF^76,77^. BFA-resistant ARFGEFs differ from BFA-sensitive ones by a few amino acid residues in their GDP/GTP exchange domains. Introducing a BFA-resistant residue into a BFA-sensitive ARFGEF converts it into a BFA-resistant ARFGEF and vice versa^78,79^. Methionine at position 832 (M832) in GBF1 is a key residue for BFA binding, and replacing it with leucine (L) converts GBF1 into a BFA-resistant form. The GBF1-specific inhibitor GCA binds to wild-type GBF1 but not to GBF1^M832L^ ^27^.

CRISPR/Cas9-mediated knock-in was performed to generate GBF1^M832L^ cells, which were screened for GCA resistance, and a single allelic GBF1^M832L^ cell line was cloned. A HeLa cell line stably expressing GalT::iRFP713 was transfected with the double-stranded DNA repair template (Supplementary data file) and ribonucleoprotein (RNP). To replace M832 on exon 18, two crRNAs were designed and synthesized (Alt-R CRISPR-Cas9 crRNA; Integrated DNA Technologies, Coralville, IA, USA) to target exons 17 and 19, respectively (Supplementary data file). The double-strand DNA repair template was designed to alter M832 to L. Introns were removed, and other codons were silently replaced for the region between the two CRISPR targets. Homology arms of 134 bp were added to both sides of the target sites. The double-strand DNA repair template was synthesized as a DNA block (gBlock, Integrated DNA Technologies) and amplified using PCR. Each crRNA (3 pmol) was annealed with an equimolar amount of tracrRNA (Integrated DNA Technologies) and then integrated with 6 pmol of Alt-R S.p. HiFi Cas9 Nuclease V3 (Integrated DNA Technologies) to form the RNP complex. The RNP complex and 250 ng of repair template were transfected using Lipofectamine RNAi MAX transfection reagent per the manufacturer’s instructions. The transfected cells were cultured in 5 µM GCA to select recombinants. The survivors were cloned through limited dilution to establish the GBF1^M832L^ cell line, clone B4.

### Construction of AP-1G-DKO and AP-1-QKO cells

Plasmids, primers, and CRISPR target sequences are described in the Supplementary data file. All CRISPR gRNAs were designed using CRISPOR (https://crispor.gi.ucsc.edu, 10.1093/nar/gky354) and cloned into pX-HFCas9^80^. For the constructs carrying tandem gRNAs, pX-HFCas9-pitch 2 was used as a PCR template to generate the gRNA-tRNA-Protospacer DNA fragment. To generate knockout cells, pX-HFCas9-based plasmids were transfected using JetOptimus (Polyplus-transfection, Illkirch-Graffenstaden, France), and transformants were selected by adding 2 µg/mL puromycin 1–4 d after transfection. On days 6–10 after transfection, surviving cells were diluted and seeded on 96-well plates to clone via limited dilution. Obtained clones were lysed using Proteinase K and analyzed by PCR using KOD ONE DNA polymerase (TOYOBO, Osaka, Japan), with the subject primer pairs. For selected clones, PCR fragments were sequenced to analyze KO alleles.

### Live imaging and quantification of cargo transport using the BME-RUSH system

GBF1^M832L^ and AP-1G-DKO cells, which stably express GalT::iRFP713, were transfected with a DNA plasmid encoding RUSH system bi-cistronic expression plasmids (Str::KDEL_SBP::NG::GPI, Str::KDEL_SBP::NG::VSVG, and Str::KDEL_SBP::TNFα::NG) using jetPRIME or JetOptimus transfection reagent per the manufacturer’s instructions (Polyplus-transfection). The medium was replaced with fresh medium containing 25 µM of biliverdin 6–10 h after transfection. The following day, the release of streptavidin-binding peptide (SBP)- and NeonGreen-fused cargoes into the secretory pathway was induced by replacing the medium with 10 µM BME^53^ and 100 μg/mL of cycloheximide (Cayman Chemical, Ann Arbor, MI, USA). Occasionally, a 4-hour treatment with 10 µM nocodazole (Cayman Chemical) was performed before cargo release. 10 nM BFA was administered together with BME or, in some cases, 5 min after BME addition.

Time-lapse epifluorescent micrographs were obtained using an IX83 microscope (Evident Scientific, Tokyo, Japan) or an APX-100 microscope (Evident Scientific), and time-lapse confocal micrographs were obtained using an FV3000 confocal microscope (Evident Scientific) before and every 2 min after BME administration. For confocal microscopy, z-sections with a 0.4 μm interval were acquired, and their projection images were used for visualization and quantification. The signal intensity of the cargoes was measured in proximity to the Golgi apparatus labeled by GalT::iRFP713 using Fiji software (version 2.16.0/1.54p)^81^. ROIs were defined as either the entire cell or the Golgi ribbon region in cargo-expressing cells, and cropped images were generated based on these ROIs. GalT puncta were segmented as follows. A Median-blurred image (radius = 10–40) was generated from the GalT channel and subtracted from the original image using Image Calculator to reduce diffuse background signals. The resulting image was further processed with a median blur filter (radius = 1) and subsequently thresholded using the Otsu method to generate a binary mask. ROIs were then redefined as the GalT mask, and cells in which the largest ROIs exceeded 20% of the total image area were excluded from the analysis. The mean intensity of cargo signals within the GalT ROIs was measured. The mean and SD at each time frame were calculated from the mean intensities measured in individual cells. Half-maximum time was defined as the last time point before the value exceeded the midpoint between the initial and peak value. The half-decay time was defined as the time elapsed from the peak value to the midpoint between the peak and the minimum value observed after the peak.

To investigate co-localization of different cargoes or Rab11a in tubules, GBF1^M832L^ and AP-1G-DKO cells were transfected with a DNA plasmid encoding NeonGreen::Rab11a and/or one or two DNA plasmids encoding RUSH system bi-cistronic expression plasmids (Str::KDEL_SBP::NG::GPI, Str::KDEL_SBP::NG::VSVG, Str::KDEL_SBP::Sca::VSVG, and Str::KDEL_SBP::Sca::TNFα). The remaining procedures were identical to those described above.

### Live imaging with high-quality confocal microscopy

GBF1^M832L^ and AP-1G-DKO cells, which stably express GalT::iRFP713, were transfected with DNA plasmids encoding the proteins of interest with fluorescent probes, organelle markers, the RUSH system bi-cistronic expression of a cargo and hook, and/or the RudLOV system bi-cistronic expression of a cargo and hook using JetOptimus transfection reagent according to the manufacturer’s instructions (Polyplus-transfection). The cells were cultured for 1 d in phenol red-free medium to reduce background fluorescence. They were then treated with 10 µM nocodazole for 4 h to disrupt the MTs (Cayman Chemical). For live imaging of RUSH cargoes, the release of SBP and NeonGreen-fused cargoes into the secretory pathway was induced by replacing the medium with 10 µM of BME^53^ and 100 μg/mL of cycloheximide (Cayman Chemical). For live imaging of RudLOV cargoes, the release of Zdk1 and fluorescent probe-fused cargoes into the secretory pathway was induced by 5 min of 445 nm illumination.

Time-lapse 3D stacks of confocal micrographs were obtained using an FV3000 microscope (Evident Scientific) or a STELLARIS5 microscope (Leica Microsystems, Wetzlar, Germany). The 3D stacks were projected using maximum intensity with Fiji software (version 2.16.0/1.54p). The obtained 3D voxel data were displayed as volume-rendered images using Volocity software (version 6.5.1; PerkinElmer, Waltham, MA, USA). The localization of cargo or other proteins of interest within the Golgi stacks was measured using line profiles across the Golgi stacks in Fiji software (version 2.16.0/1.54p) and processed with Plot2 Pro (Michael Wesemann, Berlin, Germany).　 The peak signal intensities of each of the three or four proteins (Golgi markers, ARFs, and cargoes) on 1.5 μm line were normalized to 100% within each Golgi stack, and the normalized profiles were then averaged from 15 independent Golgi stacks.

### Live imaging with SCLIM

GBF1^M832L^ cells stably expressing GalT::iRFP713, inoculated on glass-based dishes (Iwaki, Tokyo, Japan), were transfected with a DNA plasmid encoding NeonGreen::Rab11a and the RUSH system bi-cistronic expression plasmid Str::KDEL_SBP::Sca::GPI using JetOptimus transfection reagent per the manufacturer’s instructions (Polyplus-transfection). The cells were cultured for 1 day in phenol red-free medium to reduce background fluorescence. They were then treated with 10 µM nocodazole for 4 h to disrupt the MTs. SBP::Sca::GPI was released into the secretory pathway by replacing the medium with 10 µM of BME and 100 μg/mL of cycloheximide. Z-stack images were obtained using SCLIM^47,49,55^ and processed by deconvolution with Volocity software (version 6.5.1; PerkinElmer) using the theoretical point-spread function for spinning-disk confocal microscopy. The obtained 3D voxel data are displayed as volume-rendered images using Volocity software (version 6.5.1; PerkinElmer). Signal intensities of the indicated proteins were measured using Fiji software^81^ in BFA-treatment and BFA-washout experiments. z-stack images were converted into Sum-slices z-projections. Signals of the proteins of interest (POIs) were segmented from the corresponding channels using the Otsu thresholding method. ROIs were defined as POI masks within the Golgi stack, and the integrated density of each POI within its ROI was measured. In Fig. 5w, Rab11a signals were segmented to generate Rab11a ROIs, and the integrated densities of GPI-AP and Rab11a within the Rab11 ROIs were measured.

### Quantification of attachment and detachment frequencies between Golgi stacks and REs

SCLIM was used to quantify the frequencies of attachment and detachment events between Golgi stacks and REs in time-lapse images. Images were acquired at five-second intervals for 49 frames. Z-stack images were processed by deconvolution using Volocity software (version 6.5.1; PerkinElmer), employing the theoretical point-spread function for spinning-disk confocal microscopy. The z-stack images were then converted to maximum-intensity z-projections. The projected time-lapse images were analyzed frame by frame. REs that moved to within two pixels (120 nm) of the Golgi stacks were counted as attachment events. REs that subsequently moved away from this region were counted as detachment events. For each condition, ten Golgi stacks per cell were analyzed in three cells (*n* = 30).

### Quantification of the double-cargo colocalization

The spatial correlation between the two cargo signals was quantified using Fiji software^81^. For each cargo-containing region, the 2D cropped image showing the clearest cargo signals was manually selected from cropped images derived from the original time-lapse datasets. This image was then used for subsequent analysis. Analyses were performed at the three indicated time points (10, 30 and 50 min). In all cases, Pearson correlation analysis was performed between the two cargo channels (VSVG and GPI/TNFα). Binary masks of the two cargo signals were generated independently in Fiji. The Otsu method was used to automatically threshold each cargo channel and generate a binary mask representing the spatial distribution of each cargo signal in the selected 2D cropped image. A union ROI was defined for each cropped image as the set of pixels positive in either cargo mask. Thus, the union ROI included pixels belonging to the shared region, as well as pixels positive for only one cargo, while excluding pixels negative for both. Pearson correlation was then calculated using only the raw fluorescence intensities of the two cargo channels within the union ROI. Specifically, for each pixel within the union ROI, the raw intensity of one cargo and that of the other cargo were extracted and used to compute the Pearson correlation coefficient. This value is referred to in the figures as “Pearson’s colocalization coefficients (VSVG vs. GPI-AP/TNFα).”

### Quantification of the colocalization between AP-1 and cargoes

We quantified cargo occupancy within AP-1-positive structures using Fiji software^81^. All analyses were performed on manually cropped image regions selected from fixed cells. Cropped regions derived from the same original cell were grouped based on a cell identifier extracted from the file name. Ch1, Ch2, and Ch3 corresponded to AP-1, GPI, and CD-M6PR, respectively. The AP-1-positive puncta were segmented as follows: first, a Gaussian-blurred image (*σ* = 8 pixels) was generated from the AP-1 channel and subtracted from the original image to reduce diffuse background signals. The resulting image was then thresholded using the Otsu method to generate a binary mask. Finally, adjacent objects were separated using the watershed algorithm. The connected components in the AP-1 mask were treated as individual AP-1-positive ROIs.

GPI- and CD-M6PR-positive signals were segmented from their respective raw channels using the Shanbhag thresholding method. For each AP-1-positive ROI, all pixels belonging to the AP-1 component were examined against the GPI and CD-M6PR binary masks. Pixels were classified as GPI-only positive, CD-M6PR-only positive, or positive for both cargoes. GPI-only occupancy, CD-M6PR-only occupancy, and shared occupancy were then calculated as fractions of the AP-1-positive area based on these measurements. Inclusive GPI and CD-M6PR occupancies were defined as the fractions of AP-1-positive pixels overlapping with GPI- or CD-M6PR-positive signals, respectively. The mean intensity and integrated density of the GPI and CD-M6PR signals within each AP-1 ROI were also measured.

For the ROI-pooled analysis, ROIs lacking both GPI and CD-M6PR signals within the AP-1 group were excluded. After this filtering step, the number of ROIs from each cell was equalized through random sampling without replacement, with the same number of ROIs selected from each cell. Paired comparisons of GPI and CD-M6PR occupancies within each ROI were evaluated using a Wilcoxon signed-rank test.

### Immunostaining

GBF1^M832L^ cells expressing fluorescent proteins were fixed with 4% paraformaldehyde in 1× phosphate-buffered saline (PBS) for 5 min at room temperature. The cells were rinsed three times for 2 min each in 1 × PBS, then treated for 2 min in 1 × PBS containing 0.1% Triton X-100, followed by three 2-minute rinses in 1 × PBS. Blocking was performed by incubating the cells in 1 × PBS containing 5% fetal bovine serum for 30 min at room temperature. The cells were incubated for 1.5 h with primary antibodies and 5% bovine serum in 1 × PBS. They were then incubated for 1.5 h with fluorescent dye-labeled secondary antibodies in 1 × PBS containing 5% bovine serum, followed by three 2-minute rinses with 1 × PBS. The primary antisera used were rabbit anti-GM130 (1:250; #PM061, Medical & Biological Laboratories, Tokyo, Japan), mouse anti-BIG1 (1:200; #MABS1247, Merck, Darmstadt, Germany), and mouse anti-human TfR (1:500; #136800, Life Technology, Carlsbad, CA, USA). The secondary antibodies used were anti-mouse and anti-rabbit labeled with Alexa Fluor 488, 568, and 647 (1:300; Life Technologies). Immunostained cells were observed by confocal microscopy using a FV3000 microscope (Evident Scientific) or a STELLARIS5 microscope (Leica Microsystems).

### Electron microscopy imaging

Aclar films (Nisshin-EM, Tokyo, Japan) were cut into small sections, washed with acetone, placed in a µ-Slide 8-well cell culture chamber (ibidi GmbH, Gräfelfing, Germany), and then hydrated in cell culture medium for over 24 h. For CLEM-APEX analysis, the original cover glass of a glass-based dish (Iwaki) was carefully removed, and a grid-marked cover glass (Matsunami, Osaka, Japan) was reattached to the bottom of the dish using a UV-curable resin NOA 87 (Norland Products Inc., Jamesburg, USA). The Aclar films and grid-marked cover glasses were thinly coated with collagen to enhance cell adhesion. A collagen type I solution (Nippi, Tokyo, Japan) was mixed with an equivalent amount of 0.2 M HEPES in PBS on ice, then spread onto Aclar films or cover glasses and incubated for 5 min on ice. Aclar films or cover glasses were incubated at 37 °C for 1 h to promote gelation after the excess collagen solution was removed.

GBF1^M832L^ cells were seeded onto the films and grown overnight. For Fig. 2, the cells were incubated with 10 μM nocodazole for 4 h. For the BFA-untreated condition, the cells were fixed immediately after nocodazole treatment. For the BFA-treated condition, the cells were further incubated for 1 h with 10 μM nocodazole, 100 μg/mL cycloheximide, and 10 μM BFA before fixation.

For Fig. 6, GBF1^M832L^ cells were transfected either with a DNA plasmid encoding a RUSH system bi-cistronic expression plasmid (Str::KDEL_SBP::EGFP::APEX2::GPI) alone or co-transfected with DNA plasmids encoding TfR::APEX2::HT7 and Str::KDEL_SBP::EGFP::APEX2::GPI. For Fig. 6b, the cells were seeded onto grid-marked cover glass, whereas for the other experiments, the cells were seeded onto Aclar films and cultured overnight. In the experiments shown in Fig. 6b, d, the medium was replaced with fresh medium containing 25 µM biliverdin 6–10 h after seeding. EGFP::APEX2::GPI transport was induced by replacing the medium with 10 µM BME (made in the laboratory)^53^, 100 μg/mL cycloheximide and 10 µM nocodazole after 4 h of incubation with 10 μM nocodazole. After 5 min, BFA was added to a final concentration of 10 μM to inhibit the post-Golgi transport of EGFP::APEX2::GPI.

For Supplementary Fig. 4, GBF1^M832L^ cells were transfected with a DNA plasmid encoding TfR::APEX2::HT7, seeded onto Aclar films, and cultured overnight. For Supplementary Fig. 4a–c, the cells were incubated with 10 μM nocodazole for 4 h and then fixed. For Supplementary Fig. 4d, e, the cells were first incubated with 10 μM nocodazole for 2 h. They were subsequently incubated with 100 μg/mL cycloheximide and 10 μM nocodazole for an additional 2 h. Finally, they were incubated with 100 μg/mL cycloheximide, 10 μM nocodazole, and 10 μM BFA for 1 h before fixation.

For Fig. 8 and Supplementary Fig. 11, AP-1G-DKO and AP-1-QKO cells were transfected with a DNA plasmid encoding a RUSH system bi-cistronic expression plasmid (Str::KDEL_SBP::EGFP::APEX2::GPI) and seeded onto Aclar films, and cultured overnight. EGFP::APEX2::GPI transport was induced by replacing the medium with 10 μM nocodazole, 10 µM BME^53^ and 100 μg/mL cycloheximide after 4 h of incubation with 10 μM nocodazole. For Fig. 8 and Supplementary Fig. 11a and b, cells were incubated for 1 h and then fixed on ice for 1 h in EM fixation buffer. For Supplementary Fig. 11c and e, AP-1G-DKO and AP-1-QKO cells were incubated under the same conditions for 5 min, after which BFA was added to a final concentration of 10 μM. The cells were further incubated for 1 h, after which BFA was washed out, and the cells were incubated in BFA-free medium for 15 min before fixation. The cells were then fixed on ice for 1 h in EM fixation buffer.

For supplementary Fig. 12, AP-1-QKO cells were transfected with a DNA plasmid encoding TfR::APEX2::HT7, seeded onto Aclar films, and cultured overnight. The cells were incubated with 10 μM nocodazole for 4 h and then fixed immediately after nocodazole treatment.

Unless otherwise stated, cells were fixed on ice for 1 h in EM fixation buffer (0.1 M cacodylate buffer, pH 7.4, containing 2% glutaraldehyde and 2% paraformaldehyde; both from Electron Microscopy Sciences, Hatfield, PA, USA). The samples shown in Fig. 6b were fixed under different EM fixation conditions (0.1 M cacodylate buffer, pH 7.4, containing 1% glutaraldehyde and 3% paraformaldehyde) for CLEM-APEX analysis. After fixation, GBF1^M832L^ cells were counterstained with Hoechst 33342 (1:1000; FUJIFILM Wako Pure Chemical Corporation, Osaka, Japan) for 5 min in 0.1 M cacodylate buffer and then washed three times with the same buffer. The stained cells were observed using a STELLARIS5 microscope (Leica Microsystems).

After EM fixation, the cells were washed with 0.1 M cacodylate buffer, and 10 mM glycine was added. The cells were then incubated on ice for 20 min. Samples expressing APEX2 were subjected to 3,3′-diaminobenzidine (DAB) staining (Dojindo, Kumamoto, Japan) as previously described^50,51^ with modifications^82^. The samples were first incubated in a freshly diluted solution of 0.5 mg/ml (1.4 mM) DAB in chilled cacodylate buffer (DAB solution) without H₂O₂ (30%, FUJIFILM Wako Pure Chemical Corporation) for 20 min at room temperature to allow the DAB to penetrate the cells. The solution was then replaced with a fresh DAB solution containing 1 mM H₂O₂ and exchanged every 30 min until a dark APEX2-reaction product was observed. Once the DAB staining had developed, the DAB solution was removed, and the samples were washed at least seven times with 0.1 M cacodylate buffer.

Post-fixation was performed using 2% (w/v) osmium tetroxide (Electron Microscopy Sciences) for 30–60 min in chilled cacodylate buffer on ice. The films and cover glasses were rinsed with chilled distilled water more than seven times, placed overnight in double-distilled water containing chilled 2% (w/v) uranyl acetate, dehydrated, and then penetrated with EPON-812 resin (Quetol-812, 45.1%, dodecenylsuccinic anhydride, 13.9%, methyl nadic anhydride, 38.3%, DMP-30, 2.3%, Nisshin-EM). The grid-marked cover-glass-based dishes were processed through the dehydration step under the same conditions as those used for confocal microscopy. During dehydration, the grid-marked cover glasses detached easily with a slight mechanical force.

Aclar films and grid-marked cover glass with cells in EPON-812 were polymerized into a thin EPON-812 sheet at 100 °C for 20 h. The attached Aclar film was removed from the Aclar film samples. Polymerized EPON-812 sheet-containing cells were cut into squares, which were then placed on the bottom of a pyramidal mold with fresh EPON-812 resin and re-polymerized at 100 °C for 20 h. For the cover glass-mounted samples, the EPON layer on the side opposite the cultured cells was trimmed with an ultrasonic cutter (ZO-41 II; Echotech Corporation, Aichi, Japan) to expose the underlying glass surface. The sample was then immersed in hydrofluoric acid (FUJIFILM Wako Pure Chemical Corporation) to remove the cover glass, yielding a polymerized EPON-812 sheet containing embedded cells. Regions of interest corresponding to the grid marks were cut from the sheet, placed at the bottom of a pyramidal mold with fresh EPON-812, and re-polymerized at 100 °C for 20 h. For serial-section scanning electron microscopy (SEM), ultrathin sections (50 nm thickness) were cut using a 45° diamond knife (Diatome, Nidau, Switzerland) or a SYM2035WT Ultra knife (Syntek, Tokyo, Japan) mounted on an ultramicrotome (EM UC7; Leica Microsystems). The sections were then placed on hydrophilized silicon wafers. Serial-section SEM was performed using a high-resolution field-emission scanning electron microscope and a backscattered electron detector. Specific staining and imaging conditions are shown in each figure.

The sections were stained with 0.4% uranyl acetate for 10 min and lead stain solution (Sigma-Aldrich, St. Louis, MO, USA) for 2 min and then coated with osmium tetroxide using an osmium coater (HPC-1SW, Vacuum Device Inc., Ibaraki, Japan) (Fig. 2). Serial sections were observed using a Regulus8240 instrument (Hitachi High-Tech, Tokyo, Japan) equipped with an auto-capture for array tomography and a low-angle BSE detector at an accelerating voltage of 2 kV (Fig. 2). The sections were stained with a lead stain solution (Sigma-Aldrich) for 3 min at room temperature (Figs. 6, 8 and Supplementary Figs. 4, 12). Transmission electron microscopy images were acquired using a JEM-1400plus (JEOL, Tokyo, Japan). Serial sections were observed using a HeliosG4 UC (Thermo Fisher Scientific, Waltham, MA, USA) equipped with Maps 3.19, a circular backscattered electron detector (CBS). The observations were conducted at an accelerating voltage of 3 kV, a beam current of 0.4 nA, and a working distance of 3.5 mm using CBS. Serial-section images were acquired as a set of partially overlapping tiles. Tile stitching was performed using vEMstitch, an automated image-stitching algorithm^83^, to generate seamless images for each section. After stitching, the images were manually aligned using the TrakEM2 plugin for Fiji software (version 2.16.0/1.54p)^81^. 3D segmentation and reconstruction were performed manually using 3D Slicer^84^. CLEM image registration was performed manually based on the relative positions of the nuclei and the Golgi apparatus.

### Immunoblotting

The cells were washed with ice-cold PBS and lysed using RIPA buffer (FUJIFILM Wako Pure Chemical Corporation) supplemented with benzonase nuclease (Sigma-Aldrich) and a protease inhibitor cocktail set V (FUJIFILM Wako Pure Chemical Corporation). SDS-containing sample buffer (4 ×) was added to the lysates, and the samples were incubated at 95 °C for 5 min. The samples were separated using SDS-PAGE on a 10% acrylamide gel and blotted onto polyvinylidene fluoride (PVDF) membranes (Millipore Sigma, Burlington, MA, USA). The primary antibodies and antisera used were mouse anti-BIG1 (1:1000; #MABS1247, Merck, Darmstadt, Germany), mouse anti-BIG2 (1:1000; #MABS1246, Merck), mouse anti-GBF1 (1:2000; #612116, BD Transduction, Franklin Lakes, USA), mouse anti-AP-1G1 (1:3000; SAB4200858; Sigma-Aldrich), rabbit anti-AP-1G2 (1:300; HPA004106; Sigma-Aldrich), rabbit anti-AP-1M1 (1:1000; 12112-1-AP; Proteintech Group, Inc., Rosemont, IL, USA), and rabbit anti-AP-1M2 antibodies (1:1000; 10618-1-AP; Proteintech Group, Inc.). This step was followed by incubation with horseradish peroxidase (HRP)-conjugated anti-mouse or anti-rabbit IgG antibodies (1:20,000; Jackson ImmunoResearch, West Grove, PA, USA). Signals were visualized using Clarity Western ECL Substrate (Bio-Rad Laboratories, Hercules, CA, USA) and imaged using a ChemiDoc XRS+ (Bio-Rad Laboratories). The same PVDF membrane was stripped with WB Stripping Solution (Nacalai Tesque, Kyoto, Japan) and reprobed with rat anti-α-tubulin (1:20,000; #MCA78G, Bio-Rad Laboratories), followed by HRP-conjugated anti-rat IgG (1:20,000; Jackson ImmunoResearch) and signal visualization.

### Co-immunoprecipitation of AP-1M1::Clover

HeLa cells plated in 60 mm dishes were transfected with a DNA plasmid encoding AP-1M1::Clover using the JetOptimus transfection reagent (Polyplus-transfection) according to the manufacturer’s instructions. The medium was replaced with fresh medium 8 h after transfection. The following day, the cells were washed with cold PBS (Nacalai Tesque) and lysed with 1 mL of RIPA buffer (FUJIFILM Wako Pure Chemical Corporation) supplemented with benzonase nuclease (Sigma-Aldrich) and protease inhibitor cocktail set V (FUJIFILM Wako Pure Chemical Corporation). The lysates were mixed with 5 µL of GFP-Trap magnetic agarose beads (ChromoTek, Planegg-Martinsried, Germany), which had been pre-equilibrated with RIPA buffer. Samples were rotated at 4 °C for 2 h, and the beads were washed three times with RIPA buffer. The beads were washed twice with 25 mM triethylammonium bicarbonate (TEAB; Thermo Fisher Scientific) to remove detergents and transferred to fresh low-binding tubes. To elute the proteins from the beads, 50 µL of 25 mM TEAB containing 1% sodium deoxycholate (Nacalai Tesque) was added, and the samples were boiled at 95 °C for 5 min. The eluates were collected in fresh low-binding tubes for mass spectrometry sample preparation.

### Mass spectrometry sample preparation

The samples were reduced with a 10 mM TCEP solution (Thermo Fisher Scientific) in 25 mM TEAB for 30 min at 37 °C, and then alkylated with 5 mM iodoacetamide (FUJIFILM Wako Pure Chemical Corporation) for 30 min at room temperature in the dark. The samples were digested overnight at 37 °C with 250 ng of Trypsin Platinum (Promega Corporation, Madison, WI, USA) prepared in 25 mM TEAB. The resulting peptides were labeled with TMTpro 18plex reagents (ZE390055; Thermo Fisher Scientific) overnight at 37 °C, and the labeling reaction was quenched with 2 M methylamine (Thermo Fisher Scientific) for 1 h at 37 °C. Sodium deoxycholate was removed using the phase-transfer surfactant method with formic acid (FUJIFILM Wako Pure Chemical Corporation) and ethyl acetate (FUJIFILM Wako Pure Chemical Corporation). The samples were desalted using SDB-StageTips and analyzed using liquid chromatography–mass spectrometry after phase separation and removal of the organic layer.

### Reporting summary

Further information on research design is available in the Nature Portfolio Reporting Summary linked to this article.

## Supplementary information

Supplementary Information

Description of Additional Supplementary Files

Supp Data 1

Video 1

Video 2

Video 3

Video 4

Video 5

Video 6

Video 7

Video 8

Video 9

Video 10

Video 11

Video 12

Video 13

Video 14

Video 15

Video 16

Video 17

Video 18

Video 19

Reporting Summary

Transparent Peer Review file

## Source data

Source data

## Data Availability

The original microscopy images are available on Figshare with the dataset identified at 10.6084/m9.figshare.32684082. The raw mass spectrometry proteomic data were deposited in the jPOST repository^85^ with the dataset identified as JPST004467. Source data are provided in this paper.
