# Supplementary material for: Reversible control of post-Golgi transport by brefeldin A reveals recycling endosome maturation during glycosylphosphatidylinositol-anchored protein transport
Source: Nat Commun. 2026 Jul 27;17:7262. doi: 10.1038/s41467-026-75784-1 (PMC13408501; doi:10.1038/s41467-026-75784-1)
Supplement: Supplementary file 1 — Supplementary Information [file 41467_2026_75784_MOESM1_ESM.pdf]

Supplementary Information

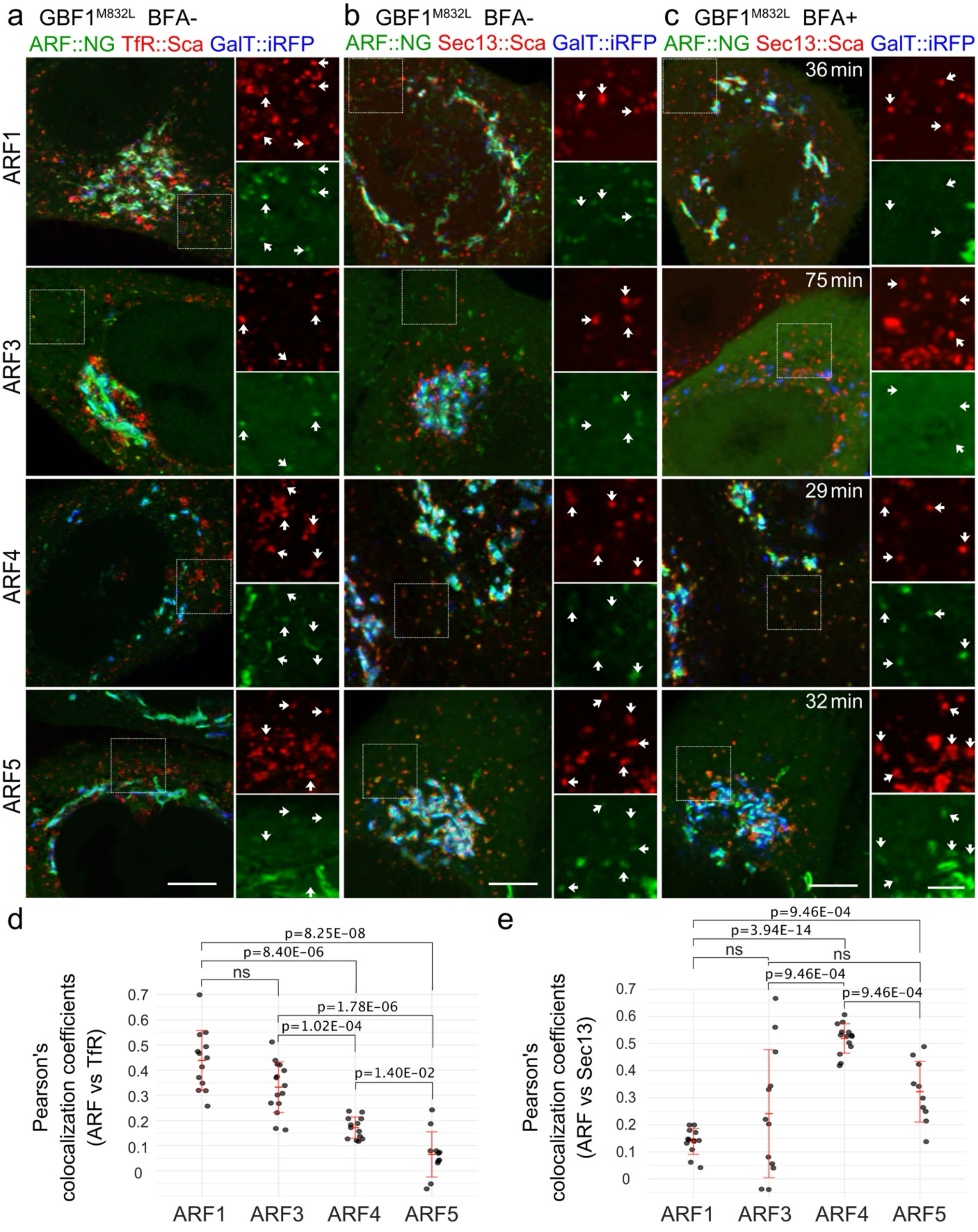

Supplementary Figure 1. ARF1 and ARF3 are sensitive to brefeldin A (BFA) in GBF1^{M832L} cells

a–c Localization of ARF1::NG, ARF3::NG, ARF4::NG, and ARF5::NG (green) before (a, b) and after (c) BFA administration in GBF1^{M832L} cells. The recycling endosome (RE) marker (TfR::Sca) is shown in red in (a), and the endoplasmic reticulum (ER) exit site (ERES) marker (Sec13::Sca) is shown in red in (b, c). The *trans*-Golgi marker (GalT::iRFP) is indicated in blue. The time indicated in the upper-right corner represents the incubation time with BFA. The right panels show magnified single-color images of the insets of the left images. Arrows show RE (a) or ERES (b, c).

d, e Plots showing the colocalization of ARFs with TfR (d) or Sec13 (e). Error bars represent the mean ± SD (ARF1-TfR: 0.439 ± 0.119, ARF3-TfR: 0.332 ± 0.100, ARF4-TfR: 0.172 ± 0.042, ARF5-TfR: 0.065 ± 0.090, ARF1-Sec13: 0.138 ± 0.048, ARF3-Sec13: 0.241 ± 0.236, ARF4-Sec13: 0.519 ± 0.054, ARF5-Sec13: 0.317 ± 0.108). Results of the Games-Howell post-hoc test for each combination of samples are indicated. The following numbers of cells were analyzed: ARF1-TfR: n=13, ARF3-TfR: n=15, ARF4-TfR: n=13, ARF5-TfR: n=11, ARF1-Sec13: n=13, ARF3-Sec13: n=12, ARF4-Sec13: n=14, ARF5-Sec13: n=11.

Scale bars: 5 μm (a–c) and 2 μm (insets of a–c)

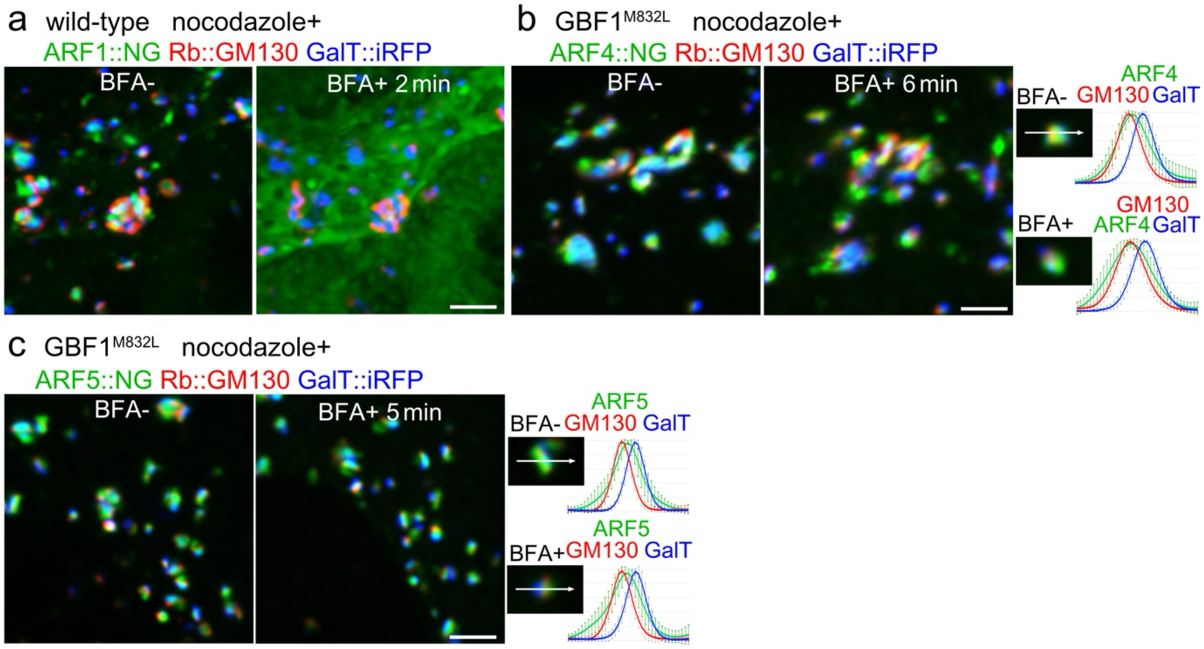

Supplementary Figure 2. ARF4 and ARF5 are insensitive to BFA in GBF1^{M832L} cells

a ARF1::NG localization before (left) and 2 min after (right) BFA administration in nocodazole-treated wild-type cells. The *cis*-Golgi marker (Rb::GM130) is shown in red, GalT::iRFP is shown in blue, and ARF1::NG is shown in green.

b, c ARF4::NG (b) and ARF5::NG (c) localization before (left) and 6 or 5 min after BFA administration (right) in nocodazole-treated GBF1^{M832L} cells. Rb::GM130 is shown in red and GalT::iRFP is shown in blue. ARF4::NG (b) and ARF5::NG (c) are shown in green. The plots show the normalized mean values for 15 ARF and Golgi markers line profiles across the Golgi stack. The image on the left shows a typical Golgi stack.

Scale bars: 2 µm (a–c)

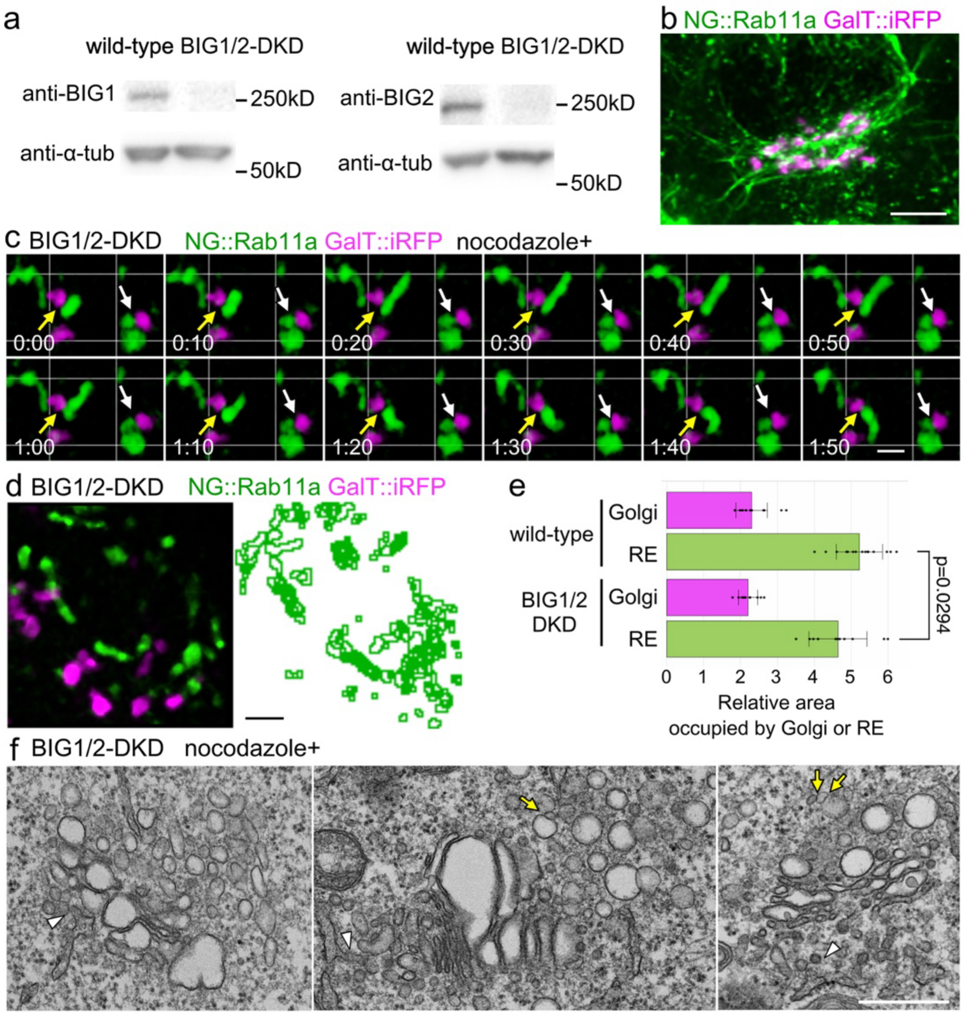

a
wild-type BIG1/2-DKD
anti-BIG1
anti-α-tub
250kD
50kD
wild-type BIG1/2-DKD
anti-BIG2
anti-α-tub
250kD
50kD
b NG::Rab11a GalT::iRFP
c BIG1/2-DKD NG::Rab11a GalT::iRFP nocodazole+
0:00
0:10
0:20
0:30
0:40
0:50
1:00
1:10
1:20
1:30
1:40
1:50
d BIG1/2-DKD NG::Rab11a GalT::iRFP
e
wild-type
Golgi
RE
BIG1/2 DKD
Golgi
RE
p=0.0294
0 1 2 3 4 5 6
Relative area occupied by Golgi or RE
f BIG1/2-DKD nocodazole+

Supplementary Figure 3. RE motility is reduced by BIG1 and BIG2 knockdown

Cells 72 hours after BIG1/2 siRNA transfection were used as BIG1/2 double-knockdown (DKD) cells.

a Immunoblotting of extracts prepared from wild-type and BIG1/2-DKD cells using anti-BIG1, anti-BIG2, and anti-α-tubulin antibodies. Six cells were observed.

b Localization of REs and the Golgi ribbon in BIG1/2-DKD cells. NG::Rab11a is shown in green, and GalT::iRFP is shown in magenta.

c Time-lapse images showing the dynamics of REs (green) and Golgi stacks (magenta) in BIG1/2-DKD cells. Arrows indicate REs stably associated with Golgi stacks. Twenty cells were observed.

d Dynamics of REs in BIG1/2-DKD cells. The images on the left are frames from live imaging of GalT::iRFP (magenta) and NG::Rab11a (green). The drawings on the right are overlaid with traces of 11 RE positions at 15-second intervals.

e The relative areas occupied by overlaid traces of 25 REs or Golgi positions at 5-second intervals are shown against the mean area of a single wild-type Golgi or RE trace. Wild-type data from Figure 2g are shown for comparison. Error bars represent the mean ± SD. Statistical significance was assessed using a one-sided two-sample t-test assuming unequal variances (Welch's t-test). The following numbers of area were analyzed: wild-type: n=18, BIG1/2-DKD: n=11.

f Transmission electron micrographs of Golgi stacks in BIG1/2-DKD cells. Arrowheads indicate the ERES. Yellow arrows indicate hemi-fused vesicles. Sixteen cells were observed in TEM.

Scale bars: 5 µm (b) 1 µm (c, d), and 500 nm (f)

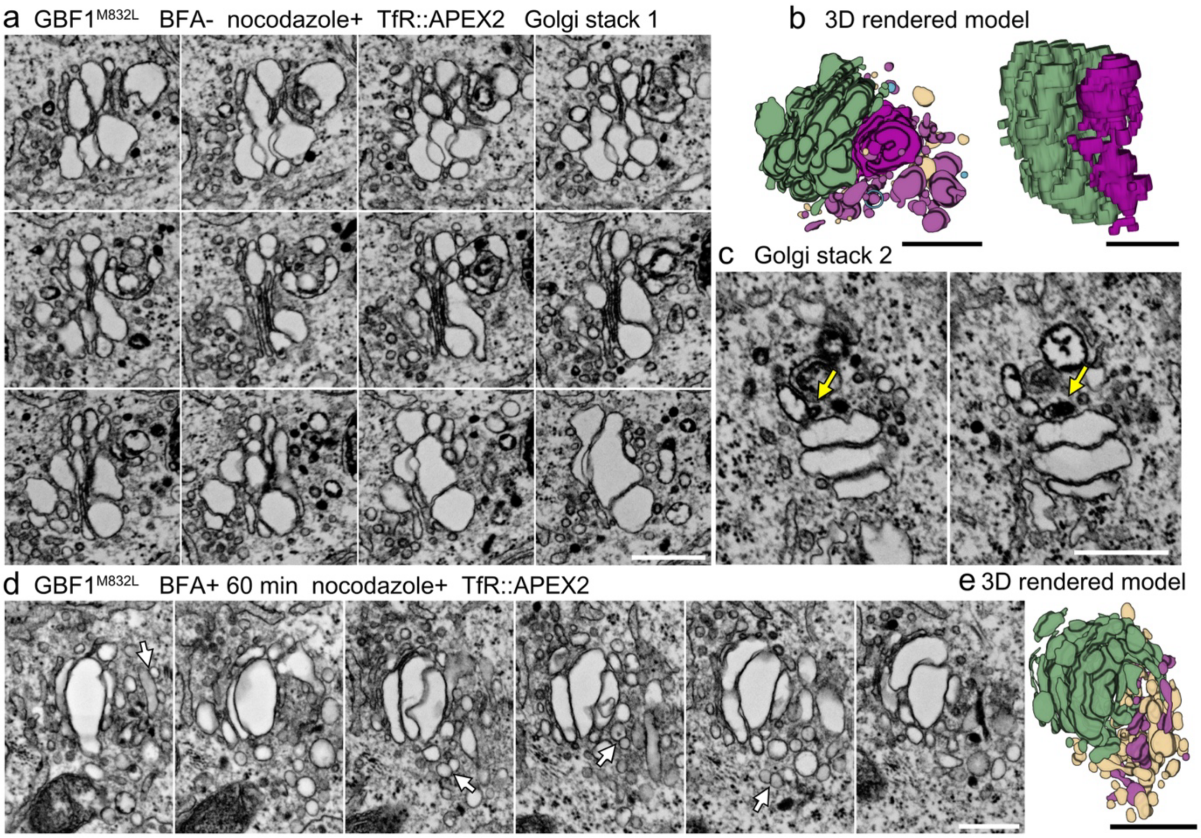

Supplementary Figure 4. Localization of recycling endosomes in BFA-treated and untreated GBF1^{M832L} cells

Scanning electron micrographs of serial sections at 50-nm intervals of Golgi stacks, tubules, and vesicles in BFA-untreated (a, c) and BFA-treated (d) GBF1^{M832L} cells expressing the RE marker TfR::APEX2 (APEX2 localized in the lumen). Yellow arrows indicate TfR::APEX2-positive cisternae. 3D images reconstructed from serial sections are shown in (b) and (e). Cells were pretreated with nocodazole for 4 h. Golgi stacks are shown in green, whereas TfR-positive and TfR-negative membranes are shown in pink and yellow, respectively (b, left and e). Clathrin coats are shown in blue. TfR-positive membranes connected to Golgi stacks are shown in purple (b, right). The following numbers of cells were analyzed: BFA-: n=4 (TEM) and 4 (SEM), BFA+ 60 min: n=7 (SEM).

Scale bars: 500 nm (a–e)

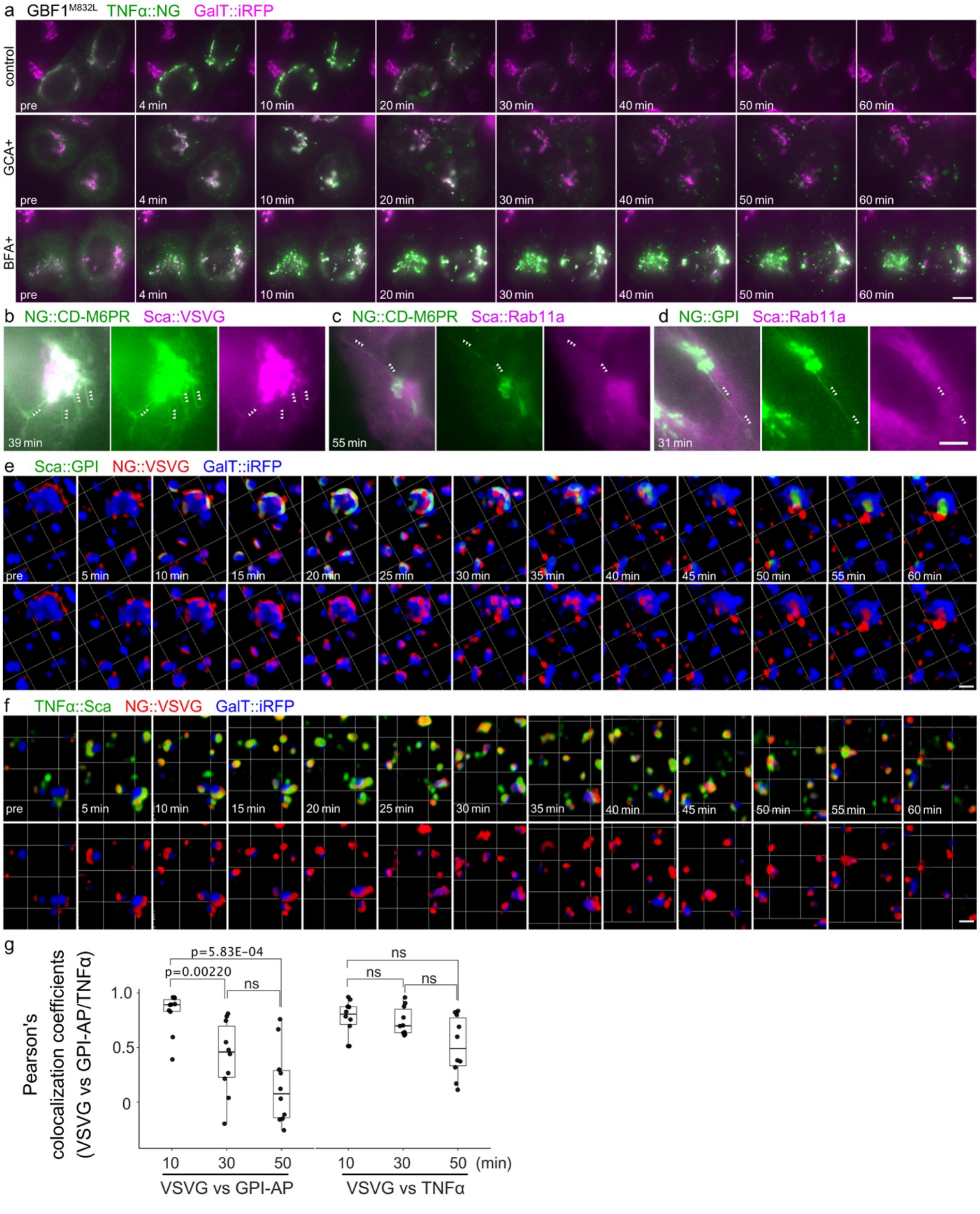

a
GBF1^{M832L} TNFα::NG GalT::iRFP
control
GCA+
BFA+
pre
4 min
10 min
20 min
30 min
40 min
50 min
60 min
b
NG::CD-M6PR Sca::VSVG
39 min
c
NG::CD-M6PR Sca::Rab11a
55 min
d
NG::GPI Sca::Rab11a
31 min
e
Sca::GPI NG::VSVG GalT::iRFP
pre
5 min
10 min
15 min
20 min
25 min
30 min
35 min
40 min
45 min
50 min
55 min
60 min
f
TNFα::Sca NG::VSVG GalT::iRFP
g
Pearson's colocalization coefficients (VSVG vs GPI-AP/TNFα)
p=5.83E-04
p=0.00220
ns
1.0
0.5
0
10
30
50
(min)
VSVG vs GPI-AP
VSVG vs TNFα

Supplementary Figure 5. Vesicular stomatitis virus glycoprotein (VSVG) and tumor necrosis factor-alpha (TNFα) but not glycosylphosphatidylinositol-anchored protein (GPI-AP) strongly co-localize during transport

a Frames from a time-lapse movie showing TNFα::NG transport initiated by the biotin methyl ester (BME)-RUSH system in untreated cells (top), Golgicide A (GCA) (middle), or BFA-treated (bottom). TNFα::NG is shown in green, and GalT::iRFP is shown in magenta.

b–d Co-localization studies in BFA-treated cells: **b** NG::CD-M6PR (green) and Sca::VSVG (magenta) localization 39 min after BME administration; **c** NG::CD-M6PR (green) and Sca::Rab11a (magenta) localization 55 min after BME administration; **d** NG::GPI (green) and Sca::Rab11a (magenta) localization 31 min after BME administration. Arrowheads indicate the co-localization of cargo pairs (b) or cargo with Rab11a (c, d). The following numbers of cells were analyzed: NG::CD-M6PR vs Sca::VSVG: n=2, NG::CD-M6PR vs Sca::Rab11a: n=6, NG::GPI vs Sca::Rab11a: n=2.

e, f Frames of the time-lapse movie showing NG::VSVG (red) and Sca::GPI (green) transport (e) or NG::VSVG (red) and TNFα::Sca (green) transport (f) initiated by the BME-RUSH system. GalT::iRFP is indicated in blue. The following numbers of cells were analyzed: NG::VSVG vs Sca::GPI: n=3, NG::VSVG vs TNFα::Sca: n=6.

g Plot showing the colocalization between the two cargoes during transport shown in (e) and (f). The left panel shows VSVG versus GPI-AP, and the right panel shows VSVG versus TNFα. Statistical analyses were performed using a two-sided Wilcoxon rank-sum test followed by Benjamini–Hochberg correction. Colocalizations of double cargoes were measured in ten Golgi stacks.

Scale bars: 10 μm (a), 5 μm (b–d), and 1 μm (e, f)

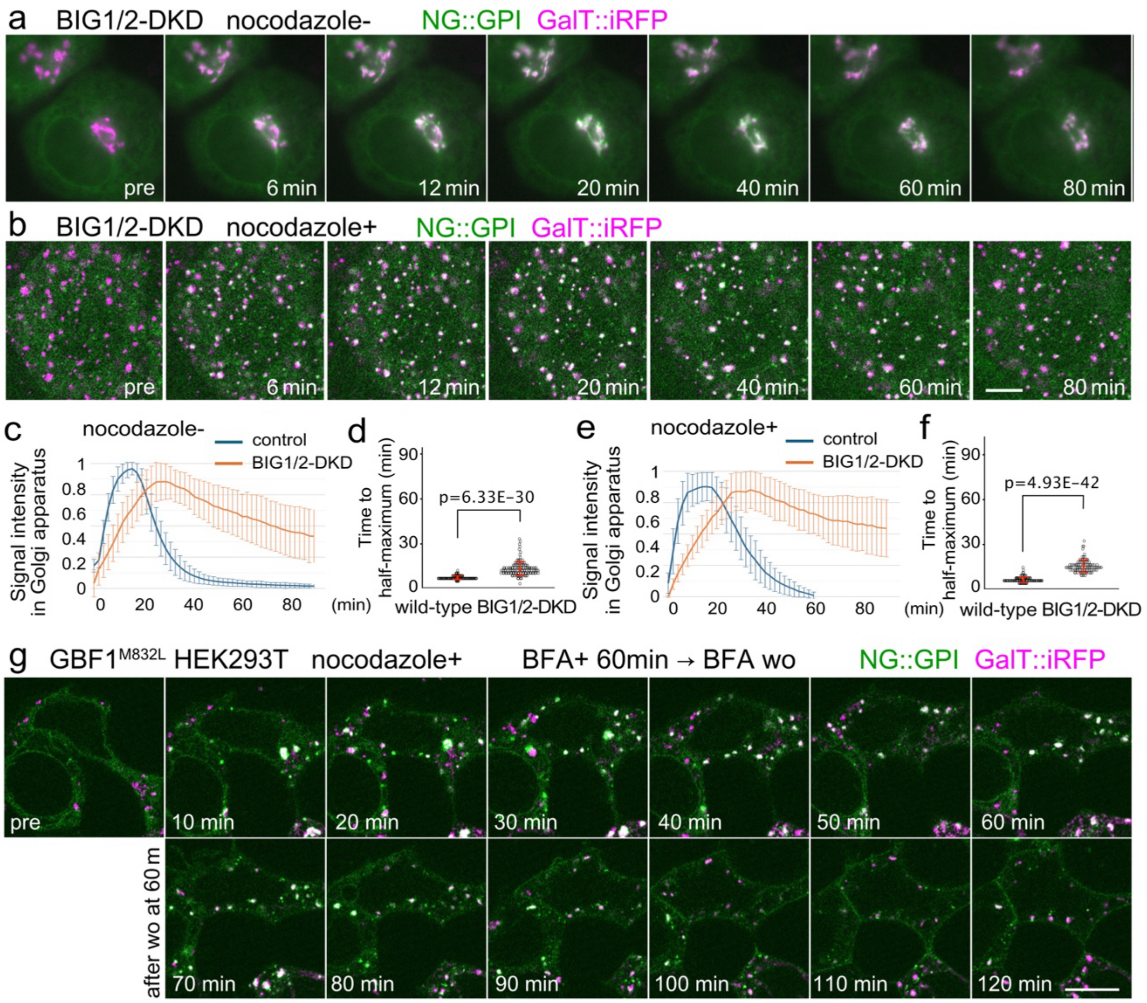

a
BIG1/2-DKD nocodazole- NG::GPI GalT::iRFP
pre
6 min
12 min
20 min
40 min
60 min
80 min
b
BIG1/2-DKD nocodazole+ NG::GPI GalT::iRFP
pre
6 min
12 min
20 min
40 min
60 min
80 min
c
nocodazole-
control
BIG1/2-DKD
Signal intensity in Golgi apparatus
(min)
d
Time to half-maximum (min)
p=6.33E-30
wild-type
BIG1/2-DKD
e
nocodazole+
control
BIG1/2-DKD
Signal intensity in Golgi apparatus
(min)
f
Time to half-maximum (min)
p=4.93E-42
wild-type
BIG1/2-DKD
g
GBF1M832L HEK293T nocodazole+ BFA+ 60min → BFA wo NG::GPI GalT::iRFP
pre
10 min
20 min
30 min
40 min
50 min
60 min
after wo at 60 m
70 min
80 min
90 min
100 min
110 min
120 min

Supplementary Figure 6. BFA reversibly inhibits cargo exit from Golgi stacks in nocodazole-treated GBF1^{M832L} HEK293T cells

a, b Time-lapse images showing NG::GPI transport initiated by the BME-RUSH system in MT-intact (a) and MT-disrupted (b) BIG1/2-DKD cells. NG::GPI is shown in green, and GalT::iRFP is shown in magenta.

c–f Quantification of cargo transport kinetics:

The wild type data are the same as those shown in Fig. 7l, p.

c, e Relative signal intensity of NG::GPI in the Golgi apparatus over time after BME administration in MT-intact (c) and MT-disrupted (e) cells. Blue and orange lines represent wild-type and BIG1/2-DKD cells, respectively. The Golgi apparatus is identified by the GalT::iRFP signal. The following numbers of cells were analyzed: MT-intact wild-type cells, n = 101, MT-intact BIG1/2-DKD cells, n = 132, MT-disrupted wild-type cells, n = 100, and MT- disrupted BIG1/2-DKD cells, n = 99.

d, f Time constants for NG::GPI accumulation in the Golgi apparatus. The time constants for MT-intact wild-type, MT-intact BIG1/2-DKD cells, MT-disrupted wild-type, and MT-disrupted BIG1/2-DKD cells were 6.5 ± 1.4, 13.4 ± 5.3, 4.8 ± 2.1, and 15.0 ± 4.7 min, respectively. Statistical analysis was performed using a one-sided two-sample t-test assuming unequal variances (Welch's t-test).

g Time-lapse images showing NG::GPI transport initiated by the BME-RUSH system in BFA-treated and washed-out GBF1^{M832L} HEK293T cells. BFA and BME treatments were initiated after a 4-hour incubation with nocodazole. BFA was washed out 60 min after BME addition (bottom). NG::GPI is shown in green, and GalT::iRFP is shown in magenta. Four cells were observed.

Scale bars: 10 μm (a, b, g).

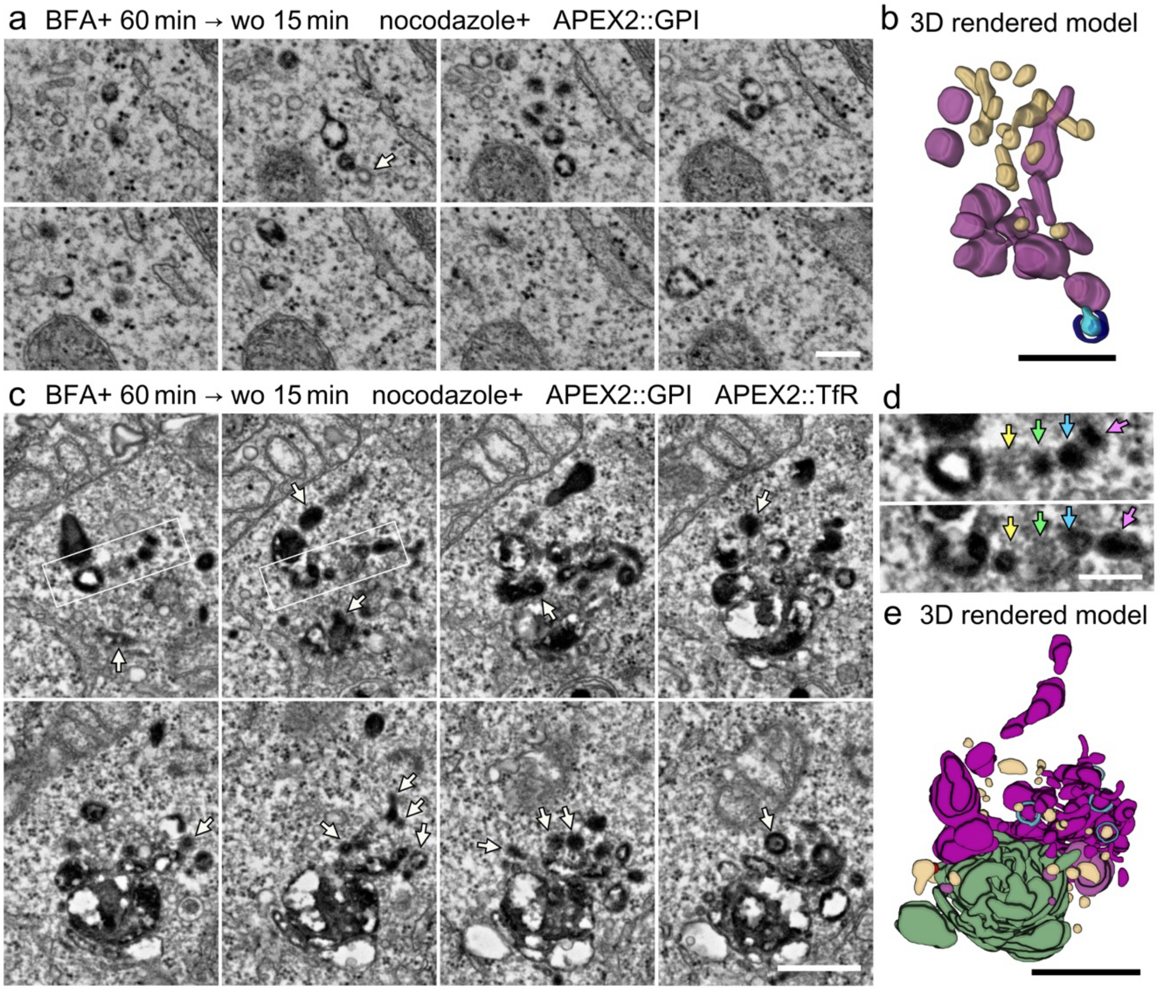

a
BFA+ 60 min → wo 15 min nocodazole+ APEX2::GPI
b
3D rendered model
c
BFA+ 60 min → wo 15 min nocodazole+ APEX2::GPI APEX2::TfR
d
e
3D rendered model

Supplementary Figure 7. Clathrin bud without GPI-AP connected to GPI-AP-positive cisternae

a Scanning electron micrograph of serial sections of APEX2::GPI-positive vesicle clusters at 50-nm intervals in a GBF1^{M832L} cell. BFA was added 5 min after BME administration, washed out after 60 min, and fixed after 15 min. The white arrow indicates a clathrin bud without GPI-AP. Ten cells were observed in TEM and four cells were observed in SEM.

b 3D images constructed from serial sections. APEX2::GPI-positive vesicles and tubules are shown in purple; APEX2::GPI-negative vesicles are shown in yellow; and a clathrin-coated bud without GPI-AP are shown in light blue; and clathrin is shown in dark blue.

c, d Scanning electron micrograph of serial sections at 50-nm intervals in a GBF1^{M832L} cell expressing luminal APEX2::GPI and cytoplasmic AREX2 fused with TfR (APEX2::TfR). BFA was added 5 min after BME administration, washed out after 60 min, and the cells were fixed 15 min later. Cells were pretreated with nocodazole for 4 h. Arrows indicate membranes positive for both APEX2::GPI and APEX2::TfR (c). Magnified images of the insets in (c) are shown in (d). Vesicles indicated by arrows of the same color represent the same vesicle in different sections. Two cells were observed using TEM and two using SEM.

e 3D images reconstructed from serial sections. Golgi stacks are shown in green; membranes positive for both APEX2::GPI and APEX2::TfR are shown in purple; membranes positive only for APEX2::GPI are shown in pink; unlabeled membranes are shown in yellow; and clathrin is shown in blue.

Scale bars: 200 nm (a, b), 500 nm (c), 200 nm (d), and 500 nm (e)

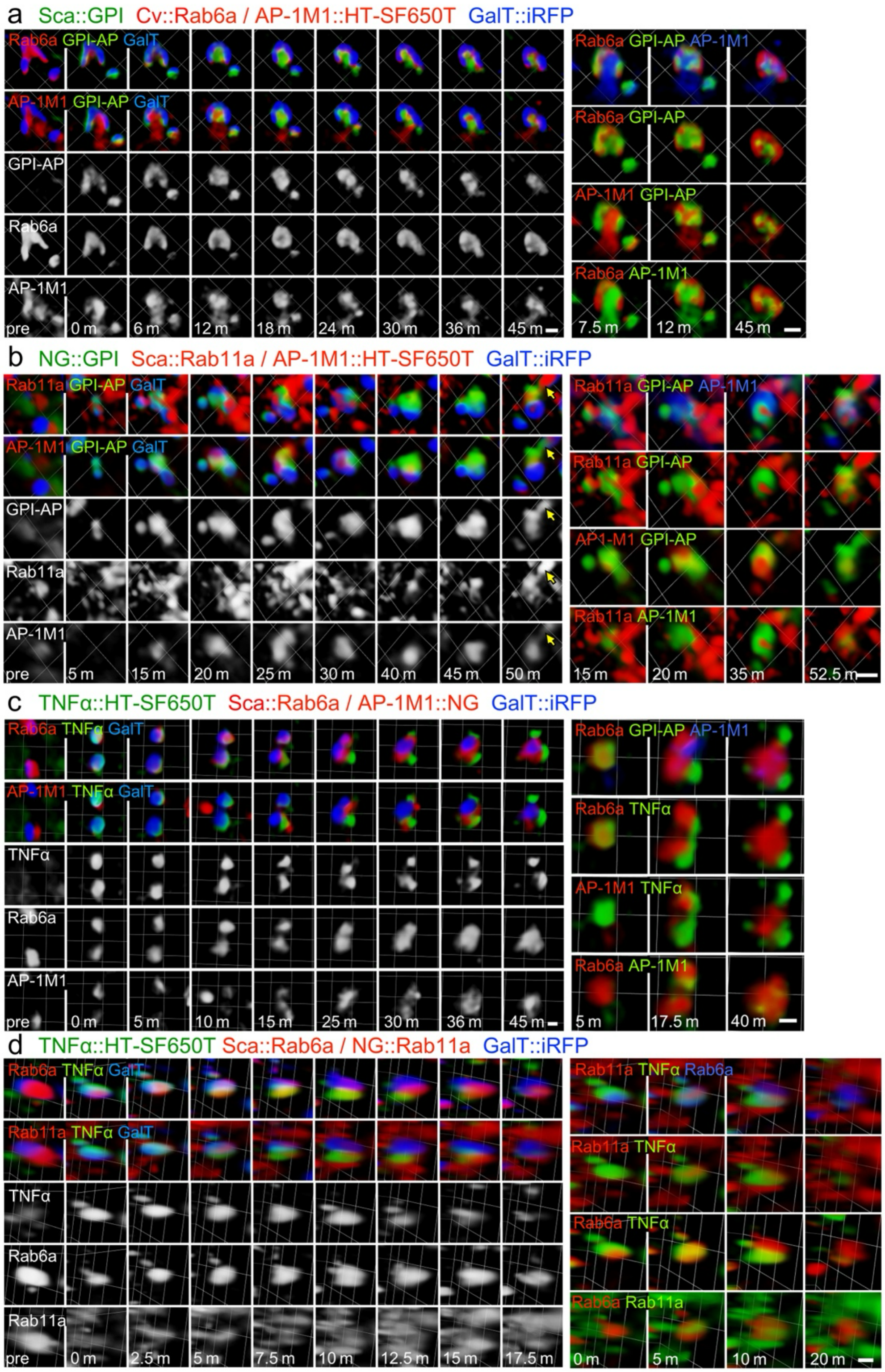
a
Sca::GPI Cv::Rab6a / AP-1M1::HT-SF650T GalT::iRFP
Rab6a GPI-AP GalT
AP-1M1 GPI-AP GalT
GPI-AP
Rab6a
AP-1M1
pre
0 m
6 m
12 m
18 m
24 m
30 m
36 m
45 m
Rab6a GPI-AP AP-1M1
Rab6a GPI-AP
AP-1M1 GPI-AP
Rab6a AP-1M1
7.5 m
12 m
45 m
b
NG::GPI Sca::Rab11a / AP-1M1::HT-SF650T GalT::iRFP
Rab11a GPI-AP GalT
AP-1M1 GPI-AP GalT
GPI-AP
Rab11a
AP-1M1
pre
5 m
15 m
20 m
25 m
30 m
40 m
45 m
50 m
Rab11a GPI-AP AP-1M1
Rab11a GPI-AP
AP1-M1 GPI-AP
Rab11a AP-1M1
15 m
20 m
35 m
52.5 m
c
TNFα::HT-SF650T Sca::Rab6a / AP-1M1::NG GalT::iRFP
Rab6a TNFα GalT
AP-1M1 TNFα GalT
TNFα
Rab6a
AP-1M1
pre
0 m
5 m
10 m
15 m
25 m
30 m
36 m
45 m
Rab6a GPI-AP AP-1M1
Rab6a TNFα
AP-1M1 TNFα
Rab6a AP-1M1
5 m
17.5 m
40 m
d
TNFα::HT-SF650T Sca::Rab6a / NG::Rab11a GalT::iRFP
Rab6a TNFα GalT
Rab11a TNFα GalT
TNFα
Rab6a
Rab11a
pre
0 m
2.5 m
5 m
7.5 m
10 m
12.5 m
15 m
17.5 m
Rab11a TNFα Rab6a
Rab11a TNFα
Rab6a TNFα
Rab6a Rab11a
0 m
5 m
10 m
20 m

Supplementary Figure 8. GPI-AP and TNFα transport observed using RudLOV

Observation of cargo transport using RudLOV. Cargo transport was initiated via 5 min of illumination at 445 nm. The frame before illumination is labeled "pre," and the frame immediately after illumination is labeled "0 min." Colors indicate the following four proteins:

a Sca::GPI, Cv::Rab6a, AP-1M1::HT-SF650T, and GalT::iRFP. Two cells were observed.

b NG::GPI, Sca::Rab11a, AP-1M1::HT-SF650T, and GalT::iRFP. Two cells were observed.

c TNFα::HT-SF650T, Sca::Rab6a, AP-1M1::NG, and GalT::iRFP. Two cells were observed.

d TNFα::HT-SF650T, Sca::Rab6a, NG::Rab11a, and GalT::iRFP. Two cells were observed.

Scale bars: 1 µm (a–d)

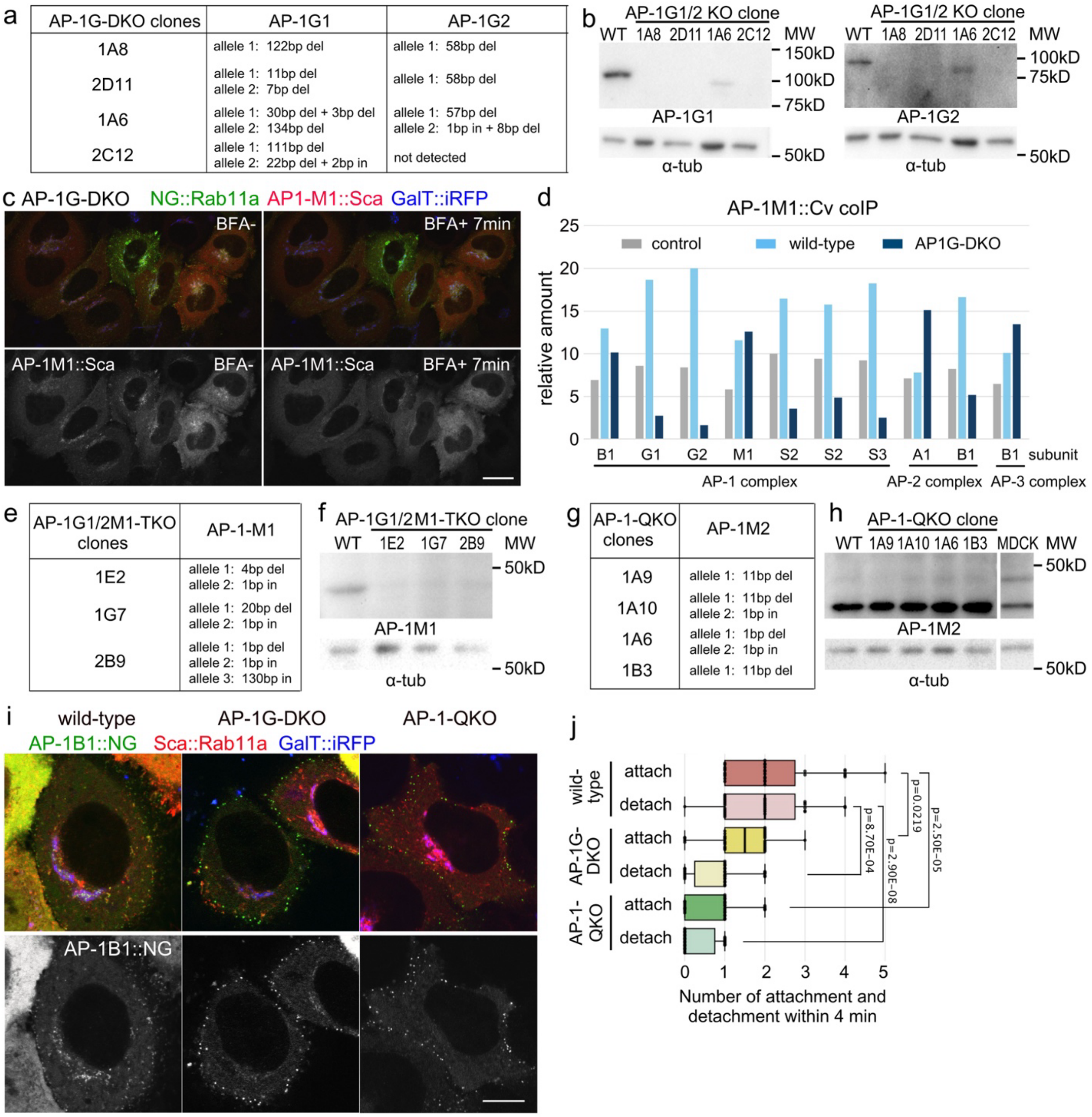

a
AP-1G-DKO clones
AP-1G1
AP-1G2
1A8
allele 1: 122bp del
allele 1: 58bp del
2D11
allele 1: 11bp del
allele 2: 7bp del
allele 1: 58bp del
1A6
allele 1: 30bp del + 3bp del
allele 2: 134bp del
allele 1: 57bp del
allele 2: 1bp in + 8bp del
2C12
allele 1: 111bp del
allele 2: 22bp del + 2bp in
not detected
b
AP-1G1/2 KO clone
WT 1A8 2D11 1A6 2C12 MW
150kD
100kD
75kD
AP-1G1
50kD
α-tub
AP-1G1/2 KO clone
WT 1A8 2D11 1A6 2C12 MW
100kD
75kD
AP-1G2
50kD
α-tub
c AP-1G-DKO NG::Rab11a AP1-M1::Sca GalT::iRFP
BFA-
BFA+ 7min
AP-1M1::Sca BFA-
AP-1M1::Sca BFA+ 7min
d
AP-1M1::Cv coIP
control
wild-type
AP1G-DKO
relative amount
0
5
10
15
20
25
B1 G1 G2 M1 S2 S2 S3 A1 B1 B1 subunit
AP-1 complex
AP-2 complex
AP-3 complex
e
AP-1G1/2M1-TKO clones
AP-1-M1
1E2
allele 1: 4bp del
allele 2: 1bp in
1G7
allele 1: 20bp del
allele 2: 1bp in
2B9
allele 1: 1bp del
allele 2: 1bp in
allele 3: 130bp in
f
AP-1G1/2M1-TKO clone
WT 1E2 1G7 2B9 MW
50kD
AP-1M1
α-tub
50kD
g
AP-1-QKO clones
AP-1M2
1A9
allele 1: 11bp del
1A10
allele 1: 11bp del
allele 2: 1bp in
1A6
allele 1: 1bp del
allele 2: 1bp in
1B3
allele 1: 11bp del
h
AP-1-QKO clone
WT 1A9 1A10 1A6 1B3 MDCK MW
50kD
AP-1M2
α-tub
50kD
i
wild-type
AP-1G-DKO
AP-1-QKO
AP-1B1::NG Sca::Rab11a GalT::iRFP
AP-1B1::NG
j
wild-type
attach
detach
AP-1G-DKO
attach
detach
AP-1-QKO
attach
detach
p=0.0219
p=8.70E-04
p=2.90E-08
p=2.50E-05
0 1 2 3 4 5
Number of attachment and detachment within 4 min

Supplementary Figure 9. Construction of AP-1 deficient cells and analysis of cargo transport

a List of AP-1G-double knockout (DKO) candidate clones.

b Immunoblotting of extracts prepared from the wild-type and AP-1G-DKO candidate clones using anti-AP-1G1, anti-AP-1G2, and anti-α-tubulin antibodies. 1A8 was selected as AP-1G-DKO cells.

c AP-1M1::Sca localization before (left) and 7 min after (right) BFA administration in AP-1G-DKO cells. AP-1M1::Sca is shown in red; GalT::iRFP is shown in blue; and NG::Rab11a is shown in green. The bottom panel shows black-and-white single channel images of AP-1M1::Sca. Some AP-1M1 was localized to the TGN in AP-1G-DKO cells. Fifteen cells were observed.

d Liquid chromatography-mass spectrometry analysis of the co-immunoprecipitated products of wild-type (light blue) and AP-1G-DKO (dark blue) cells expressing AP-1M1::Cv using an anti-GFP antibody. Wild-type cells not expressing AP-1M1::Cv were used as controls (gray).
All AP-1 subunits, as well as the B1 subunits of AP-2 and AP-3, co-immunoprecipitated in wild-type cells. However, only AP-1B1, AP-2A1, and AP-3B1 co-immunoprecipitated in AP-1G-DKO cells, whereas the other AP-1s were detected at low levels only. The AP-2A1 subunit was present in higher amounts in AP-1G-DKO cells than in wild-type cells, suggesting that it formed an AP1-like complex instead of an AP-1G1/2 complex. Thus, AP-1G-DKO cells lack the AP-1G1 and AP-1G2 subunits, yet still exhibit some AP-1 complex activity. However, the extent of this activity is likely to be limited.

e List of AP-1G1/2M1-triple knockout (TKO) candidate clones.

f Immunoblotting of extracts prepared from the wild-type and AP-1G1/2M1-TKO candidate clones using anti-AP-1M1 and anti-α-tubulin antibodies.

g List of AP-1-quadruple knockout (QKO) candidate clones.

h Immunoblotting of extracts prepared from the wild-type and AP-1-QKO candidate clones using anti-AP-1M2 and anti-α-tubulin antibodies. 1A9 was selected as AP-1-QKO cells. We also used an MDCK cell extract as a positive control. AP-1M2 is expressed in MDCK cells, but not in HeLa cells.

i AP-1B1::NG localization (green) in wild-type, AP-1G-DKO, and AP-1-QKO cells. Sca::Rab11a is shown in red; GalT::iRFP is shown in blue. The bottom panel shows black-and-white single channel images of AP-1B1::NG. In wild-type cells, AP-1B1 was localized on the *trans* side of the Golgi apparatus, presumably in the TGN. Notably, AP-1B1 was also detected in the plasma membrane, suggesting that it was incorporated into the AP-2 complex. AP-1B1 was weakly detected

in the TGN of AP-1G-DKO cells but was absent in AP-1-QKO cells. Localization to the plasma membrane was detected in both AP-1G-DKO and AP-1-QKO cells. Three wild-type, three AP-1G-DKO, and two AP-1-QKO cells were observed.

j Plot showing the number of RE attachments to and detachments from Golgi stacks within 4 min in wild-type, AP-1G-DKO and AP-1-QKO cells. The box plots show the median and the interquartile range (IQR), while whiskers extend to 1.5 times the interquartile range. Minimum or maximum values are used instead when whiskers exceed minimum or maximum. Statistical analyses were performed using a two-sided Welch's t test with Holm adjustment for multiple comparisons. Thirty Golgi stacks were counted for each condition.

Scale bars: 20 μm (c) and 10 μm (i)

Takiguchi et al., Supplementary Figure 10

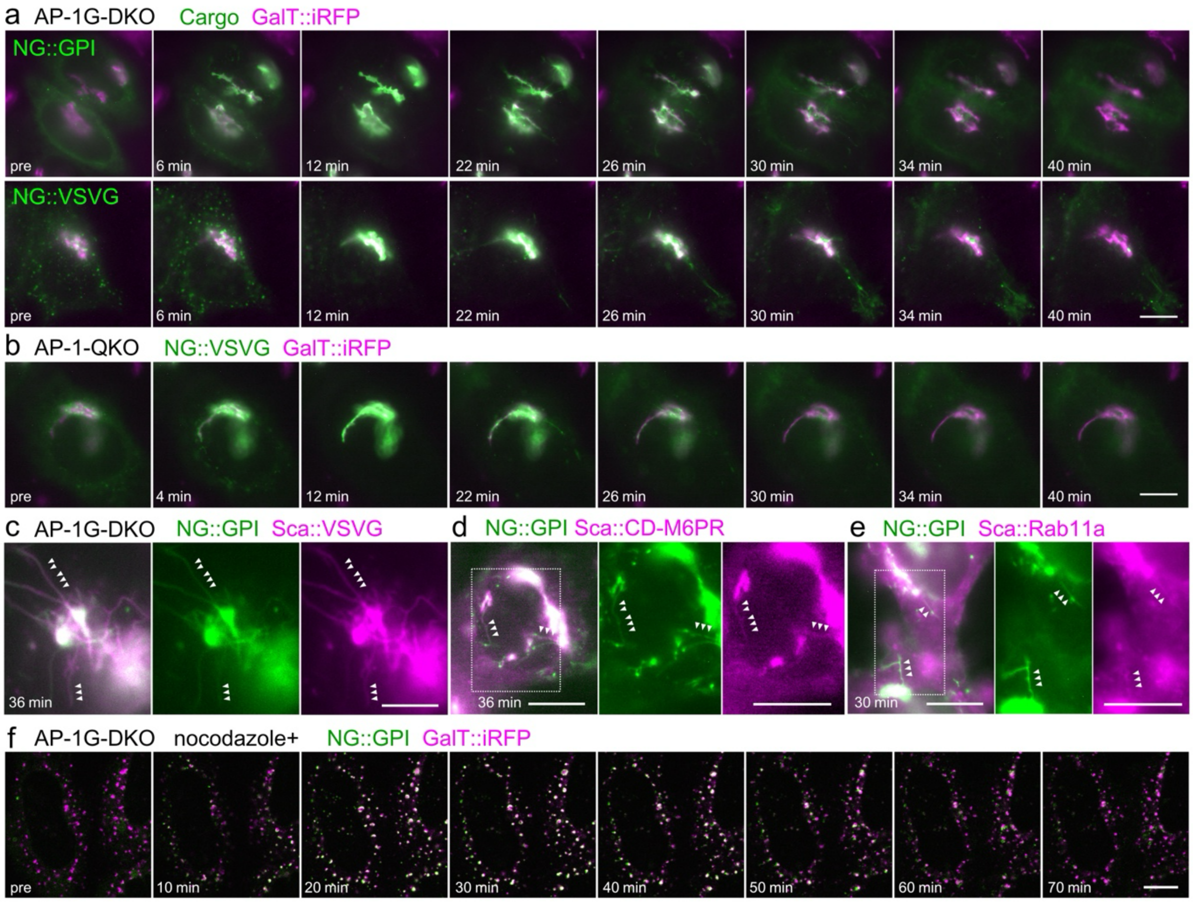

Supplementary Figure 10. Cargo transport in AP-1G-DKO and AP-1-QKO cells

a Time-lapse images showing NG::GPI (top) and NG::VSVG (bottom) transport initiated by the BME-RUSH system in AP-1G-DKO cells. The cargo is shown in green, and GalT::iRFP is shown in magenta.

b Time-lapse images showing NG::VSVG (green) transport initiated by the BME-RUSH system in AP-1-QKO cells. GalT::iRFP is shown in magenta.

c–e Co-localization studies in AP-1G-DKO cells: NG::GPI (green) and Sca::VSVG (magenta) localization 36 min after BME administration (c), NG::GPI (green) and Sca::M6PR (magenta) localization 36 min after BME administration (d), NG::GPI (green) and Sca::Rab11a (magenta) localization 30 min after BME administration (e). The right panels in (d) and (e) show magnified single-color images of the insets of the left panels. Arrowheads indicate co-localization of cargo pairs (c, d) or cargo with Rab11a (e). The following numbers of cells were analyzed: NG::GPI vs Sca::VSVG: n=8, NG:: VSVG vs Sca::M6PR: n=9, NG::GPI vs Sca::Rab11a: n=5.

f Time-lapse images showing NG::GPI transport initiated by the BME-RUSH system in AP-1G-DKO cells. BME treatment was initiated after 4 h of incubation with nocodazole. NG::GPI is shown in green, and GalT::iRFP is shown in magenta.

Scale bars: 10 µm (a, b), 5 µm (b–e), and 10 µm (f)

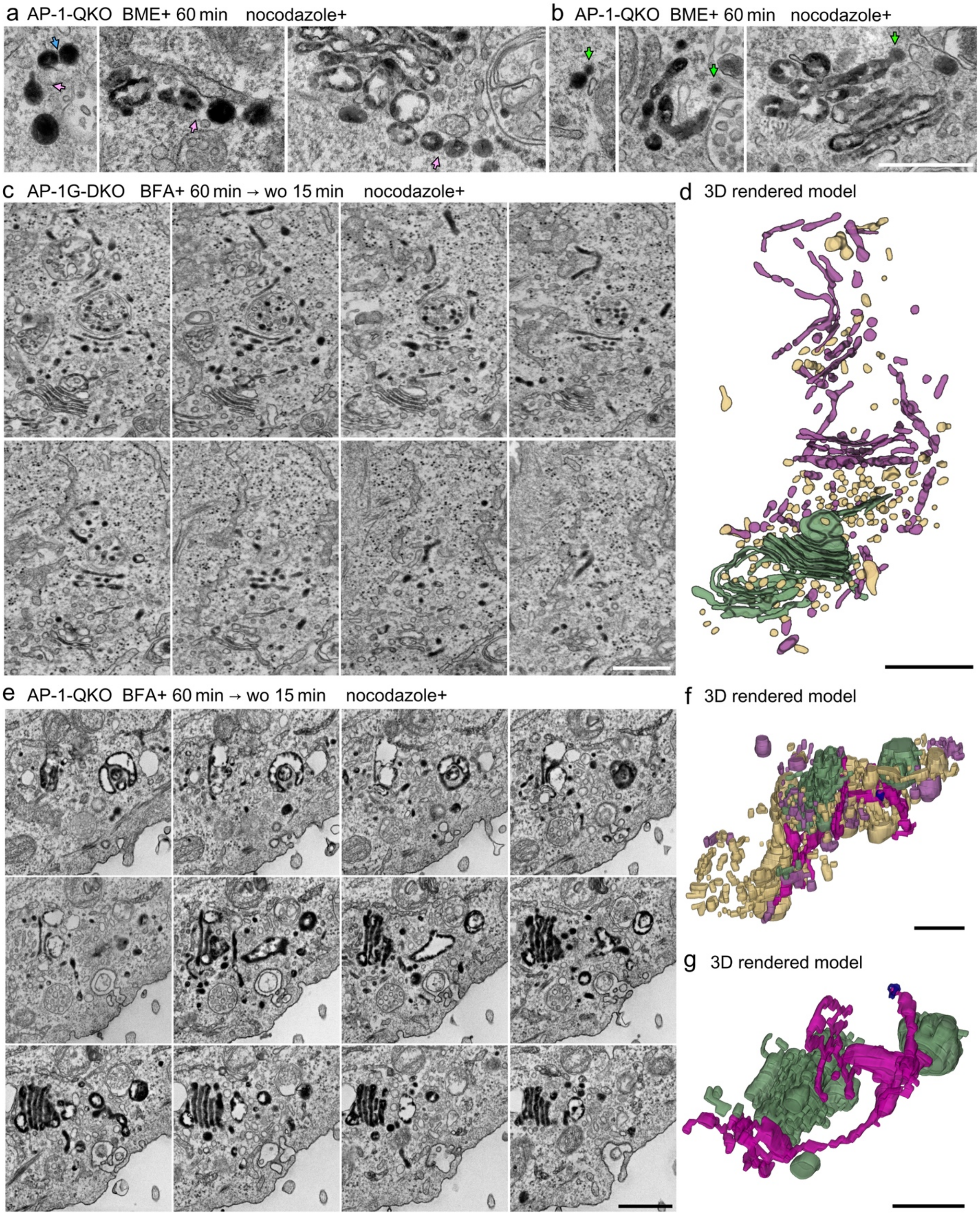
a AP-1-QKO BME+ 60 min nocodazole+
b AP-1-QKO BME+ 60 min nocodazole+
c AP-1G-DKO BFA+ 60 min → wo 15 min nocodazole+
d 3D rendered model
e AP-1-QKO BFA+ 60 min → wo 15 min nocodazole+
f 3D rendered model
g 3D rendered model

Supplementary Figure 11. GPI-AP localization in AP-1G-DKO and AP-1-QKO cells

a, b Transmission electron micrographs of nocodazole-treated AP-1QKO cells treated with APEX2::GPI, in which transport was initiated using the BME-RUSH system and fixed 60 min after BME administration. Blue arrows indicate hemifused vesicles containing APEX2::GPI, and pink arrows indicate candidate bead-like structures containing APEX2::GPI. Green arrows indicate clathrin-coated buds containing APEX2::GPI. Four cells were observed using TEM and four using SEM.

c–g Scanning electron micrographs of serial sections at 50-nm intervals of Golgi stacks, tubules, and vesicles in nocodazole-treated AP-1G-DKO (c) and AP-1-QKO cells (e) 15 min after BFA washout, and their 3D images reconstructed from serial sections (d, f, g). APEX2::GPI transport was initiated using the BME-RUSH system, and BFA was added 5 min after BME administration. BFA was washed out after 55 min of incubation, and the cells were fixed 15 min after BFA washout. APEX2::GPI was visualized as electron-dense precipitates. In the 3D-rendered image, the Golgi stack is shown in green; APEX2::GPI-positive tubules connected to the Golgi stack are shown in purple; APEX2::GPI-positive tubules without connection to the Golgi stack are shown in pink; vesicles without GPI-AP are shown in yellow; and the clathrin coat is shown in dark blue. Only the Golgi stack (green) and APEX2::GPI-positive tubules connecting the Golgi membrane (purple) are shown in (g), at different angles from those in (f). The following numbers of cells were analyzed: AP-1G-DKO: n=9 (TEM) and 6 (SEM), AP-1-QKO: n=3 (TEM) and 4 (SEM).

Scale bars: 500 nm (a–g)

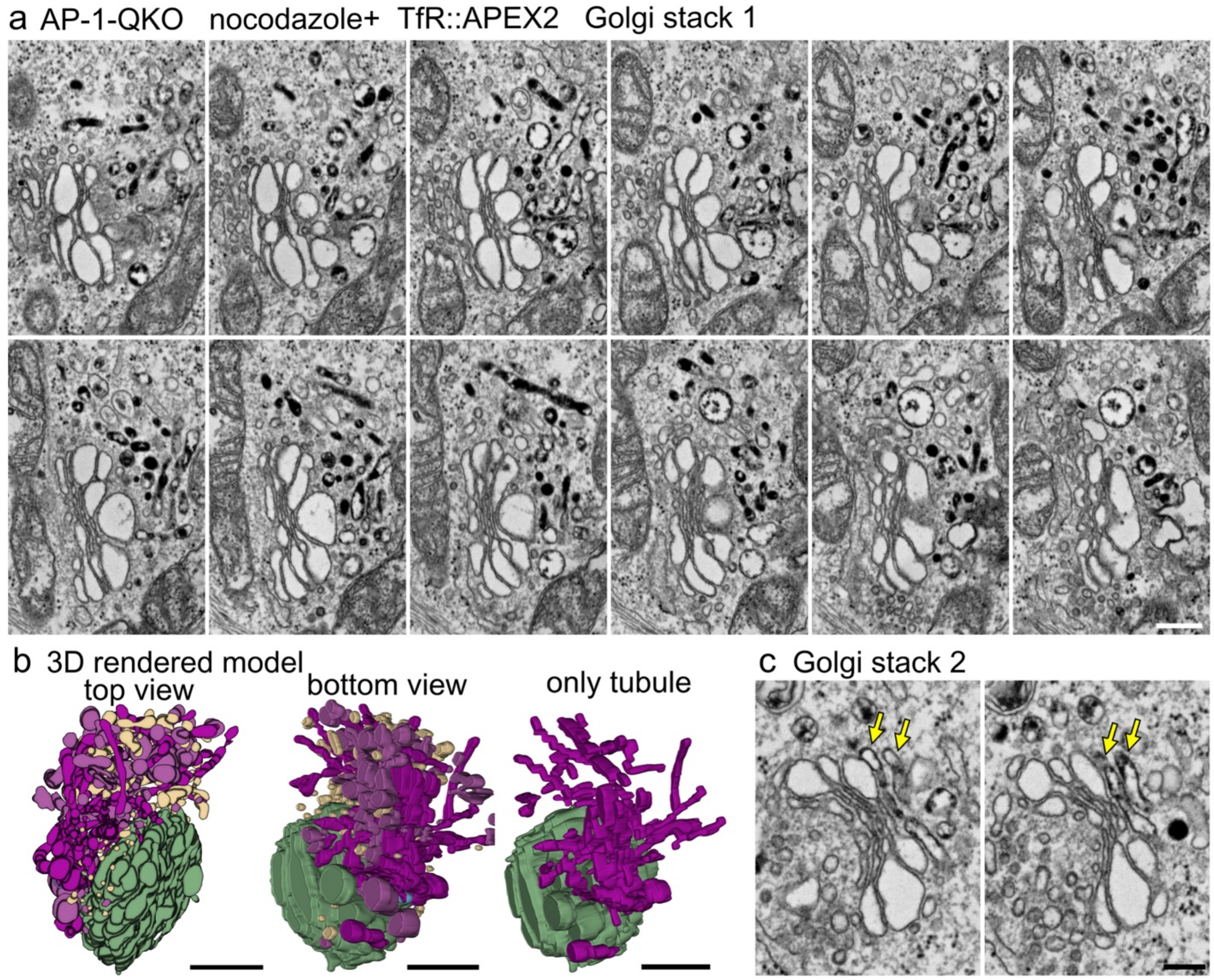

Supplementary Figure 12. TfR localization in AP-1-QKO cells

Scanning electron micrographs of serial sections at 50-nm intervals of Golgi stacks, tubules, and vesicles in nocodazole-treated AP-1-QKO cells (a, c), and their corresponding 3D rendered images (b). TfR::APEX2 (APEX2 localized in the lumen), visualized as electron-dense precipitates, indicates REs. Yellow arrows indicate TfR::APEX2-positive cisternae (c). In the 3D-rendered images (b), Golgi stacks are shown in green; TfR::APEX2-positive tubules connected to the Golgi stack are shown in purple; TfR::APEX2-positive tubules not connected to the Golgi stack are shown in pink; and vesicles lacking TfR::APEX2 are shown in yellow. Only the Golgi stacks (green) and TfR::APEX2-positive tubules (purple) are shown in the right panel of (b). Two cells were observed in TEM and three cells were observed in SEM.

Scale bars: 500 nm (a–c)
