# Supplementary material for: Reversible control of post-Golgi transport by brefeldin A reveals recycling endosome maturation during glycosylphosphatidylinositol-anchored protein transport
Source: Nat Commun. 2026 Jul 27;17:7262. doi: 10.1038/s41467-026-75784-1 (PMC13408501; doi:10.1038/s41467-026-75784-1)
Supplement: Supplementary file 2 — Description of Additional Supplementary Files [file 41467_2026_75784_MOESM2_ESM.pdf]

Title: Supplementary Data 1

Description: Plasmids, primers, and template information.

Title: Supplementary Video 1. Brefeldin A (BFA) induces Golgi absorption in wild-type cells, but not in $GBF1^{M832L}$ cells

Description: This video consists of three independent movies. The first movie shows the Golgi apparatus in wild-type HeLa cells on the left and $GBF1^{M832L}$ HeLa cells on the right before and after BFA administration. The second movie shows the Golgi apparatus and recycling endosomes (REs). The following colors are used to highlight different markers: green for the RE marker (SG::Rab11a), red for the *medial*-Golgi marker (ManII::HT-JF549), and blue for the *trans*-Golgi marker (GalT::iRFP). The third movie shows the Golgi apparatus before (0 min) and after BFA administration in wild-type HEK293T cells on the left and $GBF1^{M832L}$ HEK293T cells on the right. In this movie, green indicates the endoplasmic reticulum marker (SG::KDEL), whereas magenta indicates the *trans*-Golgi marker (GalT::iRFP).

Scale bars: 10 μm

Title: Supplementary Video 2. Dynamics of REs are reduced by BFA administration in MT-disrupted $GBF1^{M832L}$ cells

Description: RE dynamics in MT-disrupted $GBF1^{M832L}$ cells before (left) and after (right) BFA administration are shown. GalT::iRFP is shown in magenta, and the RE marker (NG::Rab11a) is shown in green. Each frame is a volume-rendered 3D image captured at 5-second intervals and captured using confocal live imaging microscopy (SCLIM).

Grid: 1.51 (left) or 1.57 (right) μm

Title: Supplementary Video 3. Accumulation of vesicles on the *trans* side of Golgi stacks in BFA-treated $GBF1^{M832L}$ cells

Description: Scanning electron micrographs of serial sections with a 50-nm interval of a Golgi stack in a $GBF1^{M832L}$ cell after 60 min of incubation with (right) or without (left) 10 μM BFA. Arrows indicate hemifused vesicles. Arrowheads indicate constricted connections between vesicles. Portions of the frames are shown in Fig. 2h, i.

Scale bar: 500 nm

Title: Supplementary Video 4. TfR localization BFA-treated and untreated GBF1^{M832L} cells

Description: Scanning electron micrographs of serial sections of a Golgi stack at 50-nm intervals in GBF1^{M832L} cells expressing TfR::APEX2 (APEX2 localized in the lumen) with or without BFA treatment. Golgi stacks are indicated in green, whereas TfR-positive and negative membranes are indicated in pink and yellow, respectively. Clathrin coats are shown in blue. TfR positive membranes connected to Golgi stacks are shown in purple. Portions of the frames are shown in Supplementary Fig. 4a–d.

Scale bar: 200 nm

Title: Supplementary Video 5. Cargo transport in Golgicide A (GCA)- or BFA-treated GBF1^{M832L} cells

Description: Time-lapse movies showing NG::GPI, NG::VSVG, and TNFα::NG transport initiated by the BME-RUSH system in untreated (left), GCA-treated (middle), and BFA-treated (right) cells. The cargoes are shown in green, and GalT::iRFP is shown in magenta. Portions of the frames are shown in Fig. 3a, b, and Supplementary Fig. 5a.

Scale bar: 10 μm

Title: Supplementary Video 6. Double cargo transport

Description: Time-lapse movies showing the transport of NG::VSVG (red) and Sca::GPI (green), or NG::VSVG (red) and TNFα::Sca (green), initiated by the BME-RUSH system. GalT::iRFP is indicated in blue. Portions of the frames are shown in Supplementary Fig. 5e. f.

Grid: 2.12 μm

Title: Supplementary Video 7. Cargo transport in nocodazole- and BFA-treated GBF1^{M832L} cells

Description: The time-lapse movies show NG::GPI or TNFα::NG transport initiated by the biotin methyl ester (BME)-RUSH system in untreated (left), BFA-treated (middle), and BFA-treated cells with washout (right). NG::GPI or TNFα::NG is shown in green, and GalT::iRFP is shown in magenta.

Scale bar: 10 μm

Title: Supplementary Video 8. GPI-AP transport in nocodazole-treated GBF1^{M832L} HEK293T cells

Description: Time-lapse movies showing NG::GPI transport initiated by the BME-RUSH system BFA-treated and washout conditions in GBF1^{M832L} HEK293T cells. NG::GPI is shown in green, and GalT::iRFP is shown in magenta. Portions of the frames are shown in Supplemental Fig. 6g.

Scale bar: 10 μm

Title: Supplementary Video 9. ARF recruitment after BFA washout in GBF1^{M832L} cells

Description: Time-lapse movies showing the recruitment of ARF1::NG or ARF3::NG (green) to the Golgi/RE unit and the efflux of Sca::GPI (red) from the Golgi/RE unit after BFA washout. BFA was added 5 min after BME administration and washed 55 min later. GalT::iRFP is indicated in blue. Portions of the frames are shown in Fig. 5m, n.

Grid: 2.12 μm

Title: Supplementary Video 10. GPI-AP transport into REs after BFA-washout in GBF1^{M832L} cells

Description: The high-speed time-lapse movies show the RUSH cargo HT::GPI-JF549 (red) transported into the REs after BFA washout. GalT::iRFP is shown in blue, and NG::Rab11a is shown in green. Numbers in the bottom-right corner represent the time after BFA washout. Each frame is a volume-rendered 3D image captured every 5 s and collected using SCLIM. Portions of the frames are shown in Fig. 5p, q.

Scale bar: 1 μm

Title: Supplementary Video 11. GPI-AP localization before and after BFA-washout in GBF1^{M832L} cells

Description: Scanning electron micrographs of serial sections of a Golgi stack at 50-nm intervals in GBF1^{M832L} cells expressing APEX2::GPI before and after BFA washout. Frames are shown in Fig. 6a–e, h–j and Supplementary Fig. 7a, b. The Golgi stacks are shown in green; APEX2::GPI-positive vesicles and tubules are shown in purple or pink; APEX2::GPI-negative vesicles are shown in yellow; APEX2::GPI-positive beads or tubule structures connected to the TGN are shown in purple

Scanning electron micrographs of serial sections of a Golgi stack at 50-nm intervals in GBF1^{M832L} cells expressing APEX2::GPI (APEX2 localized in the lumen) and APEX2::TfR (APEX2 localized in cytoplasm) 15 min after BFA washout. Portions of the frames are shown in Fig. 6f, g, k and Supplementary Fig. 7c, d.
Golgi stacks are shown in green; membranes positive for both APEX2::GPI and APEX2::TfR are shown in purple; membranes positive only for APEX2::GPI are shown in pink; membranes positive only for APEX2::TfR are shown in red ; unlabeled membranes are shown in yellow; and clathrin is shown in blue.
Scale bar: 200 nm

Title: Supplementary Video 12. AP-1 and clathrin are diffused and recruited by BFA administration and washout in GBF1^{M832L} cells
Description: Time-lapse videos showing NG::Clc (red), AP-1M1::Sca (green), and GalT::iRFP (blue) before and after BFA treatment and BFA washout. Portions of the frames are shown in Fig. 7a.
Grid: 2.11 μm

Title: Supplementary Video 13. AP-1 is recruited to the membrane with GPI-AP after BFA washout
Description: Time-lapse movies show AP-1M1::HT-SF650T (red, left), NG::GPI (green), GalT::iRFP (blue), and Sca::Rab11a (red, right) after BFA washout. Portions of the frames are shown in Fig. 7f, g.
Grid: 2.11 μm

Title: Supplementary Video 14. Cargo transport using RudLOV
Description: Time-lapse movies showing NG::GPI or TNFα::HT-SF650T transport. Cargo transport was initiated with illumination at 445 nm for 5 min using RudLOV. Portions of the frames are shown in Supplementary Fig. 8.
Grid: 2.11 μm

Title: Supplementary Video 15. RE dynamics are reduced in MT-disrupted AP-1 deficient cells

Description: Dynamics of REs in MT-disrupted wild-type (left), AP-1G-double knockout (DKO) (middle), and AP-1-quadrople knockout (QKO) (right) cells. GalT::iRFP is shown in magenta, and NG::Rab11a is shown in green. Each frame is a volume-rendered 3D image captured at 5-second intervals and collected using SCLIM. The movie was run at eight frames per second. Portions of the frames are shown in Fig. 7h, i.
Scale bar: 2 μm

Title: Supplementary Video 16. GPI-AP transport in nocodazole-treated and untreated AP-1 deficient cells
Description: The time-lapse movies show NG::GPI or NG::VSVG transport initiated by the BME-RUSH system in both nocodazole-treated and untreated wild-type (left), AP-1G-DKO (middle), and AP-1-QKO (right) cells. NG::GPI is shown in green, and GalT::iRFP is shown in magenta. Portions of the frames are shown in Fig. 7k, o and Supplementary Fig. 10a, b, f.
Scale bar: 10 μm

Title: Supplementary Video 17. GPI-AP transport in nocodazole-treated AP-1 deficient cells
Description: The high-speed time-lapse movies show the RUSH cargo HT::GPI-JF549 (red) transported into REs after BFA washout. GalT::iRFP is shown in blue, whereas NG::Rab11a is shown in green. Numbers in the bottom-right corner represent the time after BFA washout. Each frame is a volume-rendered 3D image captured every 5 s.
Grid: 2.17 μm

Title: Supplementary Video 18. GPI-AP localization in AP-1 deficient cells
Description: Scanning electron micrographs of serial sections of a Golgi stack at 50-nm intervals in AP-1G-DKO and AP-1-QKO cells expressing APEX2::GPI after BFA washout or without BFA treatment. Golgi stacks are shown in green; APEX2::GPI-positive tubules connected to the Golgi stack are shown in purple; APEX2::GPI-positive tubules not connected to the Golgi stack are shown in pink; vesicles lacking GPI-AP are shown in yellow; and the clathrin coats are shown in dark blue. Portions of the frames are shown in Fig. 8f–h, and Supplementary Fig. 11c-e. Scanning electron micrographs of serial sections of a Golgi stack at 50-nm intervals in AP-1-QKO cells expressing TfR::APEX2 (APEX2 localized in the lumen) 15 min after BFA washout. Golgi stacks are shown

in green; TfR::APEX2-positive tubules connected to the Golgi stack are shown in purple; TfR::APEX2-positive tubules not connected to the Golgi stack are shown in pink; and vesicles lacking TfR::APEX2 are shown in yellow. Portions of the frames are shown in Supplementary Fig. 12a.

Scale bar: 200 nm

Title: Supplementary Video 19. Models for GPI-AP transport in wild-type and AP-1 deficient cells

Description: The model on the left shows GPI-AP transport from the TGN to the RE in a wild-type cell. The RE matures on the TGN while incorporating GPI-AP (frame 2). A free RE attaches to newly formed RE containing GPI-AP on the TGN, becoming a Golgi-associated RE (GA-RE) (frame 3). GPI-AP then spreads throughout the entire GA-RE (frame 4). Formation of an AP-1/clathrin-coated vesicle on the TGN disconnects the TGN from the GA-RE (frame 5). The free RE subsequently detaches from the TGN, and the GA-RE becomes a free RE (frame 6). The AP-1/clathrin-coated vesicle may then return to the TGN (frame 7).

The model on the right shows GPI-AP transport from the TGN to the RE in an AP-1 deficient cell. The RE matures on the TGN while incorporating GPI-AP. RE motility, as well as attachment to and detachment from the TGN, is suppressed in AP-1-deficient cells. Thus, RE maturation proceeds without detachment, and GPI-AP-containing tubules connected to the TGN continue to elongate (frames 1–7).
