# Supplementary material for: Reversible control of post-Golgi transport by brefeldin A reveals recycling endosome maturation during glycosylphosphatidylinositol-anchored protein transport
Source: Nat Commun. 2026 Jul 27;17:7262. doi: 10.1038/s41467-026-75784-1 (PMC13408501; doi:10.1038/s41467-026-75784-1)
Supplement: Supplementary file 24 — Transparent Peer Review file [file 41467_2026_75784_MOESM24_ESM.pdf]

nature portfolio

Peer Review File

Reversible control of post-Golgi transport by brefeldin A reveals recycling endosome maturation during glycosylphosphatidylinositol-anchored protein transport

Corresponding Author: Dr Akiko Satoh

This file contains all reviewer reports in order by version, followed by all author rebuttals in order by version.

Version 0:

Reviewer comments:

Reviewer #1

(Remarks to the Author)
In this cell biology study, Takiguchi et al. presents a sophisticated and well-executed investigation of post-Golgi transport regulation using a brefeldin-controlled system in genetically engineered mammalian cells. The authors generate a brefeldin-resistant mutant cell line (GBF1-M832L), enabling reversible inhibition and reactivation of post-Golgi transport without compromising Golgi integrity, thereby achieving high temporal control over post-Golgi trafficking events. Using a combination of live-cell imaging, super-resolution microscopy, and electron microscopy, the authors demonstrate that GPI-anchored protein (GPI-AP) transport proceeds through maturation of trans-Golgi network (TGN) domains into Golgi-associated recycling endosomes (GA-REs), which subsequently detach as free recycling endosomes. The system described here provides a valuable tool for dissecting previously elusive post-Golgi mechanisms, including glycosylation, lipid remodeling, and cargo-specific maturation of individual GPI-APs.

Suggestions
Owing to the breadth of data and experimental approaches, the manuscript is information-dense and occasionally difficult to follow. A clear schematic model summarizing the key conclusions would help orient readers from a broader readership. Related to this point, some of the primary imaging data might be more appropriately placed in the Supplementary Figures to improve the overall readability of the main text.

Reviewer #2

(Remarks to the Author)
This manuscript presents a novel system for synchronizing cargo release from the recycling endosomes using BFA, cleverly utilizing a GBF1 point mutant to selectively block BIG1 and BIG2 for post-Golgi traffic control. The authors use this approach to investigate Golgi-associated recycling endosomes and GPI-anchored protein sorting, proposing that AP-1 helps these endosomes detach from the Golgi.

This reviewer’s assessment of the work is mixed: while the methodology is technically remarkable, original, and highly creative, the level of quantification is currently subpar for a study of this caliber. The assay itself is a brilliant idea, particularly the introduction of the mutation to address the need for better post-Golgi transport release assays, but the lack of robust quantification makes it difficult to fully assess major findings, such as the specific role of AP-1 or the sorting mechanisms of different cargoes.

Major Comments:
Given the combination of the GBF1 modification, nocodazole treatment, BFA, and cargo overexpression, this reviewer has concerns whether the cells are still behaving "normally." Localizing endogenous cargoes like M6PR or the transferrin receptor (perhaps via uptake) would help demonstrate that cellular functions remain intact under these conditions. It would also be important to validate the sorting findings using endogenous cargo alongside a "rushed" cargo. Alternatively, looking at two endogenous cargoes via staining after BFA washout to confirm they are behaving similarly to the RUSHed cargoes would provide valuable validation.

Regarding the choice of HeLa cells, what was the rationale for this line, and is the system easily applicable to other cell types?
Figures 1b-c: For the development of the GBF M382L line, what is the viability of the mutant versus WT without BFA? Showing individual values in the supplemental data, rather than just a ratio, would better establish cell health.
Figures 1j-k & Extended Data Figure 1: These require quantification of BIG1 and BIG2 colocalization at the Golgi. A quantified increase would reinforce the conclusions far more than the current images. Similarly, quantifying the colocalization of various ARFs with TfR or GalT in Extended Data Figure 1 is necessary. Some puncta seem to be colocalizing for ARF4 and ARF5 and GalT signal is hard to see.
Figures 1g,i,l,o: The quantification methods are unclear and the axes are currently missing labels. Some conditions show less than 100% signal at the Golgi peak, can the authors explain better how the data was expressed/normalized?
Figure 2f: The data on Rab11 puncta mobility is interesting, but it lacks the statistics required to show if the observed effect is significant.
Figures 3c, f, and i: Same comments as above for Figure 1: The quantification methods are unclear and the axes are currently missing labels. Some conditions show less than 100% signal at the Golgi peak, can the authors explain better how the data was expressed / normalized?
Figure 3f: Some tubules appear to be devoid of Rab11 signal. Are these tubules cargo-dependent, or do they represent distinct types?
Lines 198-200: The statement regarding the separation of GPI-AP and VSVG needs quantification to support the observation that VSVG and TNFα continue to co-localize.
Lines 201-202: For the claim about M6PR transport, it isn't clear if this is the authors' own data or a reference to previous studies; if the latter, it needs to be cited.
Figure 5 & Figure 7: These sections suffer from a lack of quantification and statistical analysis, which needs to be addressed to substantiate the claims.
Figure 5o, Why are the two ARF (1 and 3) behaving differently when observed by themselves (o) vs with cargo (m, n)? There seems to be a loss of both ARFs for a longer period of time. Again, the lack of quantification makes it difficult to assess.

Minor Points:
Lines 124-125: The description of ARF1 "disappearing on the puncta" is confusing. How does this differ from the diffusion seen with ARF3?
Line 183: Possible typo; should this read "ARF1"?
Lines 241-242: The wording about two types of cargoes being close to each other is vague, especially as only GPI-AP was mentioned in that paragraph.
The manuscript would benefit from a clearer transition for the investigation of AP-1 and clathrin. What is the rationale for looking at AP-1? Additionally, the standard "AP-1" (with a hyphen) nomenclature should be used throughout.
The use of the term “natural conditions” (line 299 and throughout) is inaccurate for a system involving genetic mutations, chemical inhibitors, and light-induced cargo release.

There is a typo in Extended Data Fig1: “e, f ARF::NG4 (e) “ it should be ARF4::NG. Same figure, maybe another typo in “The plots show the normalized mean values for 15 Golgi markers and ARF line profiles across the Golgi stack “ 15 seems to be not relevant.
Typo in Extended Data Fig3: AFR1 should be replaced with ARF1 throughout.

Reviewer #3

(Remarks to the Author)
By using BFA treatment in the newly generated GBF1M832L cell line and powerful live images, Arata Takiguchi et al established a unique system to manipulate and observe the post-Golgi transport of cargo trafficking in this manuscript. Previous study has reported that BFA-sensitive Sec71 regulates RE detachment from Golgi stacks and post-Golgi transport in Drosophila. Based on this, the authors constructed a BFA-resistant GBF1 allele (GBF1M832L) in HeLa cells to analyze GPI-AP transport from TGN to GA-RE. They further validated that Sec71 homologs (BIG1/2) specifically activated ARF1/3, but not ARF4/5. Meanwhile, they carefully examined RE motility in MT-intact or -disrupt conditions, and compared 3 different cargoes (GPI-AP, VSVG and TNFα) transport in this system. Finally, the authors provided the evidence that AP-1 was required for microtubule-independent post-Golgi transport through a tubule scission mechanism.
Overall, this study provided a detailed investigation of RE-mediated cargo transport by the impressive images of electronic microscope and 3D-rendered model. However, several issues regarding to the chemical treated assay remain to be addressed.
Major concerns:
1. This study used BFA to treat cell line. Although the data is interesting and impressive, the whole process is unnatural. Additional evidence, including interfering key factors of BFA targets by knockout assay, should be given to validate the data.
2. The authors performed many live-cell images. However, some images or videos quality are dubious: 1) in supplementary video 1, the signals were not clear due to low resolution; 2) in figs 1k and 2b, GalT::iRFP+ signals, as a marker of trans-Golgi network, should not present in extracellular matrix. These images should be revised or replaced with higher-quality, representative examples.
3. The authors constructed a single allelic GBF1M832L HeLa cell line by using CRISPR/Cas9-mediated knock-in method. However, the detailed information of this cell line was lacking, such as knock-in site, GBF1 protein level. Meanwhile, the authors should explain the reason of GBF1M832L cells were more resistant to BFA treatment.
4. BFA treatment paradigm is foundational for this study. Hence, the choices of specific time points (10, 30, 60 min) for

different experiments should be justified.
5. The manuscript lacked basic background introduction in many cases: 1) the reason of using MT-intact and MT-disrupt cells in this assay, because Golgi apparatus was totally fragmented in MT-disrupt cells, whether the results were convinced in these conditions; 2) the effect of GCA treatment on cells; 3) in the final part of the manuscript, the logical flow in the investigation of AP-1 feels slightly disconnected. Introducing a hypothesis or previous study is recommended.
6. The author should utilize this system in other mammalian cells to expand its broad utility.
Minor concerns:
1. The authors should add descriptive titles to all supplementary videos to enhance their clarity.
2. In some images, such as figs 1d and 1e, the authors use same color (gray) to label different signals was confusing. Using different colors to label distinct Golgi networks is recommended.
3. The authors should modify some images: 1) figs 1d and 1j should be clearly labeled to indicate which panels represent MT-intact or MT-disrupted conditions; 2) the authors should add boxes or arrowheads in left panels to identify the regions shown at higher magnification on the right panels in fig 1f-i.
4. It would be extremely helpful for readers if the authors draw a schematic work model to summarize this work.
5. Line 53, please extensively introduce the information of “other pathways”.
6. Line 183, the “AFR1” should be modified into “ARF1”.
7. Line 229, “but not significantly different”. The data should be “fig 4f”. please examine this result.

Reviewer #4

(Remarks to the Author)
This manuscript by Takiguichi and colleagues has investigated the trafficking of cargo from the TGN and especially the functional relationship between the TGN and recycling endosome during cargo transport. The work is certainly of interest to understand cargo transport and Golgi exit along the anterograde transport pathway. The establishment of a cell system to allow brefeldin A controlled cargo exit from Golgi stacks is a clever approach that allows the exit of cargo from the TGN to be investigated. However, I found the manuscript very dense with information and it was challenging to identify the key findings from the manuscript that represent the advance from the previous work by the Satoh laboratory (Fujii et al 2020, JCS). The major take home message from the exploitation of this cell system was not entirely clear to this reviewer. A cartoon depicting the new findings and conclusions would have been helpful. I think the paper could be dramatically shortened to enhance the readability of the paper and to focus on the key findings.

I had a number of specific concerns/issues relating to the clarity of the data, quantitation, and interpretation.

Additional concerns

1. An ER control marker is needed Fig. 1d.

2. Fig. 1h, 1i. The manuscript says that the “ localisation of golgin 97 remained unaffected”. One cannot conclude this from the optical images, but could say the relative spatial distribution of GM130. GalT and golgin 97 was very similar in BFA treated and untreated cells.

3. Line 105/106 states “ which have scattered Golgi stacks in front of each ER exit site….” What is the evidence for this conclusion?

4. Data on the localisation ARF4 and ARF5, line 121 and following, is contrary to some of the literature- at least for ARF4. W. Nakai, Y. Kondo, A. Saitoh, T. Naito, K. Nakayama, H.W. Shin, ARF1 and ARF4 regulate recycling endosomal morphology and retrograde transport from endosomes to the Golgi apparatus, Mol Biol Cell 24 (2013) 2570-2581. Also, ARF4 is a binding partner of Rab30, which is localised to the TGN and recycling endosomes (Zulkefli et al Exp Cell Res, 399 (2021):112442). These differences in the literature need to be reconciled.

5. In Fig. 2b, Rab11- labelled tubules are hard to see in BFA-treated cells.

6. Fig. 2C- quantitation of attachment and separation of Rab11-positive structures from Golgi structures should be provided, as this represents an important dataset for the conclusion.

7. Fig. 3g, 3h: how is the polarity of the Golgi determined in the EM images to conclude that the vesicle accumulation was on the trans side? For BFA treated samples it is not clear how the Golgi membranes were identified as the Golgi stack is greatly perturbed. How does the perturbation of the Golgi stack impact on the proposed conclusions of the paper?

8. Line 182: Please clarify why GCA did not alter the kinetics of GPI-AP transport- this reviewer found this confusing. GCA should inhibit ERES trafficking?

9. Lines 192-194. Conclusion that “numerous Rab11 + tubules simultaneously carried newly synthesised cargo” was clear for the GPI-AP cargo but not obvious for the other cargoes. Better images are needed.

10. Line 201- “M6PR was transported to endosomes rather than plasma membrane:” This finding may be expected but do the authors have data that this is the case in their manipulated cell system? No data appears to have been provided.

11. Fig extended data Fig. 3a-f are very small and difficult to interpret.

12. Figure 6B. CLEM is weak.

13. Figure 7e; quantitation is needed on the level of co-localisation of AP1 with GPI-AP and M6PR.

14. Extended Fig 5a- says cargo enters the cis side of Golgi, however, a cis-Golgi marker is not included. Also data at 9 min pulse is not shown in Supp Fig. 5, but is mentioned in text

15. Line 336. It was not clear to this reviewer why KO of AP-1 did not result in the accumulation of GPI-AP in the Golgi stacks (Fig. 7n-q).

16. Line 342 and Fig. 8: GPI-AP incorporation into recycling endosomes of AP1 KO cells after BFA washout was not convincing. There is very little overlap of green and red signals, just proximity.

Version 1:

Reviewer comments:

Reviewer #2

(Remarks to the Author)
The authors have appropriately addressed my comments and concerns. The quantification of the key assays is a major addition to the manuscript.

Reviewer #3

(Remarks to the Author)
Takiguchi et al. performed addtional experiments and revised the manuscript to well addressed all my concerns. I recommend the publication of this mansucript.

Reviewer #4

(Remarks to the Author)
The revised manuscript by Satoh and colleagues has provided detailed responses and addressed my concerns and suggestions. Quantitation has been significantly improved. The manuscript remains very dense with data, however, the information is required to validate the conclusions. Overall, an impressive body of work.

There are a few minor issues that need to be corrected.
1. Figure 1e no longer has GM130 as a marker in the revised manuscript. The text in results section and in the Figure legend need to be corrected, and replaced with KDEL.
2. Line 107-108. The statement that the “Golgi ribbon was slightly fragmented” cannot be concluded based on the resolution. It is more appropriate to state that the Golgi morphology was slightly perturbed.
3. Video 19 has been added in the revision to explain the model. Reference to this video is missing in the text and needs to be included.

Open Access This Peer Review File is licensed under a Creative Commons Attribution 4.0 International License, which permits use, sharing, adaptation, distribution and reproduction in any medium or format, as long as you give appropriate credit to the original author(s) and the source, provide a link to the Creative Commons license, and indicate if changes were made.
In cases where reviewers are anonymous, credit should be given to 'Anonymous Referee' and the source.
The images or other third party material in this Peer Review File are included in the article's Creative Commons license, unless indicated otherwise in a credit line to the material. If material is not included in the article's Creative Commons license and your intended use is not permitted by statutory regulation or exceeds the permitted use, you will need to obtain permission directly from the copyright holder.
To view a copy of this license, visit https://creativecommons.org/licenses/by/4.0/

Thank you for your suggestions.

We would first like to apologize for the errors in Figure 8a, b, d and Supplementary Figure 4e, f (previously Extended Data Figure 2b, c).

In the previous version of Figure 8, HT::GPI-JF549 was incorrectly labeled in green and NG::Rab11a in red; in fact, HT::GPI-JF549 is shown in red and NG::Rab11a in green. This labeling has now been corrected in the revised Figure 8. The figure legend was accurate in the previous version.

In Supplementary Figure 4e, f (previously Extended Data Figure 2b, c), NG::VSVG was incorrectly labeled in green and Sca::GPI/TNFα::Sca in red; however, NG::VSVG is actually shown in red and Sca::GPI/TNFα::Sca in green. These labeling errors have been corrected in the revised Supplementary Figure 4. In addition, the legend for this figure contained errors and has also been revised accordingly.

We also made several changes to the text to improve clarity.

Reviewer #1 (Remarks to the Author)

In this cell biology study, Takiguchi et al. presents a sophisticated and well-executed investigation of post-Golgi transport regulation using a brefeldin-controlled system in genetically engineered mammalian cells. The authors generate a brefeldin-resistant mutant cell line (GBF1-M832L), enabling reversible inhibition and reactivation of post-Golgi transport without compromising Golgi integrity, thereby achieving high temporal control over post-Golgi trafficking events. Using a combination of live-cell imaging, super-resolution microscopy, and electron microscopy, the authors demonstrate that GPI-anchored protein (GPI-AP) transport proceeds through maturation of trans-Golgi network (TGN) domains into Golgi-associated recycling endosomes (GA-REs), which subsequently detach as free recycling endosomes. The system described here provides a valuable tool for dissecting previously elusive post-Golgi mechanisms, including glycosylation, lipid remodeling, and cargo-specific maturation of individual GPI-APs.

Suggestions
Owing to the breadth of data and experimental approaches, the manuscript is information-dense and occasionally difficult to follow. A clear schematic model summarizing the key conclusions would help orient readers from a broader readership.
Related to this point, some of the primary imaging data might be more appropriately placed in the Supplementary Figures to improve the overall readability of the main text.

Thank you for this helpful suggestion.
We have added a schematic video (Supplementary Video 19) as well as a graphical abstract to clarify our model of post-Golgi transport of GPI-AP via recycling endosomes.

To improve readability and streamline the main text, we have reorganized several figures. Specifically, we removed the original Extended Data Figure 3 and the original Figure 7d.

At the same time, in response to comments from other reviewers, we have included additional datasets to strengthen the study. While this inevitably increases the overall volume of data, we have made every effort to present the results in a clearer and more accessible manner.

Reviewer #2 (Remarks to the Author):

This manuscript presents a novel system for synchronizing cargo release from the recycling endosomes using BFA, cleverly utilizing a GBF1 point mutant to selectively block BIG1 and BIG2 for post-Golgi traffic control. The authors use this approach to investigate Golgi-associated recycling endosomes and GPI-anchored protein sorting, proposing that AP-1 helps these endosomes detach from the Golgi.

This reviewer's assessment of the work is mixed: while the methodology is technically remarkable, original, and highly creative, the level of quantification is currently subpar for a study of this caliber. The assay itself is a brilliant idea, particularly the introduction of the mutation to address the need for better post-Golgi transport release assays, but the lack of robust quantification makes it difficult to fully assess major findings, such as the specific role of AP-1 or the sorting mechanisms of different cargoes.

Major Comments:
Given the combination of the GBF1 modification, nocodazole treatment, BFA, and cargo overexpression, this reviewer has concerns whether the cells are still behaving "normally."
Localizing endogenous cargoes like M6PR or the transferrin receptor (perhaps via uptake) would help demonstrate that cellular functions remain intact under these conditions.

We appreciate the reviewer's concern that the combination of GBF1 modification, nocodazole treatment, BFA treatment, and cargo overexpression may affect normal cellular behavior. These perturbations were intentionally introduced to achieve temporal control of cargo transport and thereby visualize trafficking events that are otherwise difficult to capture under steady-state conditions.

Importantly, several lines of evidence indicate that key trafficking pathways remain functional under our experimental conditions. First, although nocodazole disrupts microtubule-dependent transport, the rates of Golgi entry and exit of GPI-AP released by BME-RUSH are comparable (Fig. 4d), consistent with previous work showing that secretion can proceed in a microtubule-independent manner (Fourriere et al., 2016).

Fourriere, L., Divoux, S., Roceri, M., Perez, F. and Boncompain, G. (2016). Microtubule-independent secretion requires functional maturation of Golgi elements. J Cell Sci 129, 3238-50.

Second, to minimize potential artifacts due to cargo overexpression, we compared the RUSH system with the RudLOV system, which allows synchronized transport of more moderate cargo levels (Tago et al., 2025). Cargo trafficking behavior was essentially identical using these two approaches.

Tago, T., Ogawa, T., Goto, Y., Toyooka, K., Tojima, T., Nakano, A., Satoh, T. and Satoh, A. K. (2025). RudLOV is an optically synchronized cargo transport method revealing unexpected effects of dynasore. EMBO Rep 26, 613-634.

Third, we examined the localization of endogenous trafficking markers. The distribution of CI-M6PR and transferrin receptor (TfR) in GBF1^{M832L} cells was similar to that in wild-type cells (Figure for rebuttal 2A, B),

suggesting that major trafficking pathways remain intact in these cells. Furthermore, endogenous CI-M6PR localized to the TGN and endogenous Rab5 also localized to the early endosomes both in the presence and absence of nocodazole (Figure for rebuttal 1C, D). In addition, transferrin uptake experiments showed that internalized Tf reached NG::Rab11a-positive recycling endosomes within 20 min in both nocodazole-treated and untreated cells (Figure for rebuttal 1E, F).

Together, these results indicate that the major endosomal and Golgi trafficking pathways remain functional under our experimental conditions. However, since Reviewers 1 and 4 suggested reducing the number of results, these validation data were not included in the revised manuscript but are provided here for clarification.

REDACTED

REDACTED

Reviewers 1 and 4 recommended that we reduce the number of results, and we hope to use these data in the other manuscript

It would also be important to validate the sorting findings using endogenous cargo alongside a "rushed" cargo. Alternatively, looking at two endogenous cargoes via staining after BFA washout to confirm they are behaving similarly to the RUSHed cargoes would provide valuable validation.

We agree that validation using endogenous cargo would be valuable. However, visualization of the transport of newly synthesized cargo requires synchronized release from the ER, because the amount of newly synthesized protein is much lower than that of the pre-existing protein pool. For this reason, several systems for synchronized cargo release, including the RUSH system, have been developed and are widely used to study biosynthetic trafficking.

As an alternative approach, we attempted to analyze a cargo that cycles between the TGN and endosomes. Firstly, we investigated whether CI-M6PR is accumulated on TGN rather than Rab5-positive early endosomes by 90 min BFA-treatment (Figure for rebuttal 2A, B). Unfortunately, much of CI-M6PR is still in Rab5 compartments, probably because BFA-sensitive factor AP1 is necessary for CI-M6PR to recycle back to TGN.

REDACTED

REDACTED

We also examined CD-M6PR that had been labeled with a membrane-impermeable dye and found that it was rapidly endocytosed and transported to the TGN, followed by recycling to endosomes. Consistent with previous studies, this recycling from the TGN to endosomes was impaired in GGA mutants (we are currently preparing another paper for these results, and they are not shown). We therefore tested whether CD-M6PR could accumulate on the TGN by BFA treatment and subsequently be released upon BFA washout. However, under BFA treatment, most of endocytosed CD-M6PR did not reach the TGN well and accumulated Rab5-positive endosomes (Figure for rebuttal 2C). Again, this is likely because retrograde trafficking of CD-M6PR to the TGN requires BFA-sensitive factors, such as AP-1. Consequently, CD-M6PR is unlikely to be accumulated on the TGN under BFA treatment, making it unsuitable for the type of synchronized analysis performed in this study.

Reviewers 1 and 4 recommended that we reduce the number of results, and we hope to use these data in the other manuscript.

We confirmed the results obtained by RUSH using synchronized cargo-releasing method, RudLOV (Supplementary Fig. 8), which induces a limited amount of cargo (Tago et al., 2025).

Regarding the choice of HeLa cells, what was the rationale for this line, and is the system easily applicable to other cell types?

We chose HeLa cells, because many researchers in the field of membrane traffic use them.

To demonstrate the broad utility of the GBF1^{M832L} mutation, we created GBF1^{M832L} HEK293T cell line. The data obtained from the GBF1^{M832L} HEK293T cell line were included in the revised manuscript. Golgi absorption into the ER by BFA-administration was not observed in this cell (see Supplementary Video 1). Cargo transport stopped by BFA-administration and restarted with BFA washout in the GBF1^{M832L} HEK293T cell line (see Supplementary Figure 6g and Supplementary Video 8).

Figures 1b-c: For the development of the GBF M382L line, what is the viability of the mutant versus WT without BFA? Showing individual values in the Supplementary data, rather than just a ratio, would better establish cell health.

We have included the original data in a ZIP file named as "source data".

Figures 1j-k & Extended Data Figure 1: These require quantification of BIG1 and BIG2 colocalization at the Golgi. A quantified increase would reinforce the conclusions far more than the current images. Similarly, quantifying the colocalization of various ARFs with TfR or GalT in Extended Data Figure 1 is necessary. Some puncta seem to be colocalizing for ARF4 and ARF5 and GalT signal is hard to see.

Thank you for the nice suggestion. We have included graphs showing the quantified increase in colocalization between BIG1/BIG2 and GalT after BFA administration (see Fig. 1k and 1l): the results are much clearer with quantification. Additionally, we included graphs showing the Pearson's colocalization coefficient between ARF and TfR or Sec13 in the cytoplasm, excluding the GalT-positive region (the Golgi) and the nucleus. These graphs demonstrate ARF puncta colocalization on the RE (TfR) or the ERES (Sec13) (see Supplementary Fig. 1d, e).

Figures 1g,i,m,o: The quantification methods are unclear and the axes are currently missing labels. Some conditions show less than 100% signal at the Golgi peak, can the authors explain better how the data was expressed/normalized?

Thank you for your helpful comment. We have clarified the quantification method in the "Methods" section and added labels to the axes in Figures 1h, j, n, p, and Figure 5b, d, f, h, j, l.
The y-axis represents the average of the normalized signal intensity from 15 Golgi stacks. The peak signal intensity of each of the three proteins (Golgi markers and/or ARFs) was normalized to 100% within each stack. Then, the normalized profiles were averaged from 15 stacks. Therefore, the averaged peak values do not necessarily reach 100%.

Figure 2f: The data on Rab11 puncta mobility is interesting, but it lacks the statistics required to show if the observed effect is significant.

We apologize for the lack of statistics. We have included them in the revised Figure.

Figures 3c, f, and i: Same comments as above for Figure 1: The quantification methods are unclear and the axes are currently missing labels. Some conditions show less than 100% signal at the Golgi peak, can the authors explain better how the data was expressed / normalized?

The y-axis shows the average normalized signal intensity of the cargo in the Golgi stacks of 85 to 101 cells. The peak signal intensity of the cargo in the total Golgi region (GalT::iRFP) was normalized to 100% in each cell, and then the normalized profiles were averaged across the 85-101 cells. Therefore, the averaged peak values do not necessarily reach 100%.

Figure 3f: Some tubules appear to be devoid of Rab11 signal. Are these tubules cargo-dependent, or do they represent distinct types?

In addition to Fig. 3l–n, we have now included analyses of all three combinations of the cargo types and Rab11a in Supplementary Fig. 5b–d. These analyses demonstrate that the three cargo types are present in the same tubules. However, only a subset of these tubules are Rab11-positive, and many Rab11-positive tubules are observed without detectable cargo. Notably, cargo-containing tubules only appear when cargo exits the Golgi apparatus, while Rab11-positive tubules appear shortly after BFA administration. Therefore, we suggest that the difference in the timing of tubule formation between cargo and Rab11 likely reduces their apparent colocalization.

Lines 198-200: The statement regarding the separation of GPI-AP and VSVG needs quantification to support the observation that VSVG and TNFα continue to co-localize.

We quantified the colocalization of VSVG with GPI-AP and TNFα during transport following BME treatment (Supplementary Fig. 5g). The Pearson's correlation coefficient between VSVG and GPI-AP decreased rapidly after the onset of transport, whereas the coefficient between VSVG and TNFα remained unchanged throughout, indicating sustained colocalization.

Lines 201-202: For the claim about M6PR transport, it isn't clear if this is the authors' own data or a reference to previous studies; if the latter, it needs to be cited.

We added the following references.

*Coutinho, M. F., Prata, M. J. and Alves, S. (2012). Mannose-6-phosphate pathway: a review on its role in lysosomal function and dysfunction. Mol Genet Metab **105**, 542-50.*

Figure 5 & Figure 7: These sections suffer from a lack of quantification and statistical analysis, which needs to be addressed to substantiate the claims

We quantified all the images in Figure 5 and displayed the results at the bottom of the figure. The results showed that ARF1/3 accumulates gradually on the Golgi, while GPI-AP leaves the Golgi after BFA washout. We also quantified the images in Figures 7b and 7d, shown in Figures 7c and 7e, respectively. Figure 7c shows that, after BFA washout, ARF1/3 and AP-1 gradually accumulate on the Golgi. Most importantly, quantifying the GPI-AP- or CD-M6PR-positive area within the AP-1-positive regions of interest (ROIs) near the Golgi stacks shows that AP-1 colocalizes with GPI-AP but not CD-M6PR. The degree of colocalization differs significantly between those two (Figure 7e).

Figure 5o, Why are the two ARF (1 and 3) behaving differently when observed by themselves (o) vs with cargo (m, n)? There seems to be a loss of both ARFs for a longer period of time. Again, the lack of quantification makes it difficult to assess.

We quantified the amount of ARFs after BFA washout and included the results in Figure 5r-t.

The difference in ARF accumulation timing near the Golgi stacks may be due to variations in BFA incubation time and cargo induction. In Figure 5m and 5n, GPI-AP transport from the ER was induced by the BME-RUSH system, while GPI-AP exit from the TGN was blocked by BFA. After a 60-minute incubation period with BFA, the drug was washed out, and the cells were imaged. ARF1 and ARF3 quickly and massively returned to the TGN. In contrast, in Figure 5o, RUSH-cargo was not expressed and the BFA incubation time was much shorter (10 minutes). The presence of cargo may be a key factor in ARF1/3 in the TGN.

There seems to be a loss of both ARFs for a longer period of time.

Not ARFs (green) but GPI-AP (red) lost for a longer period of time: GPI-AP is transported from TGN to the plasma membrane after BFA-washout, thus it is lost for a longer period of time.

Minor Points:

Lines 124-125: The description of ARF1 "disappearing on the puncta" is confusing. How does this differ from the diffusion seen with ARF3?

It is actually the diffusion of ARF1 from the puncta. We rephrased the sentences as follows:
"Upon BFA administration, all of ARF3 diffused, and ARF1 on the puncta also diffused; however, ARF1 on the Golgi apparatus remained visible."

Line 183: Possible typo; should this read "ARF1"?

Thank you. We fixed.

Lines 241-242: The wording about two types of cargoes being close to each other is vague, especially as only GPI-AP was mentioned in that paragraph.

We added the following sentences in current Line 296-297:

"To investigate whether different types of cargo accumulate separatory we examined the two types simultaneously."

The manuscript would benefit from a clearer transition for the investigation of AP-1 and clathrin. What is the rationale for looking at AP-1?

Using electron microscopy, we found GPI-AP-containing clathrin vesicles 15 minutes after BFA washout. Therefore, we wonder if clathrin and its adaptor are involved in GPI-AP trafficking. AP-1 and GGA are well-known clathrin adapters that locate in the TGN; however, GGA is also known to be involved in endosomal trafficking rather than transport to the plasma membrane. Therefore, we focused on AP-1. We added the following clause to the text in current lane 347-348:
"As clathrin-coated vesicles or buds containing GPI-AP were observed in electron microscopy,"

Additionally, the standard "AP-1" (with a hyphen) nomenclature should be used throughout.

We changed "AP1" to "AP-1".

The use of the term "natural conditions" (line 299 and throughout) is inaccurate for a system involving genetic mutations, chemical inhibitors, and light-induced cargo release.

We agree with the reviewer's suggestion to change the following sentence in current Lane 362-363.
"Finally, AP-1 and cargo localization were examined using RudLOV, a new light-induced cargo-releasing system that can control the amount of cargo released from the ER."

There is a typo in Extended Data Fig1: "e, f ARF::NG4 (e) " it should be ARF4::NG.
We fixed.

Same figure, maybe another typo in “The plots show the normalized mean values for 15 Golgi markers and ARF line profiles across the Golgi stack “ 15 seems to be not relevant.

We used 15 profiles rather than 15 Golgi markers. To make clearer, we changed “15 Golgi markers and ARF line profiles” to “15 ARF and Golgi markers line profiles”.

Typo in Extended Data Fig3: AFR1 should be replaced with ARF1 throughout.
We fixed.

Reviewer #3 (Remarks to the Author):

By using BFA treatment in the newly generated GBF1M832L cell line and powerful live images, Arata Takiguchi et al established a unique system to manipulate and observe the post-Golgi transport of cargo trafficking in this manuscript. Previous study has reported that BFA-sensitive Sec71 regulates RE detachment from Golgi stacks and post-Golgi transport in Drosophila. Based on this, the authors constructed a BFA-resistant GBF1 allele (GBF1M832L) in HeLa cells to analyze GPI-AP transport from TGN to GA-RE. They further validated that Sec71 homologs (BIG1/2) specifically activated ARF1/3, but not ARF4/5. Meanwhile, they carefully examined RE motility in MT-intact or -disrupt conditions, and compared 3 different cargoes (GPI-AP, VSVG and TNFα) transport in this system. Finally, the authors provided the evidence that AP-1 was required for microtubule-independent post-Golgi transport through a tubule scission mechanism.
Overall, this study provided a detailed investigation of RE-mediated cargo transport by the impressive images of electronic microscope and 3D-rendered model. However, several issues regarding to the chemical treated assay remain to be addressed.

Major concerns:

1. This study used BFA to treat cell line. Although the data is interesting and impressive, the whole process is unnatural. Additional evidence, including interfering key factors of BFA targets by knockout assay, should be given to validate the data.

We agree that it is important to verify that the phenotypes observed after BFA treatment reflect inhibition of its target factors. Because BIG1/2 double knockout is expected to be cell lethal from our Drosophila work (Fujii et al., 2020 JCS PIMD33262309), we instead generated BIG1/2 double knockdown (DKD) cells using siRNA. Seventy-two hours after start of siRNA treatment, the protein levels of BIG1 and BIG2 were reduced to 17.5% and 18.6% of those in wild-type cells (Supplementary Fig. 3a). Under these conditions, Rab11-positive tubular networks were not always but occasionally formed in BIG1/2-DKD cells (Supplementary Fig. 3b). We further analyzed the dynamics of recycling endosomes (REs) and Golgi stacks in microtubule-disrupted BIG1/2-DKD cells and observed a significant reduction in RE motility (Supplementary Fig. 3c-e). In addition, electron microscopy showed vesicle cluster accumulation in trans-side of Golgi stacks (opposed to ERES shown in white arrowheads) in MT-disrupted BIG1/2-DKD cells (Supplementary Fig. 3f). Some of these vesicles are in hemi-fused appearance (Supplementary Fig. 3f yellow arrows).

We also examined GPI-AP transport in both MT-intact and MT-disrupted BIG1/2-DKD cells (Supplementary Fig. 6a-f). Similar to be observed in BFA-treated GBF1^{M832L} cells, cargo transport was strongly delayed in MT-intact cells. However, cargo transport was strongly delayed but not blocked in MT-disrupted BIG1/2-DKD cells, although cargo transport was totally disrupted in MT-disrupted BFA-treated GBF1^{M832L} cells. This milder phenotype of MT-disrupted BIG1/2-DKD cells than that of MT-disrupted BFA-treated GBF1^{M832L} cells must be the residual activity of BIG1/2 in MT-disrupted BIG1/2-DKD cells.

Together, these results indicate that the phenotypes observed in BFA-treated GBF1^{M832L} cells are recapitulated by depletion of BIG1/2, supporting the conclusion that they arise from inhibition of BIG1/2 activity.

2. The authors performed many live-cell images. However, some images or videos quality are dubious: 1) in supplementary video 1, the signals were not clear due to low resolution; 2) in figs 1k and 2b, GalT::iRFP+ signals, as a marker of trans-Golgi network, should not present in extracellular matrix. These images should be revised or replaced with higher-quality, representative examples.

1) We revised Supplementary Video 1 with a new, high-resolution live imaging movie.

2) GBF1^{M832L} cells that constitutively express GalT::iRFP were transfected with some additional markers, and imaged. Thus, the neighboring cells in the Figures usually express only GalT::iRFP. Therefore, you can see the neighboring cells expressing only GalT::iRFP in current Fig 1l.

3. The authors constructed a single allelic GBF1M832L HeLa cell line by using CRISPR/Cas9-mediated knock-in method. However, the detailed information of this cell line was lacking, such as knock-in site, GBF1 protein level.

The detailed information on constructing the GBF1^{M832L} HeLa cell line was included in the experimental data sets; however, it does not seem easy to find. Thus, we added guidance to the Methods section, where the information is available. We performed an immunoblot analysis to investigate the amount of GBF1 in wild-type and GBF1^{M832L} HeLa cells and found that they are comparable (see Fig. 1b).

Meanwhile, the authors should explain the reason of GBF1M832L cells were more resistant to BFA treatment.

In the first paragraph of the section “Construction of BFA-resistant GBF1M832L cells” in Methods, we explained why GBFM832L is BFA-resistant.
”BFA inhibits ARF protein activation by forming dead-end complexes with ARF-GDP and ARFGEF (Peyroche et al., 1999; Renault et al., 2003). BFA-resistant ARFGEFs differ from BFA-sensitive ones by a few amino acid residues in their GDP/GTP exchange domains. Introducing a BFA-resistant residue into a BFA-sensitive ARFGEF converts it into a BFA-resistant ARFGEF and vice versa (Geldner et al., 2003; Robineau et al., 2000). Methionine at position 832 (M832) in GBF1 is a key residue for BFA binding and replacing it with leucine (L) converts GBF1 into a BFA-resistant form. The GBF1-specific inhibitor GCA binds to wild-type GBF1 but not to GBF1M832L (Sáenz et al., 2009).”

4. BFA treatment paradigm is foundational for this study. Hence, the choices of specific time points (10, 30, 60 min) for different experiments should be justified.

We selected a 60-minute BFA treatment to perfectly synchronize cargo transport by blocking and restarting cargo exit from the Golgi. To confirm that the Golgi marker distribution was normal after adding BFA, we used a 60-minute period. When we investigated their BFA sensitivity, we used shorter periods for sensitive ARFs and BIG1/2 and longer periods for resistant ARFs.

5. The manuscript lacked basic background introduction in many cases: 1) the reason of using MT-intact and MT-disrupt cells in this assay, because Golgi apparatus was totally fragmented in MT-disrupt cells, whether the results were convinced in these conditions; 2) the effect of GCA treatment on cells; 3) in the final part of the manuscript, the logical flow in the investigation of AP-1 feels slightly disconnected. Introducing a hypothesis or previous study is recommended.

1) In our original manuscript, we explained why we used MT-disrupted cells in current line 111-114 "MT-disrupted GBF1^{M832L} cells, which have scattered Golgi stacks in front of each ER exit site (ERES) instead of the Golgi ribbon, were used to further investigate the cis–trans polarity of the Golgi apparatus."
However, we did not mention why we used MT-disrupted cells at the beginning of transport assay using MT-disrupted cells in our original manuscript. Thus, we added the following sentence in line 242-244, "Next, we investigated the effects of BFA on GPI-AP transport in MT-disrupted GBF1^{M832L} cells, because the scattered Golgi stacks allow for a more detailed analysis with precise cis–trans polarity."

2) We added the following explanation of GCA in current lane 96-98: "As the GBF1-specific inhibitor Golgicide A (GCA) binds to wild-type GBF1 but not to GBF1^{M832L} (Sáenz et al., 2009), we investigated the effect of GCA on the viability of the wild-type and GBF1^{M832L} cells."

3) For AP-1, we included the following clause at the beginning of AP-1 section in current lane 347-348: "As clathrin-coated vesicles or buds containing GPI-AP were observed in electron microscopy."

6. The author should utilize this system in other mammalian cells to expand its broad utility.

To demonstrate the broad utility of GBF1^{M832L} mutation, we created GBF1^{M832L} HEK293T cell line. The data obtained in GBF1^{M832L} HEK293T cell line were included in revised manuscript. Golgi absorption into the ER was not observed by BFA-administration (Supplementary Video 1) and cargo transport stopped by BFA-administration and restarted by BFA-washout in the GBF1^{M832L} HEK293T cell line (Supplementary Fig. 6g and Supplementary Video 8).

Minor concerns:

1. The authors should add descriptive titles to all supplementary videos to enhance their clarity.

We add a title frame to all supplementary videos.

2. In some images, such as figs 1d and 1e, the authors use same color (gray) to label different signals was confusing. Using different colors to label distinct Golgi networks is recommended.

Thank you. We fixed.

3. The authors should modify some images: 1) figs 1d and 1j should be clearly labeled to indicate which panels represent MT-intact or MT-disrupted conditions;

We labeled "nocodazole +" for MT-disrupted cells in Figures.

2) the authors should add boxes or arrowheads in left panels to identify the regions shown at higher magnification on the right panels in fig 1f-i.

The Golgi stacks in the left images in Fig. 1h and 1j were not presented in the panels (g, i). The plots are the averages of 15 Golgi stacks, and the images of the Golgi stacks in the left images (h, j) are representative.

4. It would be extremely helpful for readers if the authors draw a schematic work model to summarize this work.

We added a schematic video (Supplementary Video 19) and a graphical abstract to explain our model of post-Golgi transport of GPI-AP via RE.

5. Line 53, please extensively introduce the information of "other pathways".

We apologize for this grammatical mistake. We just wanted to clarify that the Golgi apparatus and REs are primarily involved in exocytic and endocytic pathways, respectively. However, both organelles also participate in other pathways. The Golgi apparatus is involved in the endocytic pathway, and the REs are involved in the exocytic pathway. Therefore, we changed the sentence as follows:

"The Golgi apparatus and REs are primarily involved in the exocytic and endocytic pathways, respectively; however, each organelle also contributes to the other pathway."

6. Line 183, the "AFR1" should be modified into "ARF1".

We fixed.

7. Line 229, "but not significantly different". The data should be "fig 4f". please examine this result.

We are sorry. This was our big mistake. The correct data is "Fig. 4g." However, the difference is significant. Thus, we changed the sentence as follows:

"The time constant of GPI-AP exit from the Golgi stacks was calculated using this peak, which was slightly but significantly slower than ~~but not significantly different from~~ the one without BFA (Fig. 4g)."

Reviewer #4 (Remarks to the Author):

This manuscript by Takiguichi and colleagues has investigated the trafficking of cargo from the TGN and especially the functional relationship between the TGN and recycling endosome during cargo transport. The work is certainly of interest to understand cargo transport and Golgi exit along the anterograde transport pathway. The establishment of a cell system to allow brefeldin A controlled cargo exit from Golgi stacks is a clever approach that allows the exit of cargo from the TGN to be investigated.

However, I found the manuscript very dense with information and it was challenging to identify the key findings from the manuscript that represent the advance from the previous work by the Satoh laboratory (Fujii et al 2020, JCS). The major take home message from the exploitation of this cell system was not entirely clear to this reviewer. A cartoon depicting the new findings and conclusions would have been helpful. I think the paper could be dramatically shortened to enhance the readability of the paper and to focus on the key findings.

We added a schematic Video (Supplementary Video 19) and a graphical abstract to explain our model of post-Golgi transport of GPI-AP via RE. We also removed original Extended Data Figure 3 to make the manuscript shorter.

I had a number of specific concerns/issues relating to the clarity of the data, quantitation, and interpretation.

Additional concerns

1. An ER control marker is needed Fig. 1d.

We revised current Fig 1e to include an ER marker.

2. Fig. 1h, 1i. The manuscript says that the “ localisation of golgin 97 remained unaffected”. One cannot conclude this from the optical images, but could say the relative spatial distribution of GM130. GalT and golgin 97 was very similar in BFA treated and untreated cells.

Thank you for your helpful suggestion. We have revised the sentence as you suggested.

3. Line 105/106 states “ which have scattered Golgi stacks in front of each ER exit site….” What is the evidence for this conclusion?

Dr. Hammond and Glick first showed ERES (they called transitional ER) is in front of Golgi in nocodazole-treated mammalian cells. Dr. Lu’s group also have clearly demonstrated. We cited both references in our revised manuscript.

Hammond, A.T. & Glick, B.S. Dynamics of transitional endoplasmic reticulum sites in vertebrate cells. Mol Biol Cell 11, 3013-3030 (2000).

Tie, H. C., Mahajan, D. and Lu, L. (2022). Visualizing intra-Golgi localization and transport by side-averaging Golgi ministacks. J Cell Biol 221.

4. Data on the localisation ARF4 and ARF5, line 121 and following, is contrary to some of the literature- at least for ARF4. W. Nakai, Y. Kondo, A. Saitoh, T. Naito, K. Nakayama, H.W. Shin, ARF1 and ARF4 regulate recycling endosomal morphology and retrograde transport from endosomes to the Golgi apparatus, Mol Biol Cell 24 (2013) 2570-2581. Also, ARF4 is a binding partner of Rab30, which is localised to the TGN and recycling endosomes (Zulkefli et al Exp Cell Res, 399 (2021):112442). These differences in the literature need to be reconciled.

We agree with the reviewer and have added this information, along with references, to the text as follows:

“These results suggest that ARF1 and ARF3 are prominent targets of BIG1/2, although some previous studies have indicated that ARF4 is also localized to the TGN and regulates trafficking from endosomes to the TGN (Nakai et al., 2013; Zulkefli et al., 2021).

5. In Fig. 2b, Rab11- labelled tubules are hard to see in BFA-treated cells.

We revised Fig 2b. To demonstrate the clear, Rab11-positive tubule, we used Z-projection images created from three slices with an interval of 0.4 μm.

6. Fig. 2C- quantitation of attachment and separation of Rab11-positive structures from Golgi structures should be provided, as this represents an important dataset for the conclusion.

We quantified the attachment and detachment events of Rab11-positive recycling endosomes (REs) to and from the Golgi stacks using the same movies that were used for the analysis of RE motility (Fig. 2g and Fig. 7j). These analyses have now been included in Fig. 2d and Supplementary Fig. 9j. The results show that both BFA treatment and AP-1 deficiency cause a marked reduction in the number of attachment and detachment events of REs to and from the Golgi stacks, in addition to the reduction in RE motility.

7. Fig. 2g, 2h: how is the polarity of the Golgi determined in the EM images to conclude that the vesicle accumulation was on the trans side? For BFA treated samples it is not clear how the Golgi membranes were identified as the Golgi stack is greatly perturbed.

We believe the polarity of the Golgi stacks is evident in Fig. 2h and 2i, because the images include the ERES, as indicated by the arrowheads in the revised Figure 2. While we agree that the Golgi stacks are swollen and distorted in BFA-treated cells, they still maintain their cisternae and association with the ERES. Thus, we can easily identify them as Golgi stacks.

How does the perturbation of the Golgi stack impact on the proposed conclusions of the paper?

BFA causes the Golgi stack to swell. However, we also know that the Golgi stack looks normal 15 minutes after BFA washout, and post-Golgi transport proceeds with normal kinetics after BFA washout. Therefore, although we cannot say that the Golgi is not influenced by BFA, the benefits of this system are tremendous and have greatly helped us reach the proposed conclusions.

8. Line 182: Please clarify why GCA did not alter the kinetics of GPI-AP transport- this reviewer found this confusing. GCA should inhibit ERES trafficking?

Golgicide A (GCA) is a GBF1-specific inhibitor, that binds to the wild-type GBF1 but not to GBF1^{M832L} (Sáenz et al., 2009). We used GBF1^{M832L} cells, which contain both the wild-type and GBF1^{M832L} alleles. GBF1^{M832L} does not bind to GCA; however, the wild-type GBF1 forms a complex with ARF and GCA. When half of the GBF1 function (wild-type) is inhibited, the other half (GBF1^{M832L}) remains active. Thus, GCA does not alter the kinetics of GPI-AP transport.

9. Lines 192-194. Conclusion that “numerous Rab11 + tubules simultaneously carried newly synthesised cargo” was clear for the GPI-AP cargo but not obvious for the other cargoes. Better images are needed.

In addition to Figure 3l-n, we have included data on all combinations of the three cargo types and Rab11a in Supplementary Figures 5b-d. All three cargo types are localized in the same tubules: we investigated three combinations of two cargoes. However, we found that only some of them are Rab11-positive, and many Rab11 tubules are found without cargo. Cargo-laden tubules only form when cargo exits the Golgi. However, Rab11-positive tubules form shortly after BFA administration. Rab11-positive tubules form much earlier than cargo-containing tubules. We hypothesize that the difference in the timing of tubule formation between cargo and Rab11 reduces their colocalization.

10. Line 201- “M6PR was transported to endosomes rather than plasma membrane:” This finding may be expected but do the authors have data that this is the case in their manipulated cell system? No data appears to have been provided.

We added the reference as follows:

> *Coutinho, M. F., Prata, M. J. and Alves, S. (2012). Mannose-6-phosphate pathway: a review on its role in lysosomal function and dysfunction. Mol Genet Metab* ***105****, 542-50.*

We have data on the transport of newly synthesized CD-M6PR to endosomes in our manipulated cell system. We found that CD-M6PR is transported from the TGN to the Golgi-attached Rab5 compartment (Figure for rebuttal 3). We are preparing another manuscript on this topic. The arrows show the Rab5 compartment gradually accumulating CD-M6PR after BFA washout.

REDACTED

11. Fig extended data Fig. 3a-f are very small and difficult to interpret.

The data in Extended Data Fig. 3a-f are not very important for our story. We removed them.

12. Figure 6B. CLEM is weak.

We appreciate the reviewer's concern that the CLEM analysis may not provide sufficient resolution to clearly identify the membrane structures associated with the fluorescence signal. Indeed, CLEM has an intrinsic limitation because the resolution of light microscopy is substantially lower than that of electron microscopy, making it difficult to unambiguously assign fluorescence signals to specific membranes. To address this limitation, we performed additional experiments using TfR::APEX2 (APEX2 in lumen), which allows direct visualization of recycling endosomes (REs) at the ultrastructural level. Our new results reinforced some of accumulated vesicles near Golgi stacks are APEX2::TfR positive, which is the direct evidence that at least some of vesicles in vesicle clusters are REs. We also successfully showed that the beads-like structures connecting to TGN in the wild type cells and the long-tubules connecting TGN in AP-1 deficient cells are APEX2::TfR positive. Thus, GPI-AP containing membrane emerged from TGN is REs. These new data have been included in the revised Supplementary Figure 4 and Supplementary Figure 12 and further support our conclusions.

Moreover, we finally succeeded in the double labelling of APEX2::GPI (APEX2 in lumen) and APEX2::TfR (APEX2 in cytoplasm). These data gave the direct evidence, showing that GPI-AP containing tubules or beads on the string structures are REs (Figure 6f, g and Supplementary Figure 7c-e).

13. Figure 7e; quantitation is needed on the level of co-localisation of AP1 with GPI-AP and M6PR.

We assume that your suggestion refers to the quantification of the co-localization between AP-1 and GPI-AP/M6PR shown in our original Fig. 7c and d. As the original Fig. 7c and d presented essentially the same results obtained from different samples, we have removed the image in Fig. 7d. Instead, we now include a graph showing the quantification of the GPI-AP– or CD-M6PR–positive area within AP-1–positive ROIs near Golgi stacks. The results clearly demonstrate that AP-1 is strongly co-localized with GPI-AP, but not with CD-M6PR.

14. Extended Fig 5a- says cargo enters the cis side of Golgi, however, a cis-Golgi marker is not included. Also data at 9 min pulse is not shown in Supp Fig. 5, but is mentioned in text

We did not use the cis-Golgi marker, but we did use the TGN marker Cv::Rab6a and the trans-Golgi marker GalT::iRFP. Therefore, the cis-trans polarity of the Golgi stacks was evident. We apologize that the data from the nine-minute time point was not included in original Extended Data Figure 5a (current Supplementary Figure 8a). In this figure, we only showed frames taken every six minutes. Please refer to Supplementary Video 14 for the other time points.

15. Line 336. It was not clear to this reviewer why KO of AP-1 did not result in the accumulation of GPI-AP in the Golgi stacks (Fig. 7n-q).

As the reviewers pointed out, GPI-AP exit from the Golgi stacks was delayed but not totally blocked in AP-1-KO cells. We interpret these results as indicating that AP-1 is not involved in the anterograde transport of GPI-APs. Currently, we hypothesize that AP-1 is involved in the retrograde trafficking of non-GPI-AP proteins from the RE/TGN to late Golgi compartments. GPI-APs are transported from the TGN to the RE. However, this sorting is not tightly regulated; many TGN and Golgi resident proteins are transported to the RE with GPI-APs and AP-1-dependent retrograde transport then salvages these proteins from the RE. This hypothesis is

consistent with recent studies indicating AP-1 functions on retrograde trafficking (Casler et al., 2022; Robinson et al., 2024).

Casler, J. C., Johnson, N., Krahn, A. H., Pantazopoulou, A., Day, K. J. and Glick, B. S. (2022). Clathrin adaptors mediate two sequential pathways of intra-Golgi recycling. J Cell Biol 221.

Robinson, M. S., Antrobus, R., Sanger, A., Davies, A. K. and Gershlick, D. C. (2024). The role of the AP-1 adaptor complex in outgoing and incoming membrane traffic. J Cell Biol 223, e202310071.

We think the formation of clathrin/AP1-coated vesicles results in the scission of RE tubules from the TGN. Therefore, in AP-1-KO cells, RE tubules do not disconnect from the TGN, and GPI-AP exit from the Golgi stacks is delayed. In fact, GPI-AP accumulates for a longer period in the Golgi stacks of AP-1 KO cells than in wild-type cells (see Fig.7p, r). We added a schematic Video (Supplementary Video 19) and a graphical abstract to illustrate our model of the post-Golgi transport of GPI-AP via the RE.

16. Line 342 and Fig. 8: GPI-AP incorporation into recycling endosomes of AP1 KO cells after BFA washout was not convincing. There is very little overlap of green and red signals, just proximity.

As noted in the first paragraph of this response, we accidentally mislabeled HT::GPI-JF549 and NG::Rab11a in the previous version of Figure 8. HT::GPI-JF549 is in fact shown in red, whereas NG::Rab11a is shown in green. We believe this labeling error may have contributed to the reviewer's concern.

To show the overlap of GPI-AP and Rab11 clearly, we extracted pixels containing both GPI-AP and Rab11 signals and showed them in yellow in the bottom panel (see Figure for rebuttal 4). The resulting signal closely matches the Rab11 distribution, indicating substantial overlap. Because this result does not provide additional information beyond the Rab11 signal itself, we did not include it in the main Figure.

Figure for rebuttal 4

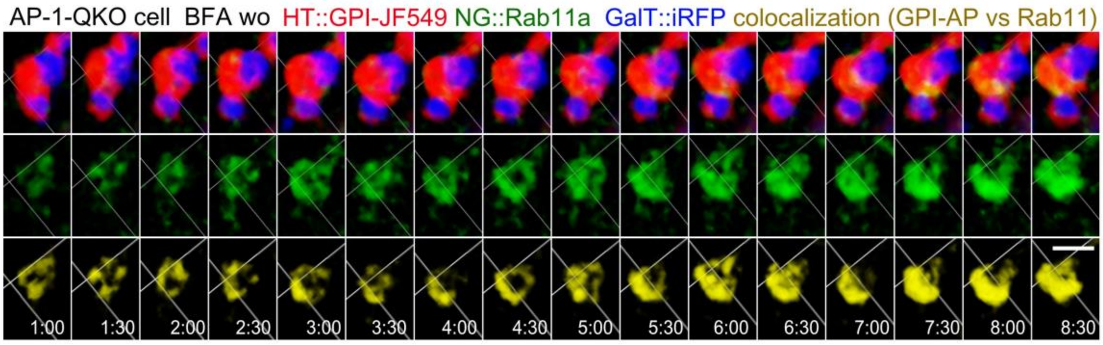

Reviewer #2 (Remarks to the Author):

The authors have appropriately addressed my comments and concerns. The quantification of the key assays is a major addition to the manuscript.

Reviewer #3 (Remarks to the Author):

Takiguchi et al. performed addtional experiments and revised the manuscript to well addressed all my concerns. I recommend the publication of this mansucript.

Reviewer #4 (Remarks to the Author):

The revised manuscript by Satoh and colleagues has provided detailed responses and addressed my concerns and suggestions. Quantitation has been significantly improved. The manuscript remains very dense with data, however, the information is required to validate the conclusions. Overall, an impressive body of work.

There are a few minor issues that need to be corrected.
1. Figure 1e no longer has GM130 as a marker in the revised manuscript. The text in results section and in the Figure legend need to be corrected, and replaced with KDEL.

Thank you for the helpful suggestion.
However, we did not find the label of GM130 in Figure1e and in the text described about Figure 1e.
It seems they have already changed to KDEL.

2. Line 107-108. The statement that the “Golgi ribbon was slightly fragmented” cannot be concluded based on the resolution. It is more appropriate to state that the Golgi morphology was slightly perturbed.

We agreed. We fixed the sentence.

3. Video 19 has been added in the revision to explain the model. Reference to this video is missing in the text and needs to be included.

We cited Video19 in the discussion.
